# The temperature-dependence of host–guest binding thermodynamics: experimental and simulation studies†

**DOI:** 10.1039/d3sc01975f

**Published:** 2023-10-13

**Authors:** Laura M. Grimm, Jeffry Setiadi, Boryslav Tkachenko, Peter R. Schreiner, Michael K. Gilson, Frank Biedermann

**Affiliations:** a Institute of Nanotechnology (INT), Karlsruhe Institute of Technology (KIT) Hermann-von-Helmholtz Platz 1 76344 Eggenstein-Leopoldshafen Germany laura.grimm@kit.edu frank.biedermann@kit.edu; b Skaggs School of Pharmacy and Pharmaceutical Sciences, University of California, San Diego 9255 Pharmacy Lane La Jolla CA 92093 USA mgilson@health.ucsd.edu; c Institute of Organic Chemistry, Justus Liebig University Giessen Heinrich-Buff-Ring 17 35392 Giessen Germany

## Abstract

The thermodynamic parameters of host–guest binding can be used to describe, understand, and predict molecular recognition events in aqueous systems. However, interpreting binding thermodynamics remains challenging, even for these relatively simple molecules, as they are determined by both direct and solvent-mediated host–guest interactions. In this contribution, we focus on the contributions of water to binding by studying binding thermodynamics, both experimentally and computationally, for a series of nearly rigid, electrically neutral host–guest systems and report the temperature-dependent thermodynamic binding contributions Δ*G*_b_(*T*), Δ*H*_b_(*T*), Δ*S*_b_(*T*), and Δ*C*_p,b_. Combining isothermal titration calorimetry (ITC) measurements with molecular dynamics (MD) simulations, we provide insight into the binding forces at play for the macrocyclic hosts cucurbit[*n*]uril (CB*n*, *n* = 7–8) and β-cyclodextrin (β-CD) with a range of guest molecules. We find consistently negative changes in heat capacity on binding (Δ*C*_p,b_) for all systems studied herein – as well as for literature host–guest systems – indicating increased enthalpic driving forces for binding at higher temperatures. We ascribe these trends to solvation effects, as the solvent properties of water deteriorate as temperature rises. Unlike the entropic and enthalpic contributions to binding, with their differing signs and magnitudes for the classical and non-classical hydrophobic effect, heat capacity changes appear to be a unifying and more general feature of host–guest complex formation in water. This work has implications for understanding protein–ligand interactions and other complex systems in aqueous environments.

## Abstract 2

Through isothermal titration calorimetry (ITC) and molecular dynamics (MD) simulations, we demonstrate that negative changes in heat capacity (Δ*C*_p,b_) are a unifying feature for both the classical and non-classical hydrophobic effect.

## Graphical abstract

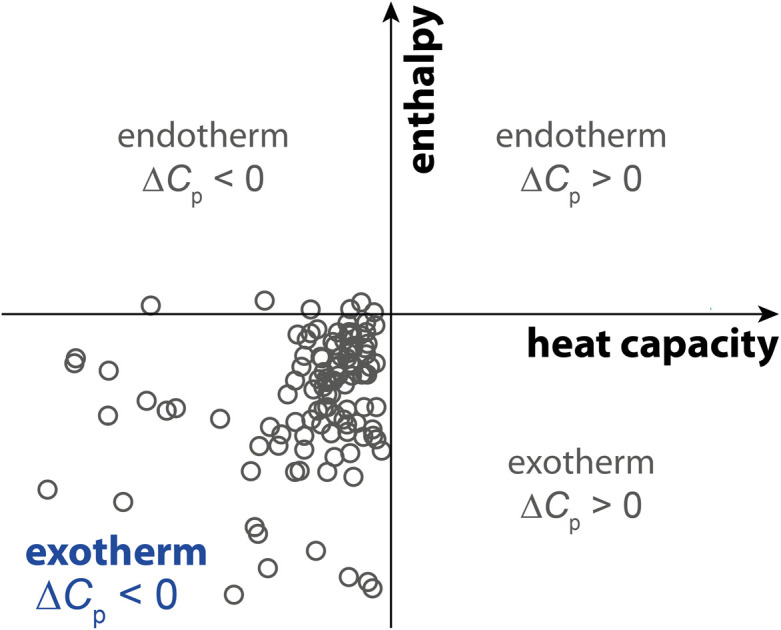

## Introduction

Understanding the principles of molecular interaction in aqueous solutions is crucial for biochemical and pharmacological research, as it provides a basis for the rational design of targeted compounds, including proteins and small molecule drugs.^1–3^ Water as a solvent has complex and non-intuitive features. It is challenging to distinguish its contributions to binding thermodynamics from direct solute–solute interactions.^4–8^ Studying simple systems with a limited number of atoms, such as aromatic systems like benzenes, can help address this issue.^9,10^ However, available experimental methods do not provide a detailed structural picture of the stacking geometries of aggregated aromatic molecules in water, and the heterogeneity of the aggregates cannot be assessed. Moreover, it is cumbersome to obtain all thermodynamic parameters – binding free energy Δ*G*_b_, binding enthalpy Δ*H*_b_, binding entropy Δ*S*_b_, and changes in heat capacity Δ*C*_p,b_ experimentally, as the binding interactions are relatively weak, and isothermal titration calorimetry (ITC) cannot easily be applied to such systems. In contrast, processes involving biomolecules, such as the binding of small molecule ligands and drugs with proteins or nucleic acids, can routinely be measured and their binding and folding geometries are usually relatively well-defined, and often follow a two-state model.^11,12^ Frank and Evans' iceberg model of hydrophobic hydration led Kauzmann to postulate that an entropic driving force for intramolecular binding is generated by the liberation of structured surface water molecules around apolar peptide side chains during protein folding.^13,14^ However, the concept of entropy as the driving force for hydrophobic association *via* water liberation has never been unequivocally accepted, and the debate continues to this day.^15,16^ For instance, Ben-Naim^17^ and Baldwin^18^ expressed opposing views in their respective 2013 and 2014 articles on whether the association of non-polar solutes is driven in part by water contributions. Ben-Naim challenged the dominance of the hydrophobic effect and introduced the concept of hydrophilic effects as crucial factors in processes such as protein folding and protein–protein association.^17,19,20^ Conversely, Baldwin argued that the concept of dynamic hydration shells restores Kauzmann's explanation of how the hydrophobic factor drives protein folding.^18,21,22^ Such discrepancies may partly trace to the complexity of biomolecules and the number of counteracting effects in play when they bind or fold. For instance, the role of structured water molecules compared to “water reservoirs” and completely “dewetted” regions in the binding pockets or on the surface of biomacromolecules still requires further investigation despite the excellent contributions of many research groups, *e.g.*, Berne,^23,24^ Whitesides,^5,25^ Diederich,^4,26^ Klebe,^27,28^ and Grzesiek.^29^

Host–guest systems are more straightforward models for studying hydrophobic and non-covalent interactions because they are dramatically simpler than proteins and nucleic acids. They are also of practical interest in their own right, as they have applications as pharmaceutical excipients,^30–33^ toxin scavengers,^34^ building blocks for synthetic receptors and chemosensors,^35,36^ and components of self-healing and smart materials.^37–41^ Host–guest binding data are also used extensively to evaluate the accuracy of computational methods, notably in the SAMPL series of blinded prediction challenge.^42–48^ The empirical investigation of molecular studies and host–guest complexes has already led to suggested correlates and principles of binding affinity, such as the packing coefficient,^49–51^ the energetic cost of receptor organization,^52–54^ entropy,^55–58^ conformational freedom and effective molarities,^59,60^ multivalency,^61–64^ the molecular electrostatic potential surface,^65^ surface site interaction points (SSIP),^66^ the solvent cohesiveness,^67–69^ the Hofmeister and chaotropic effect,^70–72^ the solvent accessible surface area,^73^ differential cavitation energies,^74^ and the high-energy water release concept.^75–78^ Although such concepts are intuitive and help guide the design of improved host–guest pairs, they do not directly provide insight into the thermodynamics of binding and are not necessarily comprehensive or mutually exclusive.

A particularly powerful way to gain insight from host–guest binding is to combine experimental and computational approaches. For example, room-temperature ITC data and DFT-computed energy contributions revealed that dispersion energy and packing coefficients are not decisive factors in aqueous CB*n*·guest binding.^79^ Note, however, that implicit solvation models, such as COSMO, may introduce sizable errors in solvation contributions for these systems, as they may not be highly suited to describe solvents in the confined interior of a host molecule.^79^ Molecular dynamics (MD) simulations with explicit solvent consideration may be more useful in this regard. Thus, a combined experimental and simulation study explored the dewetting of host molecules with nonpolar cavities,^80^ and explicit solvent simulations were used to compute binding enthalpies of CB*n*·guest systems and dissect them into defined contributions, such as the changes in solute–solute, solute–water, and water–water interaction energies.^81,82^

The present study builds on these approaches by combining experiments (ITC) and MD simulations to study the physical chemistry of binding for a series of host–guest model systems selected for their rigidity and simplicity, in order to maximize the focus on the role of solvent in binding. We focus on two families of host molecules, cucurbit[*n*]urils (CB*n*) and cyclodextrins (CDs), examining a total of 16 host–guest pairs across a 50 K temperature range. Involving the temperature as a data dimension affords more information on solvation contributions, particularly for relatively rigid systems where the direct host–guest interactions are, to a first approximation, temperature-independent. The presented results provide new insight into the determinants of affinity and the role of solvent in binding.

## Results and discussion

### Experimental analysis

#### Host–guest systems

**Fig. 1:**
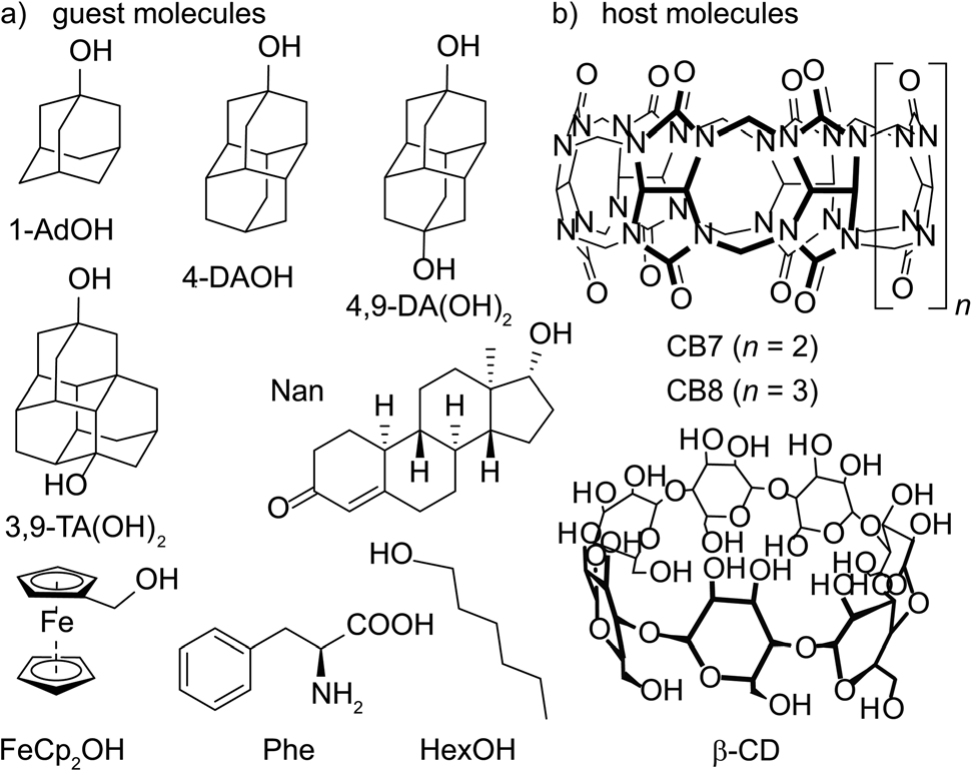
Chemical structures of water-soluble (a) guest molecules and (b) host molecules investigated in this study.

We used ITC to investigate the effects of temperature on supramolecular complexation in deionized water for seven CB7 complexes, six CB8 complexes, and three β-CD complexes (Fig. 1). The guests are all electrically neutral and most are close to rigid and fit snugly into the hosts' binding cavity. They include the hydroxylated ferrocene derivative FeCp_2_OH, as well as four hydroxylated diamondoids comprising one adamantane (1-AdOH), two diamantanes (4-DAOH, 4,9-DA(OH)_2_), and a triamantane (3,9-TA(OH)_2_).^79,83^ In addition, we studied three guest compounds that are more flexible, namely nandrolone (Nan), l-phenylalanine (l-Phe), and 1-hexanol (HexOH), in order to probe the robustness of the observed trends.

#### Binding thermodynamics at room temperature

The room temperature binding free energies, Δ*G*^298 K^_exp_, of the β-CD, CB7, and CB8 complexes span −5.5 to −14.2 kcal mol^−1^, with β-CD showing weaker binding than the other two hosts for a given guest, consistent with literature reports, and CB7 showing binding at least as strong as CB8 and sometimes stronger (Table 1). Particularly strong binding (Δ*G*^298 K^_exp_ < −10 kcal mol^−1^) is observed for CB7 with 1-AdOH and FeCp_2_OH, while CB7, as well as CB8, show similar binding free energies with the other guests tested here, except that the bulky guest 3,9-TA(OH)_2_ is too large to form CB7 inclusion complexes at all.

**Table 1:** Measured binding thermodynamics for all host–guest systems at 298 K. Numerical entries are measured binding enthalpy (Δ*H*^298 K^_exp_, kcal mol^−1^, SD ± 0.5 kcal mol^−1^), binding free energy (Δ*G*^298 K^_exp_, kcal mol^−1^, SD ± 0.5 kcal mol^−1^), binding entropy (−*T*Δ*S*^298 K^_exp_, kcal mol^−1^, SD ± 0.8 kcal mol^−1^), and change in heat capacity (Δ*C*_p,b_, cal mol^−1^ K^−1^, SD < 20%). Errors (standard deviation across replicates, SD) were determined by repeating the titrations at least three times. Based on our extensive experience with ITC studies over the years, we employed these error estimates as an upper bound. See ESI, Tables S1–S3 for the values (and https://zenodo.org for the values of each individual repetition) and Fig. S2 for reproducibility of the experiments and SD thereof. NB: no binding, *i.e.*, no inclusion complex formed. ND: not determined

Guest	CB7				CB8				β-CD			
	Δ*G*_exp_	Δ*H*_exp_	−*T*Δ*S*_exp_	Δ*C*_p,b_	Δ*G*_exp_	Δ*H*_exp_	−*T*Δ*S*_exp_	Δ*C*_p,b_	Δ*G*_exp_	Δ*H*_exp_	−*T*Δ*S*_exp_	Δ*C*_p,b_
1-AdOH	−14.2	−19.4	5.2	−102	−9.3	−8.1	−1.2	−83	−6.5	−6.5	0.0	−95
4-DAOH	−9.5	−12.1	2.6	−66	−9.1	−8.0	−1.1	−79	ND	ND	ND	ND
4,9-DA(OH)_2_	−9.6	−12.6	3.0	−135	−9.9	−7.7	−2.2	−103	−6.9	−8.9	2.0	−61
3,9-TA(OH)_2_	NB	NB	NB	NB	−9.5	−12.7	3.2	−97	ND	ND	ND	ND
FeCp_2_OH	−12.8	−21.0	8.2	−64	−9.0	−13.1	4.1	−55	−5.5	−7.7	2.2	−61
Nan	−8.9	−12.7	3.8	−144	−9.5	−8.9	−0.6	−105	ND	ND	ND	ND
l-Phe	−8.2	−9.5	1.3	−64	ND	ND	ND	ND	ND	ND	ND	ND
HexOH	−8.0	−9.6	1.6	−89	ND	ND	ND	ND	ND	ND	ND	ND

**Fig. 2:**
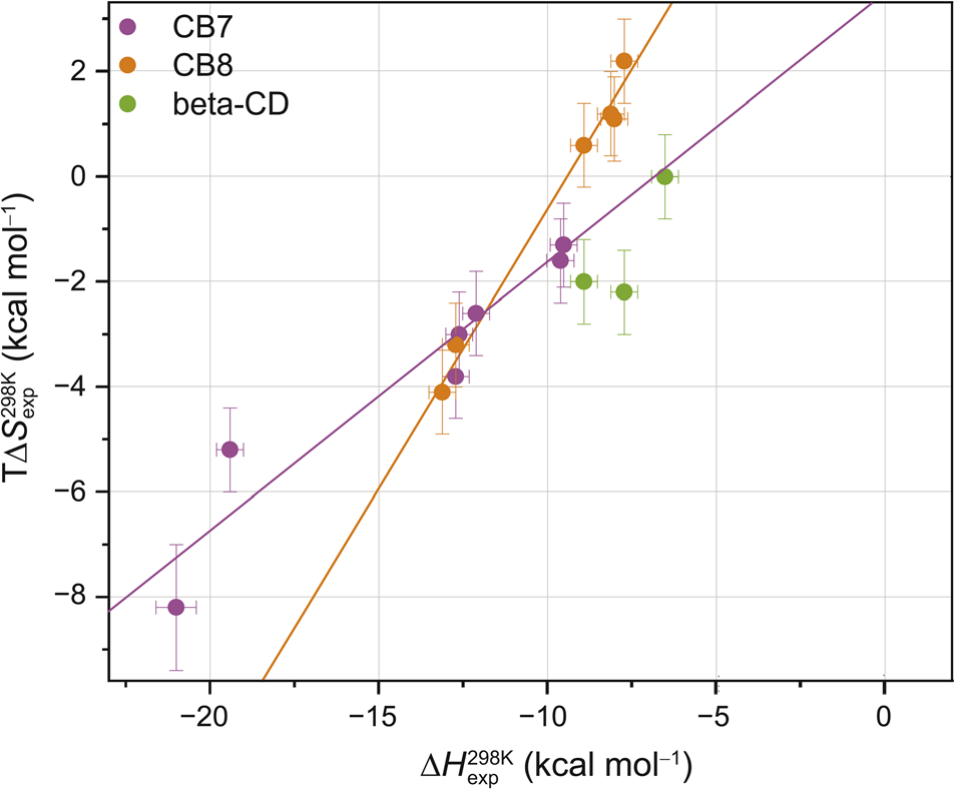
Enthalpy–entropy scatter plot for all investigated host–guest systems at 298 K. The straight lines correspond to linear least-square fits (CB7: slope = 0.50 ± 0.07, *R*^2^ = 0.91; CB8: slope = 1.05 ± 0.06, *R*^2^ = 0.99). Error bars show SD ± 0.5 kcal mol^−1^ for Δ*H*^298 K^_exp_ and SD ± 0.8 kcal mol^−1^ for *T*Δ*S*^298 K^_exp_. Typical errors (standard deviation across replicates, SD), determined by repeating the titrations at least three times, were smaller than 0.5 kcal mol^−1^ in Δ*H*_exp_ and 0.8 kcal mol^−1^ in −*T*Δ*S*_exp_. Based on our extensive experience with ITC studies over the years, we employed these error estimates as an upper bound. See ESI, Tables S1–S3† for the values (and https://zenodo.org for the values of each individual repetition) and Fig. S2† for reproducibility of the repetition experiments and SD thereof.

Interestingly, the enthalpic contributions to binding are uniformly favorable at 298 K (Fig. S1,† and Table 1). Strong enthalpic contributions to complex formation (Δ*H*^298 K^_exp_ < −12 kcal mol^−1^) were found for the CB7·1-AdOH, CB7·4-DAOH, CB7·4,9-DA(OH)_2_, CB7·FeCp_2_OH, CB7·Nan as well as CB8·3,9-TA(OH)_2_ and CB8·FeCp_2_OH complexes. Particularly large enthalpic contributions of −19.4 kcal mol^−1^ and −21.0 kcal mol^−1^ were observed for CB7·1-AdOH and CB7·FeCp_2_OH, respectively, consistent with literature reports.^84^ The entropic contributions to binding are relatively small (−*T*Δ*S*^298 K^_exp_ = −2.2 to 8.2 kcal mol^−1^) and, in most cases, unfavorable (Table 1, Fig. 2, and S1†). However, for CB8, a slightly favorable entropic contribution to binding is observed for several guests at 298 K. In contrast, for CB8·3,9-TA(OH)_2_ and CB8·FeCp_2_OH, modestly unfavorable binding entropy contributions were found.

These room temperature thermodynamic parameters indicate that counteracting effects are at play. For instance, while the binding free energy of 3,9-TA(OH)_2_ with CB8 is comparable to those of 1-AdOH, 4-DAOH, and 4,9-DA(OH)_2_, its enthalpic and entropic contributions are very different (Fig. S1†). As previously reported for host–guest^68,84–87^ and protein–ligand systems,^88–91^ we also see clear enthalpy–entropy compensation across systems at 298 K (*R*^2^(CB7) = 0.91 and *R*^2^(CB8) = 0.99, Fig. 2 and Table 2).

**Table 2:** Summary of calculated (GAFF 2.1) and experimental binding thermodynamics for the complexation of 1-AdOH and 4,9-DA(OH)_2_ with CB7, CB8, and β-CD at 298 K. The binding entropy −*T*Δ*S*_b_ is calculated by taking the difference between Δ*G*_b_ and Δ*H*_b_. All energy values are reported in kcal mol^−1^

Host·guest complex	Δ*G*_b_		Δ*H*_b_		−*T*Δ*S*_b_	
	exp	calc	exp	calc	exp	calc
CB7·1-AdOH	−14.2	−23.1	−19.4	−25.9	5.2	2.8
CB7·4,9-DA(OH)_2_	−9.6	−14.6	−12.6	−17.5	3.0	2.9
CB8·1-AdOH	−9.3	−15.2	−8.1	−10.0	−1.2	−5.2
CB8·4,9-DA(OH)_2_	−9.9	−19.4	−7.7	−15.1	−2.2	−4.3
β-CD·1-AdOH	−6.5	−8.4	−6.5	−7.8	0.0	−0.6
β-CD·4,9-DA(OH)_2_	−6.9	−9.8	−8.9	−11.2	2.0	1.4

#### Temperature dependency of binding thermodynamics

**Fig. 3:**
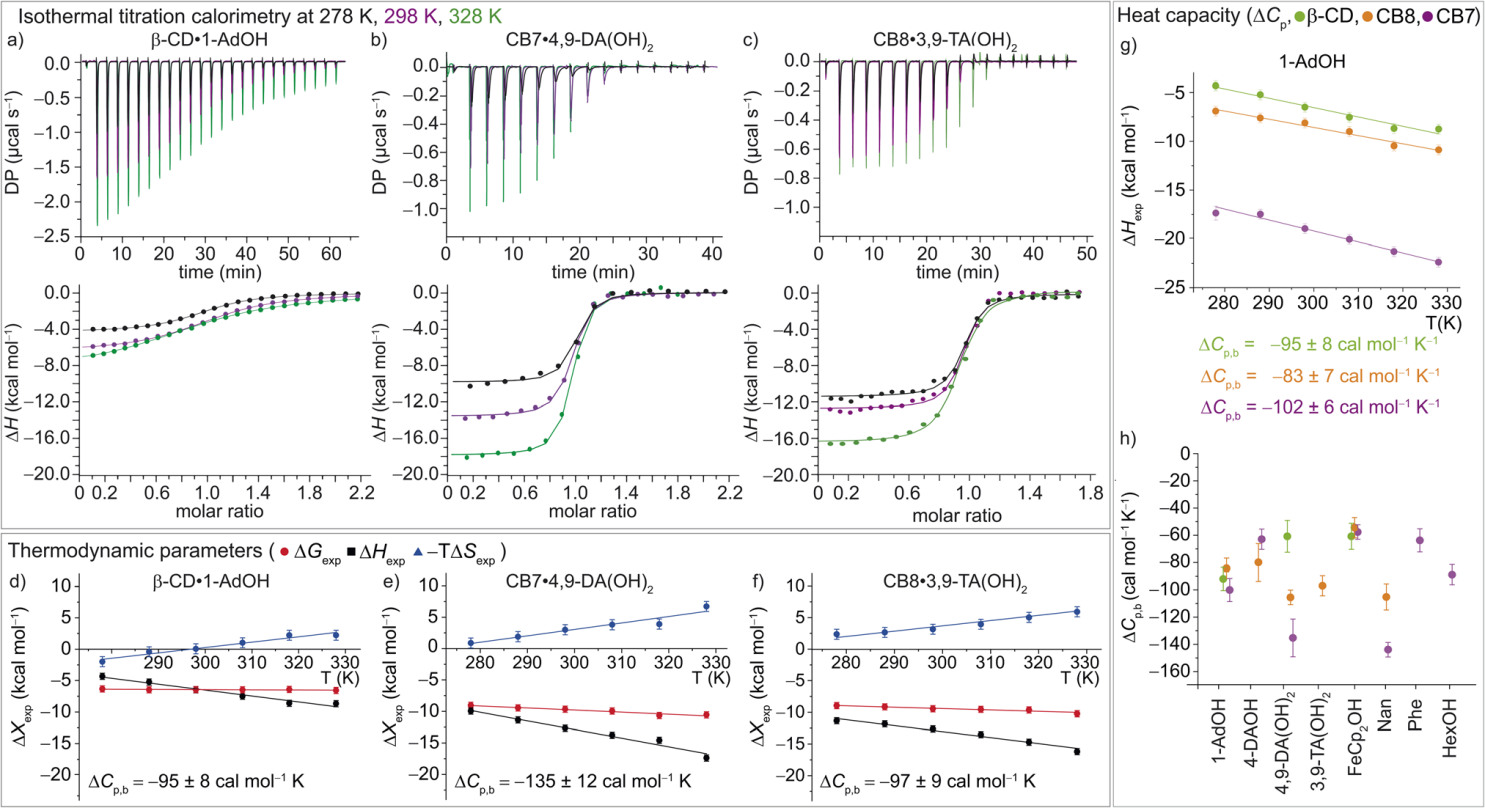
(a)–(c) ITC raw data and integrated heat plots for the complex formation of (a) β-CD·1-AdOH, (b) CB7·4,9-DA(OH)_2_, and (c) CB8·3,9-TA(OH)_2_ at 278 K (black), 298 K (violet), and 328 K (green). (d)–(f) Temperature dependence of the standard complexation parameters Δ*G*_exp_ (red), Δ*H*_exp_ (black), and −*T*Δ*S*_exp_ (blue) for (d) β-CD·1-AdOH, (e) CB7·4,9-DA(OH)_2_, and (f) CB8·3,9-TA(OH)_2_, from 278 to 328 K. Additionally, heat capacity changes (equal to the slope of the black Δ*H*_exp_ data points) are given with individual errors. (g) Measured binding enthalpies as function of temperature for 1-AdOH with β-CD (green), with CB8 (orange), and with CB7 (violet). (h) Changes of heat capacity (Δ*C*_p,b_) for all 16 host–guest complexes. Shown errors are the individual error values of the linear least-square fits. See ESI, Tables S1–S3† for details.

We used ITC to study all 16 complexes over a temperature range of 278 K to 328 K in 10 K steps. Representative enthalpograms for β-CD·1-AdOH, CB7·4,9-DA(OH)_2_, and CB8·3,9-TA(OH)_2_, each at 278, 298, and 328 K are shown in Fig. 3a–c, and the full set of enthalpograms is available in Fig. S3–S19.† The results are detailed in Tables S1–S3.† For all 16 host–guest systems, the Gibbs free energy Δ*G*_exp_ is almost unaffected by the temperature change (Fig. 3d–f and S23–S25†), though, due to the relationship *K*_a_ = exp(−Δ*G*_b_/*RT*), the affinity, defined by *K*_a_, does decrease with rising temperature (Tables S1–S4 and Fig. S20–S22†). For all host–guest systems studied here, the measured binding enthalpy was found to vary almost linearly with temperature (Fig. 3g, S23–S25†). Accordingly, a linear model was fitted to the data for each system to estimate its nearly temperature-independent change in heat capacity on binding, Δ*C*_p,b_, as the first derivative of the binding enthalpy. In all cases, the change in heat capacity on binding is negative (Fig. 3g, S26 and S27, and Tables S1–S3†), with values in the range of −55 to −144 cal mol^−1^ K^−1^.

In accordance with the common observation of enthalpy–entropy compensation,^92,93^ large, compensating changes in the enthalpic and entropic contribution to the binding free energy were observed for each host–guest system across the investigated temperature range (Fig. 3d–f and S23–S25†). In particular, the value of Δ*H*_exp_ becomes, on average, ∼0.75 kcal mol^−1^ more exothermic for each 10 K step, while for CB7·1-AdOH and CB8·Nan, each 10 K temperature increase caused an even larger increment of ∼1 kcal mol^−1^. As the free energy of binding was approximately constant with respect to temperature changes for these systems, there were compensating large changes in −*T*Δ*S*_exp_ with each change in temperature.

### Simulation analysis

We used molecular dynamics (MD) simulations, computational calorimetry^94^ based on MD, and grid inhomogeneous solvation theory (GIST)^95^ to gain insight into the binding thermodynamics observed experimentally. The computational analysis focuses on the six host–guest systems formed by CB7, CB8, and β-CD and the two diamondoid guests that were studied experimentally with all three of the hosts, *i.e.*, 1-AdOH and 4,9-DA(OH)_2_.

#### Comparison of calculation with experiment

**Fig. 4:**
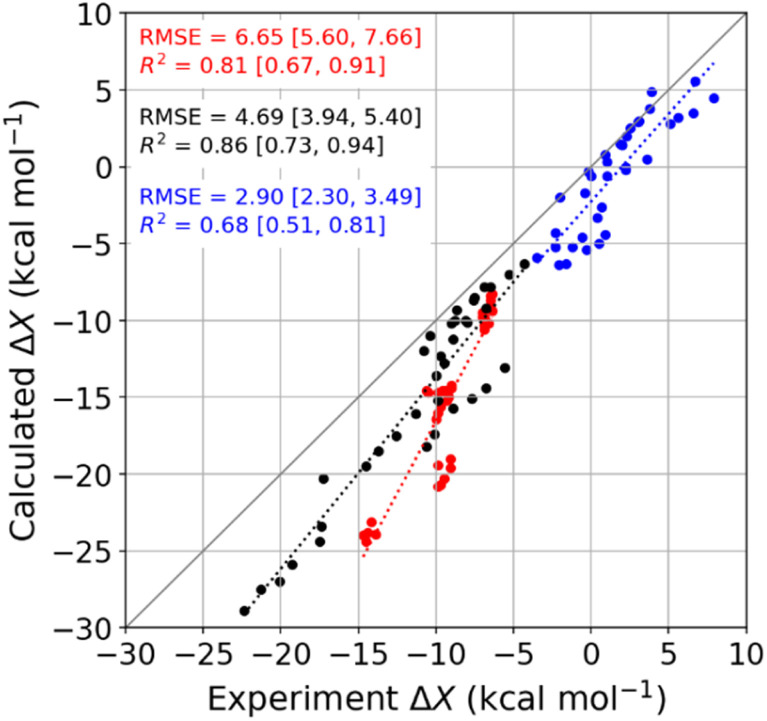
Comparison of MD-calculated thermodynamic quantities *versus* experimentally obtained values across the temperature range of 278 to 328 K. The thermodynamic quantities Δ*G*_b_, Δ*H*_b_, and −*T*Δ*S*_b_ are colored red, black, and blue, respectively. There are 36 data points for each thermodynamic quantity, and the error bars are omitted for clarity. See Table S9† for the corresponding numerical values and uncertainties. The root-mean-squared error (RMSE) and *R*^2^ values for each thermodynamic quantity are shown on the plot, and the values in square brackets give the 95% confidence interval obtained from bootstrapping.

The computed binding thermodynamics reproduce the experimental trends across the temperature range studied well (Fig. 4, S29, and Table S7†). Specifically, the *R*^2^ values of calculation *versus* experiment are close to 0.9 for Δ*G*_b_, 0.8 for Δ*H*_b_, and 0.7 for −*T*Δ*S*_b_. In addition, the slope of the computed binding enthalpy *versus* temperature is similar to the experimental slope, so the computed changes in heat capacity Δ*C*_p,b_ are similar to the experimental values. Thus, the simulations give a mean signed error for Δ*C*_p,b_, relative to experiment, of 5.8 cal mol^−1^ K^−1^ and RMSE of 18.3 cal mol^−1^ K^−1^, with a maximal relative error of only 20% (Table S11†). This concordance supports our use of the simulations to look more deeply into the molecular determinants of the binding thermodynamics. At the same time, we note that the computed thermodynamic quantities systematically overestimate the corresponding experimental data, with root-mean-squared errors (RMSE) in the range of 3–6 kcal mol^−1^ (Fig. 4).

Given that the calculations are based on the appropriate statistical thermodynamic theory, that there is no ambiguity in the protonation states of the hosts or guests, and that these systems are straightforward to converge numerically, due to the simplicity of the molecules, these errors presumably trace to problems with the force field, and in particular to an imbalance between the interactions of the free molecules with solvent and the interactions of the bound molecules with each other. Such results support the value of using host–guest binding data to evaluate (see Introduction) and even train simulation force fields.^96,97^

#### Conformations and fluctuations of bound complexes

**Fig. 5:**
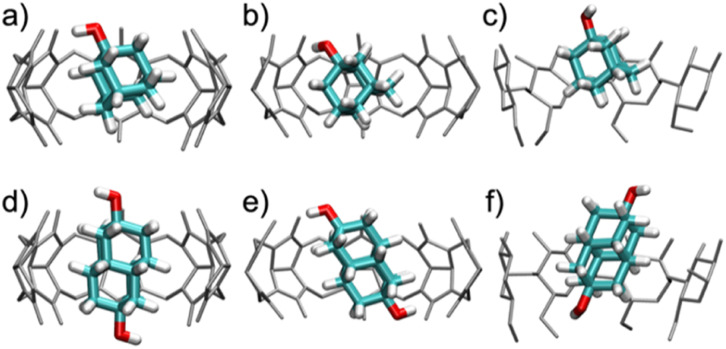
Snapshots of the characteristic binding geometries of (a) CB7·1-AdOH, (b) CB8·1-AdOH, (c) β-CD·1-AdOH, (d) CB7·4,9-DA(OH)_2_, (e) CB8·4,9-DA(OH)_2_, and (f) β-CD·4,9-DA(OH)_2_ observed in MD simulations at room temperature (298 K). Water molecules have been removed for clarity.

In order to provide a physical sense of the nature of the bound complexes, we characterized the conformations and fluctuations of the host–guest complexes in unrestrained, room temperature, 1 μs duration MD simulations. Fig. 5 provides representative snapshots of the predominant binding mode for each complex, while Fig. S31† reports the amount of conformational variation by plotting the time course of each guest molecule's axis of symmetry (*z*-axis) relative to the axis of symmetry of each host. We observed relatively tight conformational distributions for both the host and guest molecules in all six host–guest systems. In all cases, the hydrophobic core of the guest molecule sits firmly within the host cavity, and the guest's polar hydroxyl groups point to the portals of the hosts' cavity, where they may form hydrogen bonds with the polar groups fringing the portals, especially the carbonyls in CB*n*, and with the surrounding water. Although the guests rotate quite freely around the *z*-axis (data not shown), their angles relative to the *z*-axis are almost stable, as detailed below.

When bound within CB7, the smaller guest, 1-AdOH, remains tilted by about 25° from the *z*-axis during the entire 1 μs simulation, with fluctuations of only about 10° (Fig. S31b†). This angle enables it to donate a hydrogen bond to a carbonyl oxygen at a single portal (Fig. 5a). In CB8, 1-AdOH again hydrogen bonds to the portal carbonyls (Fig. 5b), but the hydroxyl end of the guest flips rapidly between portals, donating first to one portal and then to the other (Fig. S31c†), as permitted by the larger cavity of CB8. In β-CD, 1-AdOH usually sits with its hydroxyl at the secondary face but occasionally flips briefly in the opposite orientation (Fig. S31d†). However, it does not typically form hydrogen bonds with the host's hydroxyl groups (Fig. 5c). When bound within CB7, the larger guest, 4,9-DA(OH)_2_, remains highly aligned with the *z*-axis (angled by only about 5°) and forms no hydrogen bonds with the host CB7 (Fig. 5d and S31f†). Apparently, the tight fit of this elongated guest inside the CB7 cavity prevents it from tilting enough to form hydrogen bonds with the host carbonyls. In contrast, the larger cavity of CB8 allows 4,9-DA(OH)_2_ to tilt by about 35° from the *z*-axis (Fig. 5e and S31g†) so that each of its hydroxyl groups donates a hydrogen bond to a carbonyl at its respective portal. These hydrogen bonds in CB8·4,9-DA(OH)_2_ break occasionally, but we did not observe the molecule flipping around the *z*-axis, presumably due to the lack of space in the cavity. In β-CD, 4,9-DA(OH)_2_ behaves much as in CB7: it remains rather well aligned with the *z*-axis, does not flip, and does not form hydrogen bonds with the host (Fig. 5f and S31h†). Again, this is likely due to the bulky and elongated shape of the guest.

#### The role of hydrogen bonds

We explored whether the formation of hydrogen bonds might correlate with binding affinity. A hydrogen bond was considered to exist when the donor–acceptor distance was less than 3.0 Å, and the donor-hydrogen-acceptor angle was greater than 150° (see also ESI† for further information).

**Fig. 6:**
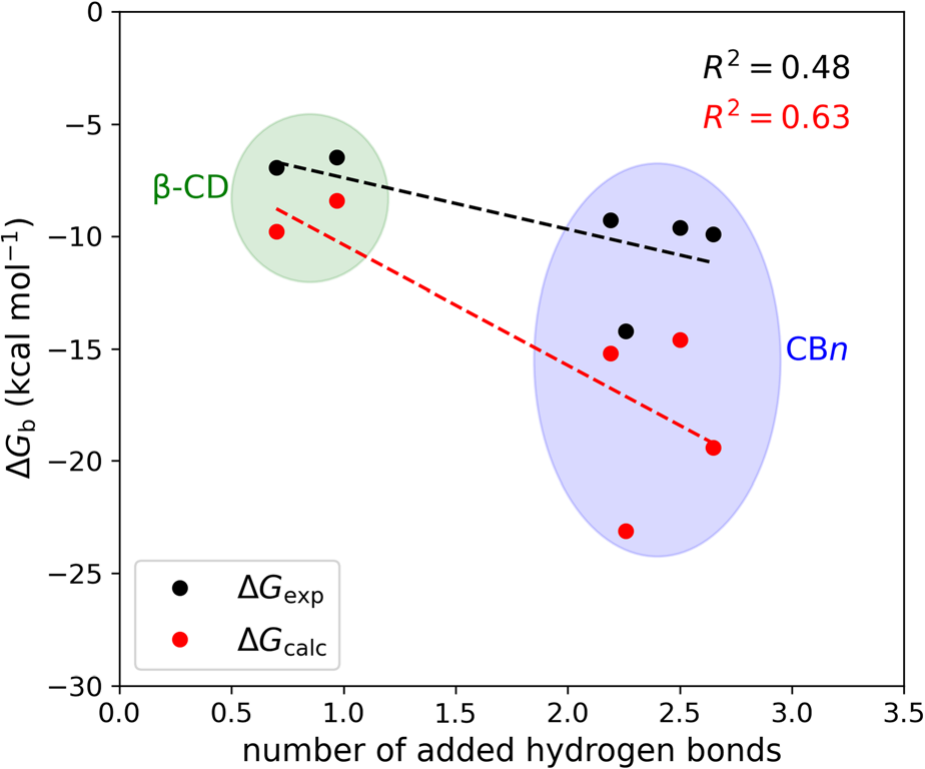
Binding free energy *versus* the number of added hydrogen bonds upon binding at 298 K. The number of added hydrogen bonds includes the change in host–guest, host–water, guest–water, and water–water hydrogen bonds upon binding.

The mean number of hydrogen bonds between the host and guest in the simulations does not correlate strongly with binding free energy: *R*^2^ is 0.53 for the computed free energies and only 0.25 for the experimentally measured free energies (graph not shown). However, better correlations are obtained when we account for all possible hydrogen bonds, *i.e.*, host–guest, guest–water, host–water, and water–water hydrogen bonds (Fig. 6). The coefficients of determination (*R*^2^) with all hydrogen bonds included are 0.63 for the computed binding free energies and 0.48 for the measured binding free energies. We note that if we exclude the outlier, *i.e.*, the complex CB7·1-AdOH, from the calculated and experimental data, the *R*^2^ increase to 0.85 and 0.96, respectively.

It is also worth noting that these correlations derive entirely from the differences between the β-CD results and the CB*n* results. The breakdown of the hydrogen bonds for all six host–guest complexes is summarized in Table 3. For instance, we observed one hydrogen bond formed between the host and guest upon binding for CB7·1-AdOH. This is accompanied by a reduction of one hydrogen bond each between water and the host and guest molecules. The loss of hydrogen bonds with the solutes is balanced by an increase of three hydrogen bonds to bulk water. Thus, the complex formation of CB7·1-AdOH produces a net gain of about two hydrogen bonds. At a coarse level, the more hydrogen bonds gained on binding over the entire solute–water system, the stronger the binding affinity. The linear regression of binding free energy against the number of hydrogen bonds formed (Fig. 6) yields −5.4 kcal mol^−1^ per hydrogen bond for the computed binding free energy and −2.6 kcal mol^−1^ per hydrogen bond for the experimental binding free energy. These are reasonable magnitudes for the strength of a hydrogen bond.^6^ It is noteworthy that amidst the myriad of other factors contributing to binding free energy, such as dispersion interactions and entropy, the hydrogen bond count has emerged as a clear correlate of affinity. Our findings resonate with the generalized hydrogen bonding concept for intermolecular recognition, as initially expounded by Hunter.^65,98^ As shown in Fig. 6 and Table 3, the total number of hydrogen bonds increases by ∼1 when the guests bind to β-CD and 2-3 for CB*n*. The smaller increases for β-CD trace chiefly to smaller increases in the number of water–water hydrogen bonds (Table 3).^99^ This may result from the fact that the free CB*n* contains collections of water molecules that lack a bulk-like complement of hydrogen bonds with other water molecules. In addition, the CB*n* cavity water molecules are only weakly hydrogen-bonded to the host. This explanation is qualitatively consistent with the concept that water molecules inside the free CB*n* are at a higher energy level (fewer hydrogen bonds) than bulk water.^7,75,77^

**Table 3:** Change in the mean number of hydrogen bonds and the mean number of waters expelled from the cavity, Δ*N*_wat_, in simulations upon binding at room temperature (298 K). The total number of hydrogen bonds is equal to the sum of host–guest (H–G), host–water (H–W), guest–water (G–W), and water–water (W–W)

Host·guest complex	H–G	H–W	G–W	W–W	Total	Δ*N*_wat_
CB7·1-AdOH	0.6	−0.7	−0.8	3.1	2.2	6.4
CB7·4,9-DA(OH)_2_	0.0	0.0	−0.1	2.6	2.5	6.4
CB8·1-AdOH	0.2	−0.2	−0.5	2.7	2.2	9.2^a^
CB8·4,9-DA(OH)_2_	1.2	−1.2	−1.5	4.2	2.7	10.3^a^
β-CD·1-AdOH	0.1	−0.4	−0.5	1.8	1.0	7.2
β-CD·4,9-DA(OH)_2_	0.0	−1.0	−0.2	1.8	0.6	7.7

a The guests expel more water from CB8 than CB7 due to the larger number of water molecules initially present in the CB8 cavity. Both the CB8 and CB7 cavities are essentially water-free following binding.

#### Molecular and force field decompositions of the binding enthalpy

For a liquid state system at 1 atm pressure, the change in volume on binding is tiny, and the pressure–volume contribution to the binding enthalpy is negligibly small. Therefore, binding enthalpy is simply the change in mean potential energy on binding. Since the force field used here is pairwise additive, it becomes straightforward to decompose the binding enthalpy into informative terms. We consider two such decompositions.

One decomposition breaks down the binding enthalpy into two components that add to the total: Δ*H*_calc_ = Δ*H*_H–G_ + Δ*H*_desolv_. The first component, Δ*H*_H–G_, includes all energy terms that involve only the host and guest, namely their internal energies in the bound or complexed state and their interaction energy in the bound state. The second term, Δ*H*_desolv_, includes all energy terms that involve water molecules, namely all host–water and guest–water interactions in the bound or complexed state and all water–water interactions. As shown in Table S10,† the binding enthalpies are strongly favored by the host–guest term, especially for the complexes where shape and size match are expected to be particularly favorable, while the desolvation term opposes binding, particularly for the larger 4,9-DA(OH)_2_ guest. It is instructive to compare the case of CB7 and 1-AdOH with that of β-CD and the same guest: CB7 achieves much more favorable binding enthalpy chiefly by providing more favorable host–guest energies, along with similar or less unfavorable desolvation energies. From this perspective, the binding of CB7 is more exothermic because of a similar or smaller desolvation penalty along with greater gains in solute energy. Note that the latter could result from favorable host–guest interactions and reorganization enthalpies that are small in magnitude.

The second decomposition breaks down the binding enthalpy into the various force field terms, notably the Lennard-Jones (LJ) treatment of van-der-Waals interactions, electrostatic interactions among the partial charges of the atoms, and so-called bonded or valence terms comprising bond stretches, angle-bends, and torsions. Decomposition of Δ*H*_calc_ at room temperature along these lines (Table S8†) shows that the LJ and electrostatic terms are favorable for all host–guest pairs, while the valence term tends to be smaller in magnitude and is usually unfavorable. The particularly favorable LJ interactions obtained in the case of CB7·1-AdOH presumably result from the snug fit of their bound complex. Given that hydrogen bonding is represented in General Amber Force Field (GAFF) primarily through the electrostatic term and that we observed a good correlation between the number of added hydrogen bonds and the binding free energy, it makes sense that the calculated binding free energy and the electrostatic part of the binding enthalpy correlate with *R*^2^ = 0.73. The computed binding free energy also correlates with the LJ part of the enthalpy, but not as well (*R*^2^ = 0.43).

#### Solvent's role in the temperature dependence of binding thermodynamics

For all six host–guest systems studied computationally, the computed binding enthalpy Δ*H*_calc_ at 328 K is 3.9 to 5.5 kcal mol^−1^ more favorable (more negative) than at 278 K (Table S10†). However, Δ*H*_H–G_, which comprises the changes in intra- and intermolecular host–guest interactions on binding, is nearly independent of temperature for all six systems, rising by merely 0.03 to 0.6 kcal mol^−1^ as the temperature goes from 278 to 328 K. The insensitivity of Δ*H*_H–G_ to temperature presumably traces to the relative rigidity of the hosts and guests studied here and the constrained conformations of their complexes discussed above. That is, raising the temperature has little effect on the conformational distributions of the free and bound forms, so the energy terms associated with the host and guest and their mutual interactions are not affected much.

It follows from the above that virtually all of the temperature dependency of the binding enthalpy comes from Δ*H*_desolv_, as confirmed by the data in Table S10.† Intuitively, Δ*H*_desolv_ reports the energy cost of partly dehydrating (stripping water molecules from) the solutes when they bind. The present data thus mean that the enthalpic cost of stripping waters from the solutes on binding falls with increasing temperature. This makes sense because warmer water is less free to fall into low-energy configurations around the solutes, therefore, stripping them off upon binding is less energetically costly. Thus, warmer water leads to more enthalpically favorable binding if the host and guest energy contributions are largely temperature independent.

The observed more exothermic binding at higher temperatures explains why the changes in heat capacity on binding all are negative in sign (see heat capacity decomposition in Table S12†). Note that the same physical reasoning applies to the hydration of both polar and non-polar solutes. However, changes in the solutes' conformational distributions with temperature could lead to more complicated and less predictable outcomes for more flexible solutes and complexes.

We also observed that the computed entropy changes on binding are consistently less favorable with increasing temperature (Tables S9 and S10†). Although we did not attempt an entropy decomposition analogous to the enthalpy decomposition used above, the relative rigidity of the solutes and their complexes suggests that most of the temperature dependence of the binding entropy, similarly, derives from changes in the conformational distribution of water. This suggests, in turn, that raising the temperature leads to a greater increase in the entropy of water at the surfaces of the free host and guest than in bulk. As a consequence, release of the surface water on binding at high temperature becomes less entropically favorable (or more unfavorable) than release at low temperature. The resulting changes in −*T*Δ*S*_calc_ with temperature cancel, or compensate, roughly 70% of the corresponding changes in Δ*H*_calc_, leading to modest increases in binding free energy (Δ*G*_calc_ more negative) with rising temperature.

We used grid inhomogeneous solvation theory (GIST) to spatially map the solvent's contributions to the computed changes in heat capacity Δ*C*_p,b_ (Tables S11, S13, and Fig. S32†). We are not aware that such a spatial decomposition of Δ*C*_p,b_ has been attempted previously. As shown in Table S11,† the GIST-calculated Δ*C*_p,b_ agrees reasonably well with both the experiments and MD simulations. The GIST results do not include the solute–solute interactions, but this is a good approximation because Δ*H*_H–G_ is essentially independent of temperature (Table S10†). The contours in Fig. S32† visually show that Δ*C*_p,b_ is negative in the cavity region for all host–guest complexes, as water is expelled from this region upon binding. The portal region, however, shows negative and positive contours, which are attributable to solvent reorganization upon binding the guest molecule. The contours also show more differences in the portal region for CB*n* than for β-CD. To probe this further, we integrated the GIST voxels over two regions, inside and outside the cavity, Δ*C*_p,cavity_ and Δ*C*_p,portal_, respectively, and list them in Table S13.† For all six host–guest complexes, Δ*C*_p,cavity_ contributes more than Δ*C*_p,portal_ to the total value. One also notices that Δ*C*_p,cavity_ is larger (*i.e.*, more negative) in β-CD than in CB*n*, but the opposite is true for Δ*C*_p,portal_. The quantity Δ*C*_p,portal_ contributes very little to the total Δ*C*_p,b_ in β-CD but is significant for CB*n*. The GIST decomposition thus suggests that solvent reorganization around the portal region of CB*n*, with their highly polar carbonyl oxygens, plays an important role in binding. In β-CD, the water molecules near the portals behave more bulk-like due to weaker interactions with the hydroxyls of CDs compared to the carbonyl oxygens in CB*n*.

### Interpretations

#### The high affinity of CB*n*-guest binding

Various explanations have been introduced to rationalize the high affinities achieved by CB*n*. For instance, it is well accepted that cationic groups can form energetically favorable interactions with the electronegative carbonyl oxygens at the portals of these hosts, and apolar groups can form stabilizing London dispersion interactions with the interior of the binding cavity.^50,74,100^ In addition, because CB*n* has particularly rigid, concave, non-polar, and well-enclosed binding cavities, the water they contain may be energetically unstable^75–78^ compared with the water molecules in other hosts or the bulk, making displacement of water from the cavity relatively easy and leading to more favorable guest binding than typically observed for other hosts. (It may seem counterintuitive that water in a binding cavity is “unstable”; after all, if it is unstable, why is it present? In brief, if the water were to exit the cavity, there would be an even more unstable water–vacuum interface at the portals of the cavity, leading to a higher overall free energy of the system. Note, too, that the familiar concept of surface tension, which says that the free energy of a water droplet rises linearly with its surface area, immediately implies the existence of “high free energy” water at the surface.) It has also been argued that the rigidity of CB*n* and their tightest-binding guests is the reason for the particularly small losses in the configurational entropy associated with both solutes.^101^

The present experimental data show that the high affinities of CB*n* are attributable to highly favorable binding enthalpies along with, for the most part, only weakly unfavorable binding entropies. The computational results are in broad accordance with these observations. The binding entropies of the cyclodextrins are generally similar to those of CB*n*, and the weaker binding of cyclodextrins is attributable to their less favorable binding enthalpies. It is thus of particular interest to consider what may account for the high binding enthalpies of CB*n*. Indeed, it has been a long-standing riddle why CB*n* complexes, especially CB7 complexes, show much larger exothermic binding signatures than the corresponding cyclodextrin complexes, despite similar cavity dimensions.^101–103^ In 2012, we proposed that the release of energetically frustrated water from the binding cavities of the CB*n* upon binding might account for the outstanding affinities of these hosts.^29,45^ In particular, water molecules sequestered in a non-polar CB*n* cavity form fewer hydrogen bonds than water molecules in bulk. The release of these waters upon guest binding restores their hydrogen-bonding potential.^77^ According to the explanation model, the cavity water molecules inside the cyclodextrin cavities retain more interactions with the surrounding water and are partially hydrogen-bonded to the host, so they are more stable and gain fewer hydrogen bonds upon release. This view is at least partly consistent with observations made in the present study, *e.g.*, the enthalpy gain upon host–guest complex formation that is accompanied by the release of cavity water molecules. Thus, we see larger increases in the total hydrogen bond count for CB*n* systems than for β-CD upon binding, and this difference is only fully apparent when water hydrogen bonds are accounted for. In addition, the changes in water–water interaction energy on binding are more favorable for CB7 than for β-CD with the same guests by about 5 kcal mol^−1^ (Table S10†). On the other hand, these advantages are largely balanced by other solvation components, such as a much greater loss of favorable guest–water interactions on binding to CB7 than to β-CD (Table S10†), so that the computed values of Δ*H*_desolv_ are not grossly different between CB7 and the similarly sized β-CD. The differences in computed binding enthalpy between these two hosts instead trace primarily to differences in direct host–guest interactions Δ*H*_H–G_ (Table S10†), presumably, because the diamondoid guests fit more snugly in CB7 than in β-CD and form a more extensive array of attractive interactions. In other words, a direct comparison of the room temperature binding free energies, enthalpies, and entropies between host–guest complexes of cyclodextrins and cucurbit[*n*]urils may not be well suited to investigate the hydrophobic contributions to binding due to the largely different binding geometry and host–guest contact.

#### Changes in heat capacity

**Fig. 7:**
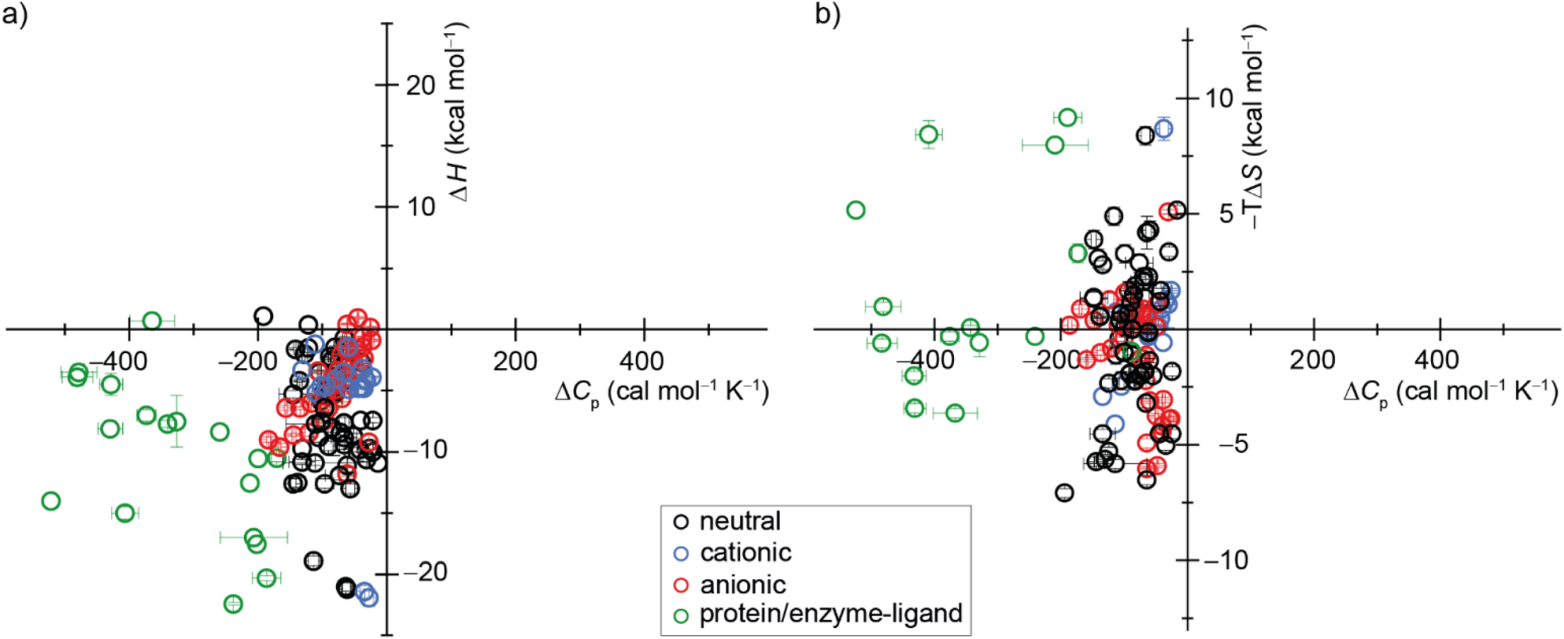
Display of a scatter plot between host–guest and protein–ligand (a) binding enthalpies (Δ*H*_exp_) and (b) binding entropies (−*T*Δ*S*_exp_) *versus* heat capacity changes (Δ*C*_p,b_). See ESI, Tables S5 and S6 as well as Fig. S28† for details on the host–guest pairs, experimental conditions, exact values, and references.

For biomacromolecules, many effects can influence Δ*C*_p,b_, resulting in values ranging from near-zero to −1500 cal mol^−1^ K^−1^ (Fig. 7 and Table S5†). Furthermore, heat capacity changes akin to those observed in biomolecular processes are an inherent consequence in any system displaying cooperative transitions involving diverse weak interactions, irrespective of the interaction nature.^104^ Consequently, deriving general insight into heat capacity changes on binding from protein–ligand interaction studies is challenging. Host molecules with relatively rigid guest molecules have distinct advantages as model systems because their simplicity eliminates many potentially complicating effects. All cases of host–guest complexes investigated here, along with most previously reported systems (Fig. 7 and Table S6†) exhibit negative Δ*C*_p,b_ values. In addition, these systems have mostly negative binding enthalpies (−22.0 to +1.0 kcal mol^−1^), while their binding entropies spread across positive and negative values (−6.6 to +8.6 kcal mol^−1^). Given that the present systems are largely nonpolar, these results militate against traditional explanations of the hydrophobic effect that revolve around entropies (guided by Kauzmanns' original hypothesis^13^). Instead, heat capacity changes and enthalpies appear to be a more unifying theme for host–guest complexation. Indeed, large, negative heat capacity changes at constant pressure (Δ*C*_p,b_) are often considered as the most definitive evidence for the hydrophobic effect, which arises due to the structuring of water molecules surrounding non-polar moieties.^105–108^ These water molecules possess a higher heat capacity change and a lower entropy than bulk water. Consequently, classical hydrophobic interactions, in which non-polar surfaces are shielded from bulk water, are typically characterized by a favorable entropic binding signature and a negative change in heat capacity.^106,109,110^ Diederich and colleagues proposed an alternative model to account for the negative values of heat capacity changes observed during the complexation of dipolar benzene derivatives and cyclophanes. In their model for the non-classical hydrophobic effect, “changes in the strong interactions between these guests and their solvent cage dominate the changes in complexation enthalpies that occur by altering the temperature.”^68^

Our study offers a complementary perspective. We observe that increasing temperature consistently renders binding more enthalpically favorable, both experimentally and computationally, signifying a negative change in heat capacity upon binding. Moreover, virtually the entire change in heat capacity traces directly to the solvent, as indicated by the enthalpic decompositions (Tables S10 and S12†) and rationalized by the relative rigidity of these host–guest systems. We deduce that water becomes a less favorable solvent as the temperature rises, thereby imposing a diminished enthalpic penalty for binding. In addition, our GIST results indicate that the solute–water interface in the portal region of the macrocyclic host plays a more important role in binding for CB*n* than for β-CD, and the binding process does not depend solely on the desolvation of the cavity by the bound guest molecule. Naturally, discerning this pattern in studies involving more flexible host–guest systems and complex molecules, such as proteins, will be more challenging, given that alterations in the solute conformational distribution are expected to contribute substantially and perhaps unpredictably to the observed heat capacity changes upon binding.

## Conclusions

The mechanistic interpretation of observed heat capacity changes for molecular associations, *e.g.*, protein–ligand complexes, is a long-standing challenge. Due to the simplicity of the host–guest systems selected here, we were better able to dissect contributions from solvent effects and direct binding on Δ*C*_p,b_. Concretely, we experimentally and computationally investigated CB*n* and β-CD host–guest complexes with adamantane-, diamantane-, and triamantane-type guests in a temperature range from 278 to 328 K. Our findings revealed a substantial increase (up to 45%) in the enthalpic driving force for binding, corresponding to negative heat capacity changes (−55 to −144 cal mol^−1^ K^−1^). The favorable shifts in binding enthalpy with temperature were counterbalanced by compensatory changes in binding entropy, resulting in a nearly constant binding free energy as temperature increased. The computationally observed temperature independence of the direct host–guest interaction combined with GIST water map analysis enabled us to attribute the temperature dependencies to local solvation effects. We argue that as temperature rises, water becomes less favorable as a solvent from an enthalpic point of view, thereby reducing the enthalpic penalty associated with host and guest desolvation upon binding.

This perspective accounts for the consistently negative changes in heat capacity of binding for cucurbit[*n*]urils and cyclodextrins and is expected to play a general role in molecular associations in water.

## Data availability

All raw and processed data generated in this study, including data presented in the main manuscript and the ESI† file, have been deposited in the https://zenodo.org database (DOI: https://doi.org/10.5281/zenodo.7082003). Furthermore, binding parameters and chemical structures from this study are available on https://suprabank.org (DOI: https://doi.org/10.34804/supra.20220330425). In addition, the scripts used to prepare and analyze the computational studies are available at https://github.com/jeff231li/adamantanes-temperature-scripts.

## Author contributions

LMG: conceptualization, methods, validation, investigation, data curation, visualization, writing – original draft. JS: formal analysis, investigation, methods, software, validation, visualization, writing – review and editing. BT & PRS: isolation, functionalization, and characterization of diamondoid alcohols. MKG: conceptualization, funding acquisition, methodology, project administration, resources, supervision, writing – review and editing. FB: conceptualization, funding acquisition, methodology, project administration, resources, supervision, writing – original draft.

## Conflicts of interest

M. K. G. has an equity interest in and is a co-founder and scientific advisor of VeraChem LLC.

## Supplementary Material

SC-014-D3SC01975F-s001
