# Supplementary material for: The temperature-dependence of host–guest binding thermodynamics: experimental and simulation studies
Source: Chem Sci. 2023 Oct 13;14(42):11818–29. doi: 10.1039/d3sc01975f (PMC10619620; doi:10.1039/d3sc01975f)
Supplement: SC-014-D3SC01975F-s001 [file SC-014-D3SC01975F-s001.pdf]

Electronic Supplementary Material (ESI) for Chemical Science.
This journal is © The Royal Society of Chemistry 2023

Electronic Supplementary Information

for

The Temperature-Dependence of Host-Guest Binding Thermodynamics: Experimental and Simulation Studies

Laura M. Grimm,‡*[a] Jeffry Setiadi,‡[b] Boryslav Tkachenko,[c]
Peter R. Schreiner,[c] Michael K. Gilson,*[b] Frank Biedermann*[a]

[a] Institute of Nanotechnology (INT), Karlsruhe Institute of Technology (KIT), Hermann-von-Helmholtz Platz 1, 76344 Eggenstein-Leopoldshafen, Germany
E-Mail: laura.grimm@kit.edu, frank.biedermann@kit.edu

[b] Skaggs School of Pharmacy and Pharmaceutical Sciences, University of California, San Diego, 9255 Pharmacy Lane, La Jolla, CA 92093
E-Mail: jsetiadi@health.ucsd.edu, mgilson@health.ucsd.edu

[c] Institute of Organic Chemistry, Justus Liebig University Giessen, Heinrich-Buff-Ring 17, 35392 Giessen, Germany
E-Mail: peter.r.schreiner@org.chemie.uni-giessen.de

‡ These authors contributed equally.

Materials and Methods

Chemicals. All commercial chemicals were used without further purification. Cucurbit[*n*]urils (CB*n*) were purchased from Strem Chemicals or synthesized following literature reports.[1, 2] CB*n* samples were desalted with a biotech-grade dialysis membrane (cellulose ester/regenerated cellulose) system to remove impurities, such as water, hydrogen chloride, ammonium, and alkali metal ion salts, typically introduced in the course of their preparation and purification, which can significantly influence the binding parameters.[3] To avoid previously observed decomposition, all desalted CB*n* stock solutions used for fundamental binding studies in deionized water were prepared freshly every couple of days. Diamantane and triamantane hydroxy derivatives were synthesized following literature procedures.[4]

Sample Preparation. All stock solutions were prepared in Millipore H_2O and kept in the fridge at 277 K for storage. Concentrations of dye stock solutions were determined by UV-Vis absorption titration measurements (BC: ε_{344nm} = 22300 M^{-1} cm^{-1};[5] cobaltocene: ε_{261nm} = 34200 M^{-1} cm^{-1};[6] MDAP: ε_{393nm} = 7800 M^{-1} cm^{-1};[7] MPCP: ε_{335nm} = 7111 M^{-1} cm^{-1} [8]). Concentrations of CB7 and CB8 solutions were determined by ITC titration experiments with bis(cyclopentadienyl)cobalt(III) hexafluorophosphate as guest, and by independent emission-based IDAs with MDAP (CB7) and MPCP (CB8).[6, 8] The determined concentrations of the two different methods were in good agreement. The concentration of the β-CD solution was determined by ITC with 1-AdOH as guest, and the values were in accordance with all other here reported guests. The concentrations of the guest molecules were determined by ITC titrations with known host concentrations or, in the case of 1-AdOH, by IDA titration with CB7•MDAP.

Spectroscopy. Absorption spectra were measured on a Jasco V-730 double-beam UV-Vis spectrophotometer and baseline corrected. Steady-state emission spectra were recorded on a Jasco FP-8300 fluorescence spectrometer equipped with a 450 W xenon arc lamp and double-grating excitation and emission monochromators. Emission and excitation spectra were corrected for source intensity (lamp and grating) and the emission spectral response (detector and grating) by standard correction curves. Fluorescence-based titration curves were performed manually or by an ATS-827 automatic titration unit to obtain the K_a values (CB7•BC, CB7•MDAP, and CB7•1-AdOH). The temperature was varied between 278 and 328 K in 10 K steps by using a water thermostat cell holder (STR-812), while the cuvettes were equipped with a stirrer allowing rapid mixing. The acquired data was fitted following literature reports.[9] All binding affinity measurements were repeated at least three times for all systems studied, and the typical errors (standard deviation across replicates, SD) were determined to be less than 25% in K_a (0.2 in $\log K_a$).

Isothermal Titration Calorimetry. Isothermal titration calorimetry (ITC) experiments were carried out under air in Millipore H_2O on a Microcal PEAQ-ITC from Malvern in analogy to our previous report.[10] The temperature was adjusted before each run by the

internal temperature control system and then kept constant during the run (temperature range from 273 to 328 K in 10 K steps). All concentrations were kept constant during titrations except that of the titrant. In a typical experiment, 1.5 μL guest solution (the first injection was 0.4 μL) was injected 25 times into the ITC cell (spacing: 150 s; stir speed: 750 rpm; initial delay: 60 s; injection duration: 6 s), which contained the host at 10 times lower concentration. The data was analyzed by Microcal PEAQ-ITC analysis software with the one-set-of-sites model. The data was baseline corrected by the average value of the titration curve of the guest into water. The first data point from the 0.4 μL injection was always omitted.

Most binding constants were directly accessible by curve fitting of the isotherms and were converted to binding free energies via $\Delta G_{\mathrm{exp}} = -\mathrm{RT}\ln K_{\mathrm{a}}$. The K_{a} values for the ultrahigh-affinity complexes of CB7•$FeCp_2OH$ and CB7•1-AdOH had to be determined by competitive methods ($FeCp_2OH$: **Figure S6**, multistep ITC with phenylalanine as competitor, and 1-AdOH: **Figure S20**, fluorescence-based IDA with MDAP as competitor). Furthermore, ITC experiments give direct access to the binding enthalpy (ΔH_{exp}). The entropic contributions to binding ($-\mathrm{T}\Delta S_{\mathrm{exp}}$) were obtained from the directly measured ΔH_{exp} and ΔG_{exp} values through $-\mathrm{T}\Delta S_{\mathrm{exp}} = \Delta G_{\mathrm{exp}} - \Delta H_{\mathrm{exp}}$.

Error value estimation. Repetition experiments, including those with newly prepared stock solutions, were carried out to estimate the experimental error values (**Figure S2**). The resulting thermodynamic parameters, *i.e.*, K_{a}, ΔG_{exp}, ΔH_{exp}, and $-\mathrm{T}\Delta S_{\mathrm{exp}}$ are provided in **Tables S1-S3**. Typical errors (standard deviation across replicates, SD), determined by repeating the titrations at least three times, were smaller than 20% in K_{a}, 0.2 in $\log K_{\mathrm{a}}$, 0.5 kcal mol^{-1} in ΔH_{exp} and ΔG_{exp}, and 0.8 kcal mol^{-1} in $-\mathrm{T}\Delta S_{\mathrm{exp}}$. Based on our extensive experience with ITC studies over the years, we employed these error estimates as an upper bound. All raw and processed data generated in this study, including data presented in the main manuscript and the Supplementary Information file and errors, have been deposited in the zenodo.org database (DOI: 10.5281/zenodo.7082003).

Experimental Results

Table S1. Binding parameters for the complexation of guests with desalined CB7 in deionized water determined by ITC. The data was averaged from at least three dilution heat-corrected experiments. Typical errors (SD) are 20% in K_a, 0.2 in $\log K_a$, 0.5 kcal mol^{-1} in ΔH_{exp} and ΔG_{exp}, and 0.8 kcal mol^{-1} in $-T\Delta S_{exp}$. Errors (standard deviation across replicates, SD) were determined by repeating the titrations at least three times. Based on our extensive experience with ITC studies over the years, we employed these error estimates as an upper bound. Values for each individual measurement are given on zenodo.org (see section "Data Availability Statement" in the main text). Heat capacity changes ($\Delta C_{p,b}$) were calculated from the slope of the temperature dependence of the enthalpies, see also **Figure S26** and **Figure S27**. Given errors are standard errors from the linear regression.

host•guest complex	T(K)	K_a (M^{-1})	$\log K_a$[a]	ΔG_{exp}[b] (kcal mol^{-1})	ΔH_{exp}[c] (kcal mol^{-1})	$-T\Delta S_{exp}$[d] (kcal mol^{-1})	ΔS_{exp} (cal mol^{-1} K^{-1})	$\Delta C_{p,b}$ (cal mol^{-1} K^{-1})
CB7•1-AdOH[e]	278	-	-	-	−17.2[f]	-	-	−102 ± 6
	288	$3.44 \cdot 10^{10}$	10.5	−13.9	−17.7	3.8	−13.2	
	298[g]	$2.59 \cdot 10^{10}$	10.4	−14.2	−19.4	5.2	−17.4	
	308	$1.68 \cdot 10^{10}$	10.2	−14.4	−20.1	5.7	−18.5	
	318	$1.17 \cdot 10^{10}$	10.1	−14.7	−21.2	6.5	−20.4	
	328	$4.72 \cdot 10^{9}$	9.7	−14.5	−22.1	7.6	−23.2	
CB7•4-DAOH	278	$3.28 \cdot 10^{6}$	6.5	−8.3	−10.6	2.3	−8.3	−66 ± 5
	288	$9.99 \cdot 10^{6}$	7.0	−9.3	−11.8	2.5	−8.7	
	298	$8.59 \cdot 10^{6}$	6.9	−9.5	−12.1	2.6	−8.7	
	308	$1.77 \cdot 10^{7}$	7.3	−10.2	−13.0	2.7	−8.8	
	318	$3.08 \cdot 10^{7}$	7.5	−10.9	−13.5	2.7	−8.5	
	328	$2.53 \cdot 10^{7}$	7.4	−11.0	−14.0	3.0	−9.1	
CB7•4,9-DA(OH)$_2$	278	$1.08 \cdot 10^{7}$	7.0	−9.0	−9.9	0.9	−3.2	−135 ± 12
	288	$1.31 \cdot 10^{7}$	7.1	−9.4	−11.3	1.9	−6.6	
	298[g]	$1.02 \cdot 10^{7}$	7.0	−9.6	−12.6	3.0	−10.1	
	308	$1.08 \cdot 10^{7}$	7.0	−9.9	−13.7	3.8	−12.3	
	318	$1.83 \cdot 10^{7}$	7.3	−10.6	−14.5	3.9	−12.3	
	328	$1.04 \cdot 10^{7}$	7.0	−10.5	−17.2[f]	6.7	−20.4	
CB7•FeCp$_2$OH[h]	278	$1.15 \cdot 10^{10}$	10.1	−12.8	−19.9	6.9	−24.8	−64 ± 5
	288	$5.17 \cdot 10^{9}$	9.7	−12.8	−20.8	8.0	−27.8	
	298[g]	$2.45 \cdot 10^{9}$	9.4	−12.8	−21.0	8.2	−27.5	
	308	$1.42 \cdot 10^{9}$	9.2	−12.9	−21.8	8.9	−28.9	
	318	$4.56 \cdot 10^{8}$	8.7	−12.6	−22.3	9.7	−30.5	
	328	$2.41 \cdot 10^{8}$	8.4	−12.6	−23.1	10.5	−32.0	
CB7•Nan	278	$6.91 \cdot 10^{6}$	6.8	−8.7	−9.9	1.2	−4.3	−144 ± 5
	288	$5.67 \cdot 10^{6}$	6.8	−8.9	−11.5	2.6	−9.0	
	298	$3.37 \cdot 10^{6}$	6.5	−8.9	−12.7	3.8	−12.8	
	308	$2.43 \cdot 10^{6}$	6.4	−9.0	−13.9	4.9	−15.9	
	318	$1.53 \cdot 10^{6}$	6.2	−9.0	−15.6	6.6	−20.8	
	328	$1.16 \cdot 10^{6}$	6.1	−9.1	−17.3	8.2	−25.0	
CB7•Phe	278	$1.62 \cdot 10^{6}$	6.2	−7.9	−8.8	0.9	−3.2	−64 ± 8
	288	$1.40 \cdot 10^{6}$	6.1	−8.1	−9.0	0.9	−3.1	
	298	$1.03 \cdot 10^{6}$	6.0	−8.2	−9.5	1.3	−4.4	
	308	$7.75 \cdot 10^{5}$	5.9	−8.3	−10.1	1.8	−5.8	
	318	$5.06 \cdot 10^{5}$	5.7	−8.3	−11.5	3.2	−10.1	
	328	$3.39 \cdot 10^{5}$	5.5	−8.3	−11.7	3.4	−10.4	

	278	$1.62 \cdot 10^6$	6.2	−7.9	−7.1	−0.8	2.9	
	288	$9.88 \cdot 10^5$	6.0	−7.9	−8.6	0.7	−2.4	
CB7•HexOH	298	$7.36 \cdot 10^5$	5.9	−8.0	−9.6	1.6	−5.4	−89 ± 7
	308	$4.03 \cdot 10^5$	5.6	−7.9	−10.1	2.2	−7.1	
	318	$2.29 \cdot 10^5$	5.4	−7.8	−11.1	3.3	−10.4	
	328	$1.57 \cdot 10^5$	5.2	−7.8	−11.7	3.9	−11.9	

[a] Logarithmic binding affinity. [b] The experimental Gibbs free binding energy was obtained via $\Delta G_{\mathrm{exp}} = -\mathrm{RT}\ln K_{\mathrm{a}}$. [c] Experimental binding enthalpy. [d] Experimental entropic contributions to complex formation obtained via $-\mathrm{T}\Delta S_{\mathrm{exp}} = \Delta G_{\mathrm{exp}} - \Delta H_{\mathrm{exp}}$. [e] Binding affinity was determined by fluorescence-based IDA titration with MDAP as competitor. [f] Error ± 0.6 kcal mol^{-1}. [g] See also ref.[10]. [h] Binding affinity was determined by multistep ITC with phenylalanine as competitor. Errors (SD across at least three replicates) were 0.6 kcal mol^{-1} in ΔH_{exp} and ΔG_{exp}, and 1.2 kcal mol^{-1} in $-\mathrm{T}\Delta S_{\mathrm{exp}}$.

Table S2. Binding parameters for the complexation of guests with desalined CB8 in deionized water determined by ITC. The data was averaged from at least three dilution heat-corrected experiments. Typical errors (SD across at least three replicas) are 0.2 in $\log K_{\mathrm{a}}$, 0.5 kcal mol^{-1} in ΔH_{exp} and ΔG_{exp}, and 0.8 kcal mol^{-1} in $-\mathrm{T}\Delta S_{\mathrm{exp}}$. Errors (standard deviation across replicates, SD) were determined by repeating the titrations at least three times. Based on our extensive experience with ITC studies over the years, we employed these error estimates as an upper bound. Values for each individual measurement are given on zenodo.org (see section “Data Availability Statement” in the main text).Heat capacity changes ($\Delta C_{\mathrm{p,b}}$) were calculated from the slope of the temperature dependence of the enthalpies, see also **Figure S26** and **Figure S27**. Given errors are standard errors from the linear regression.

host•guest complex	T(K)	K_{a} (M^{-1})	$\log K_{\mathrm{a}}$[a]	ΔG_{exp}[b] (kcal mol^{-1})	ΔH_{exp}[c] (kcal mol^{-1})	$-\mathrm{T}\Delta S_{\mathrm{exp}}$[d] (kcal mol^{-1})	ΔS_{exp} (cal mol^{-1} K^{-1})	$\Delta C_{\mathrm{p,b}}$ (cal mol^{-1} K^{-1})
	278[e]	$1.19 \cdot 10^7$	7.1	−9.0	−6.9	−2.1	7.6	
	288	$9.58 \cdot 10^6$	7.0	−9.2	−7.6	−1.6	5.6	
CB8•1-AdOH	298[f]	$6.61 \cdot 10^6$	6.8	−9.3	−8.1	−1.2	4.0	−83 ± 7
	308	$7.63 \cdot 10^6$	6.9	−9.7	−9.0[g]	−0.7	2.3	
	318	$6.36 \cdot 10^6$	6.8	−9.9	−10.4	0.5	−1.6	
	328	$4.60 \cdot 10^6$	6.7	−10.0	−10.8	0.8	−2.4	
	278	$2.05 \cdot 10^7$	7.3	−9.3	−7.3	−2.0	7.2	
	288	$4.76 \cdot 10^6$	6.7	−8.8	−7.5	−1.3	4.5	
CB8•4-DAOH	298	$4.72 \cdot 10^6$	6.7	−9.1	−8.0	−1.1	3.7	−79 ± 13
	308	$1.06 \cdot 10^7$	7.0	−9.9	−8.5	−1.4	4.5	
	318	$7.46 \cdot 10^6$	6.9	−10.0	−9.9	−0.1	0.3	
	328	$1.01 \cdot 10^7$	7.0	−10.5	−11.3	0.8	−2.4	
	278	$1.43 \cdot 10^7$	7.2	−9.1	−5.6	−3.5	12.6	
	288	$8.04 \cdot 10^7$	6.9	−9.1	−6.8	−2.3	8.0	
CB8•4,9-DA(OH)$_2$	298[f]	$1.82 \cdot 10^7$	7.3	−9.9	−7.7	−2.2	7.4	−103 ± 5
	308	$5.51 \cdot 10^6$	6.7	−9.5	−8.9	−0.6	1.9	
	318	$4.64 \cdot 10^6$	6.7	−9.7	−10.1	0.4	−1.3	
	328	$3.95 \cdot 10^6$	6.6	−9.9	−10.6	0.7	−2.1	
	278	$1.19 \cdot 10^7$	7.1	−9.0	−11.4	2.4	−8.6	
	288	$9.58 \cdot 10^6$	7.0	−9.2	−11.9	2.7	−9.4	
CB8•3,9-TA(OH)$_2$	298	$9.27 \cdot 10^6$	7.0	−9.5	−12.7	3.2	−10.7	−97 ± 9
	308	$6.48 \cdot 10^6$	6.8	−9.6	−13.6	4.0	−13.0	
	318	$4.64 \cdot 10^6$	6.7	−9.7	−14.8	5.1	−16.0	
	328	$7.29 \cdot 10^6$	6.9	−10.3	−16.3	6.0	−18.3	

CB8•FeCp2OH	278	$8.28 \cdot 10^6$	6.9	−8.8	−11.4	2.6	−9.4	−55 ± 7
	288	$5.67 \cdot 10^6$	6.8	−8.9	−12.5	3.6	−12.5	
	298	$3.98 \cdot 10^6$	6.6	−9.0	−13.1	4.1	−13.8	
	308	$5.51 \cdot 10^6$	6.7	−9.5	−13.6	4.1	−13.3	
	318	$3.96 \cdot 10^6$	6.6	−9.6	−14.1	4.5	−14.2	
	328	$3.39 \cdot 10^6$	6.5	−9.8	−14.2	4.4	−13.4	
CB8•Nan	278[e]	$1.71 \cdot 10^7$	7.2	−9.2	−7.4	−1.8	6.5	−105 ± 9
	288	$9.58 \cdot 10^6$	7.0	−9.2	−8.0	−1.2	4.2	
	298	$9.27 \cdot 10^6$	7.0	−9.5	−8.9	−0.6	2.0	
	308	$5.51 \cdot 10^6$	6.7	−9.5	−10.1	0.6	−1.9	
	318	$3.96 \cdot 10^6$	6.6	−9.6	−10.9	1.3	−4.1	
	328	$1.83 \cdot 10^6$	6.2	−9.4	−12.8	3.4	−10.4	

[a] Logarithmic binding affinity. [b] The experimental Gibbs free binding energy was obtained via $\Delta G_{\mathrm{exp}} = -\mathrm{RT}\ln K_{\mathrm{a}}$. [c] Experimental binding enthalpy. [d] Experimental entropic contributions to complex formation obtained via $-\mathrm{T}\Delta S_{\mathrm{exp}} = \Delta G_{\mathrm{exp}} - \Delta H_{\mathrm{exp}}$. [e] An additional offset was fitted. [f] See also ref.[10]. [g] Error ± 0.7 kcal mol−1.

Table S3. Binding parameters for the complexation of guests with desalined β-CD in deionized water determined by ITC. The data was averaged from at least three dilution heat-corrected experiments. Typical errors (SD across at least three replicas) are 0.2 in $\log K_{\mathrm{a}}$, 0.5 kcal mol−1 in ΔH_{exp} and ΔG_{exp}, and 0.8 kcal mol−1 in $-\mathrm{T}\Delta S_{\mathrm{exp}}$. Errors (standard deviation across replicates, SD) were determined by repeating the titrations at least three times. Based on our extensive experience with ITC studies over the years, we employed these error estimates as an upper bound. Values for each individual measurement are given on zenodo.org (see section "Data Availability Statement" in the main text).Heat capacity changes ($\Delta C_{\mathrm{p,b}}$) were calculated from the slope of the temperature dependence of the enthalpies, see also **Figure S26** and **Figure S27**. Given errors are standard errors from the linear regression.

host•guest complex	T(K)	K_{a} (M−1)	$\log K_{\mathrm{a}}$[a]	ΔG_{exp}[b] (kcal mol−1)	ΔH_{exp}[c] (kcal mol−1)	$-\mathrm{T}\Delta S_{\mathrm{exp}}$[d] (kcal mol−1)	ΔS_{exp} (cal mol−1 K−1)	$\Delta C_{\mathrm{p,b}}$ (cal mol−1 K−1)
β-CD•1-AdOH	278	$1.07 \cdot 10^5$	5.0	−6.4	−4.3	−2.1	7.6	−95 ± 8
	288	$8.56 \cdot 10^4$	4.9	−6.5	−5.3	−1.4	4.2	
	298	$5.85 \cdot 10^4$	4.7	−6.5	−6.5	0.0	0.0	
	308	$4.09 \cdot 10^4$	4.6	−6.5	−7.5	1.0	−3.2	
	318	$2.50 \cdot 10^4$	4.4	−6.4	−8.7	2.3	−7.2	
	328	$2.50 \cdot 10^4$	4.4	−6.6	−8.8	2.2	−6.7	
β-CD•4,9-DA(OH)2	278	$3.18 \cdot 10^5$	5.5	−7.0	−6.8	−0.2	0.7	−61 ± 9
	288	$2.05 \cdot 10^5$	5.3	−7.0	−8.0	1.0	−3.5	
	298	$1.15 \cdot 10^5$	5.1	−6.9	−8.9	2.0	−6.7	
	308	$7.87 \cdot 10^4$	4.9	−6.9	−9.5	2.6	−8.4	
	318	$5.52 \cdot 10^4$	4.7	−6.9	−9.7	2.8	−8.8	
	328	$3.96 \cdot 10^4$	4.6	−6.9	−10.0	3.1	−9.5	
β-CD•FeCp2OH	278	$2.53 \cdot 10^4$	4.4	−5.6	−5.7	0.1	−0.4	−61 ± 9
	288	$1.49 \cdot 10^4$	4.2	−5.5	−6.7	1.2	−4.2	
	298	$1.08 \cdot 10^4$	4.0	−5.5	−7.7	2.2	−7.4	
	308	$7.99 \cdot 10^3$	3.9	−5.5	−8.3	2.8	−9.1	
	318	$6.02 \cdot 10^3$	3.8	−5.5	−8.5	3.0	−9.4	
	328	$4.62 \cdot 10^3$	3.7	−5.5	−8.8[e]	3.3	−10.1	

[a] Logarithmic binding affinity. [b] The experimental Gibbs free binding energy was obtained via $\Delta G_{\mathrm{exp}} = -\mathrm{RT}\ln K_{\mathrm{a}}$. [c] Experimental binding enthalpy. [d] Experimental entropic contributions to complex formation obtained via $-\mathrm{T}\Delta S_{\mathrm{exp}} = \Delta G_{\mathrm{exp}} - \Delta H_{\mathrm{exp}}$. [e] Error ± 0.7 kcal mol−1.

Binding Thermodynamics at Room Temperature

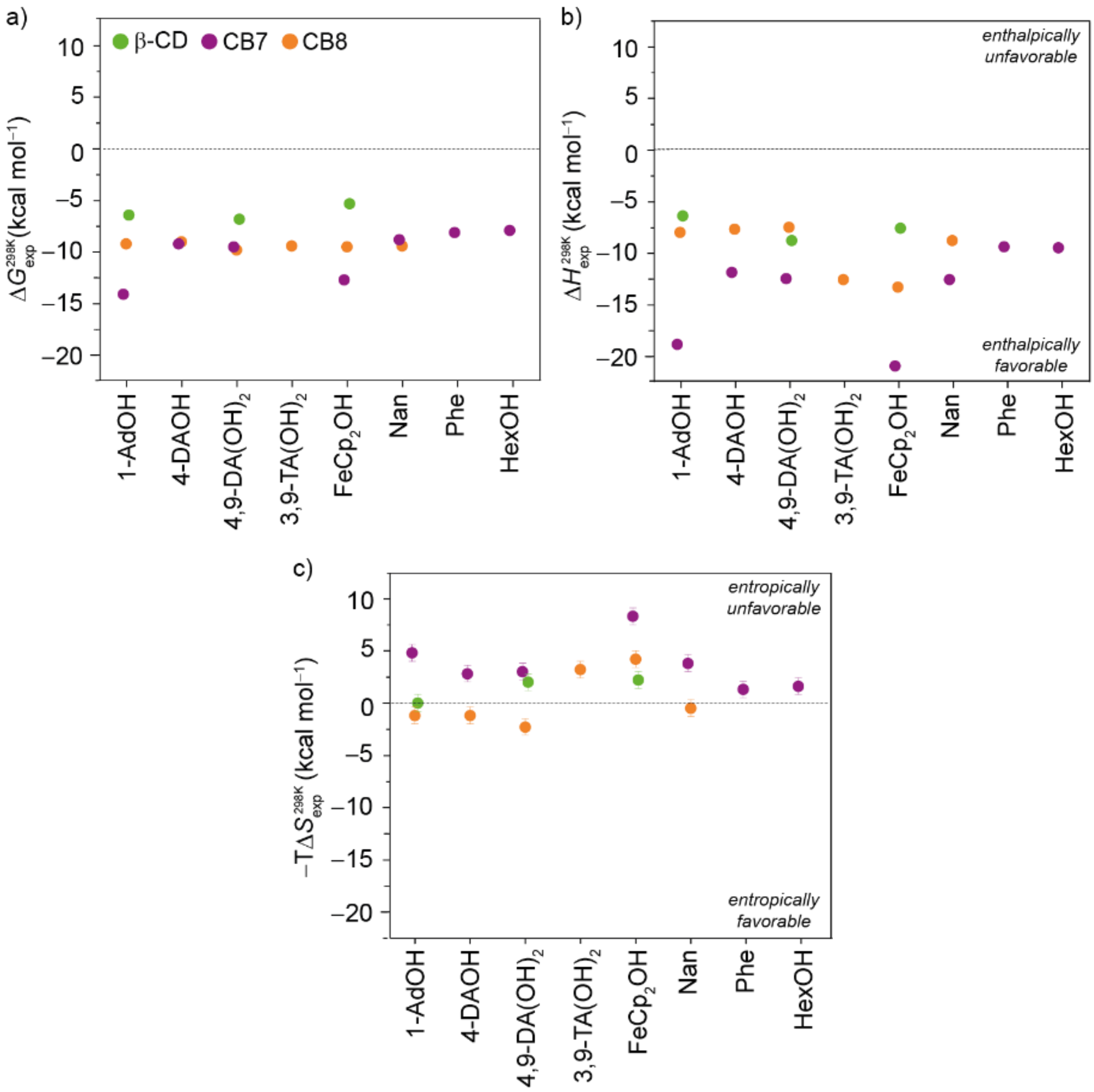

Figure S1. Graphical representation of (a) the binding free energy (ΔG_{exp}^{298K}), (b) the binding enthalpy (ΔH_{exp}^{298K}), and (c) the entropic contribution ($-T\Delta S_{exp}^{298K}$) of the complex formation of all investigated host-guest systems at 298 K. See **Tables S1-S3** for the individual values.

Reproducibility

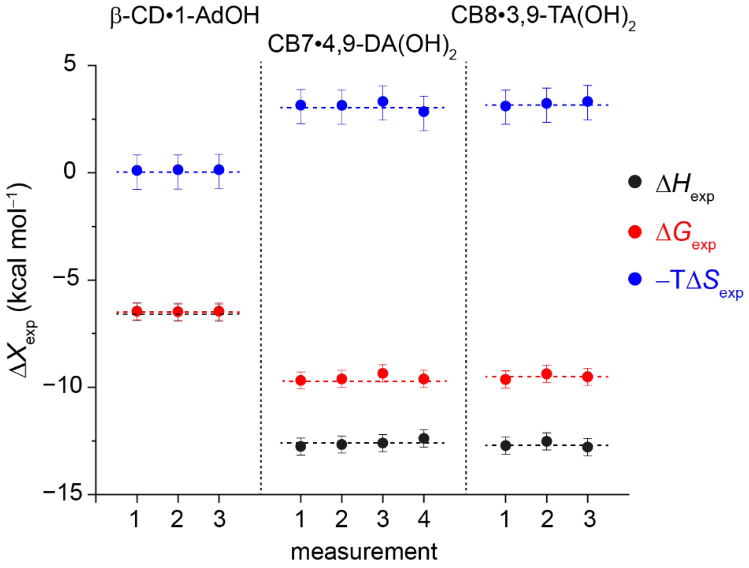

Figure S2. Individually measured repetitions and standard deviations (SD) of the determined thermodynamic binding parameters ΔH_{exp} (black), ΔG_{exp} (red), and $-\mathrm{T}\Delta S_{\mathrm{exp}}$ (blue) exemplarily shown for the complex formation of β-CD•1-AdOH, CB7•4,9-DA$(OH)_2$, and CB8•3,9-TA$(OH)_2$.

ITC Experiments with Cucurbit[7]uril

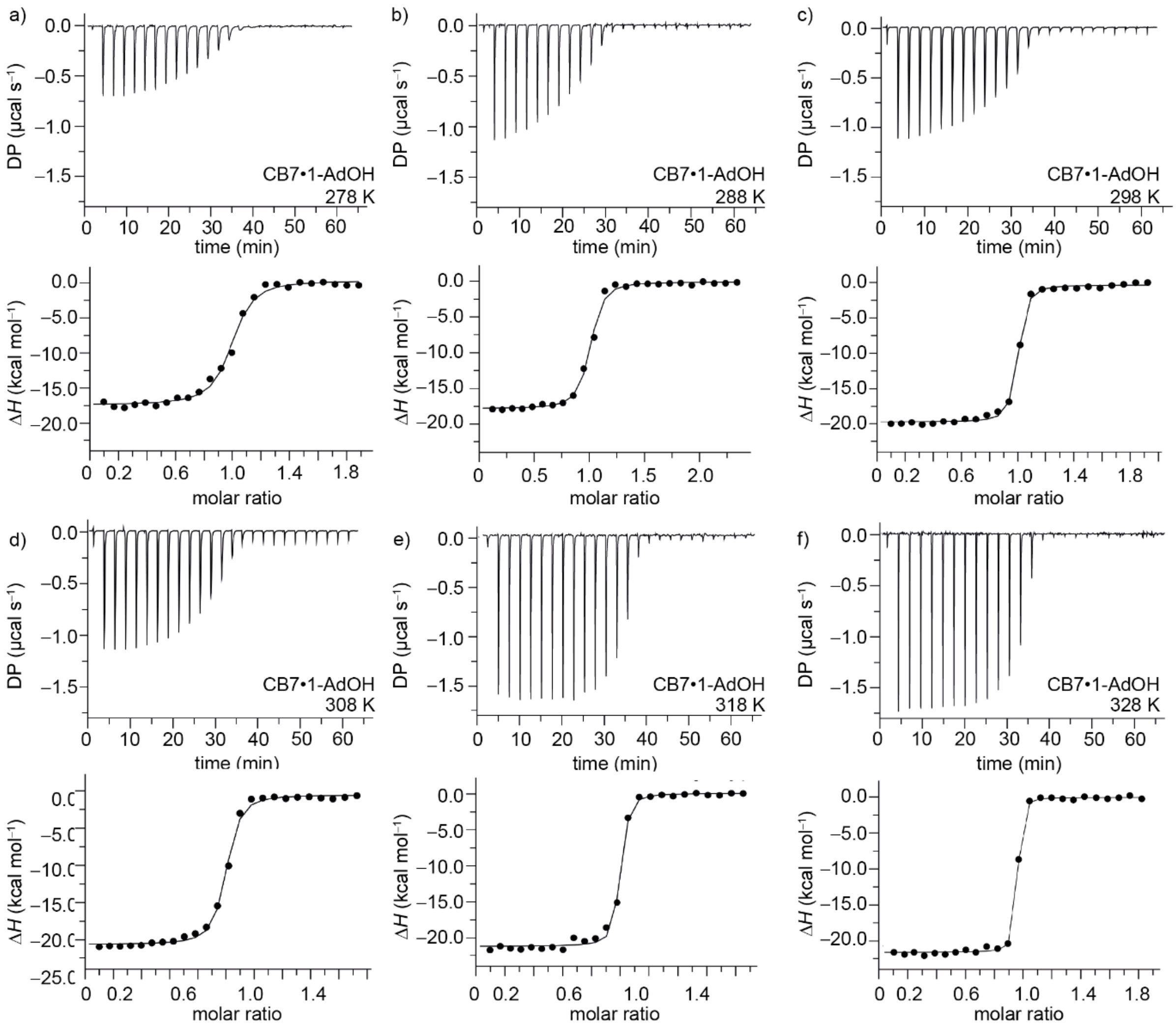

Figure S3. ITC isotherms (dilution heat corrected) for the direct titration of 1-AdOH (c = 0 - 70 µM) to CB7 (c = 34 µM) from 278 to 328 K. Raw data of the measurements are uploaded on zenodo.org (DOI: 10.5281/zenodo.7082003).

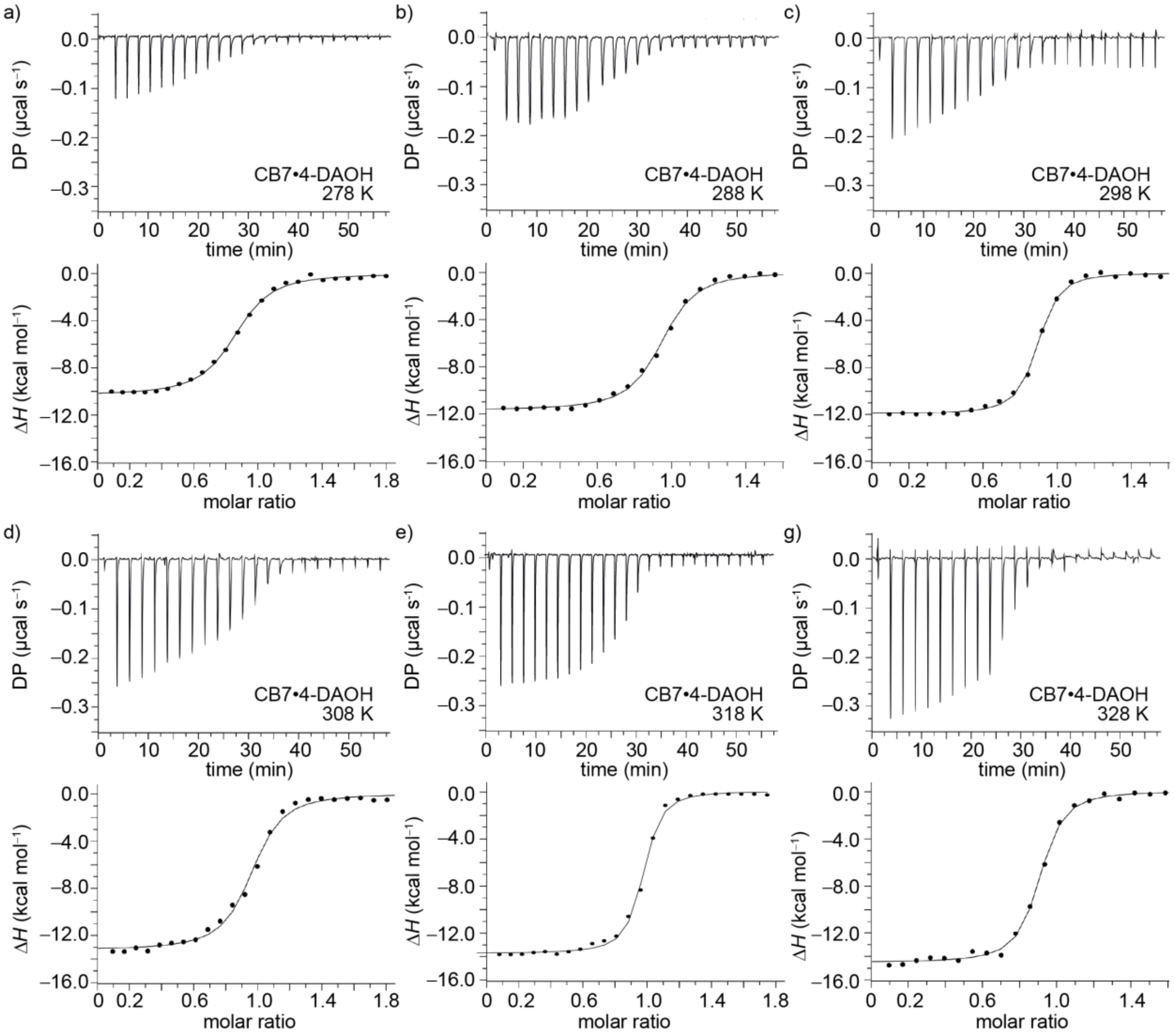

Figure S4. ITC isotherms (dilution heat corrected) for the titration of 4-DAOH (c = 0 - 70 µM) to CB7 (c = 34 µM) in a temperature range from 278 to 328 K. Raw data of the measurements are uploaded on zenodo.org (DOI: 10.5281/zenodo.7082003).

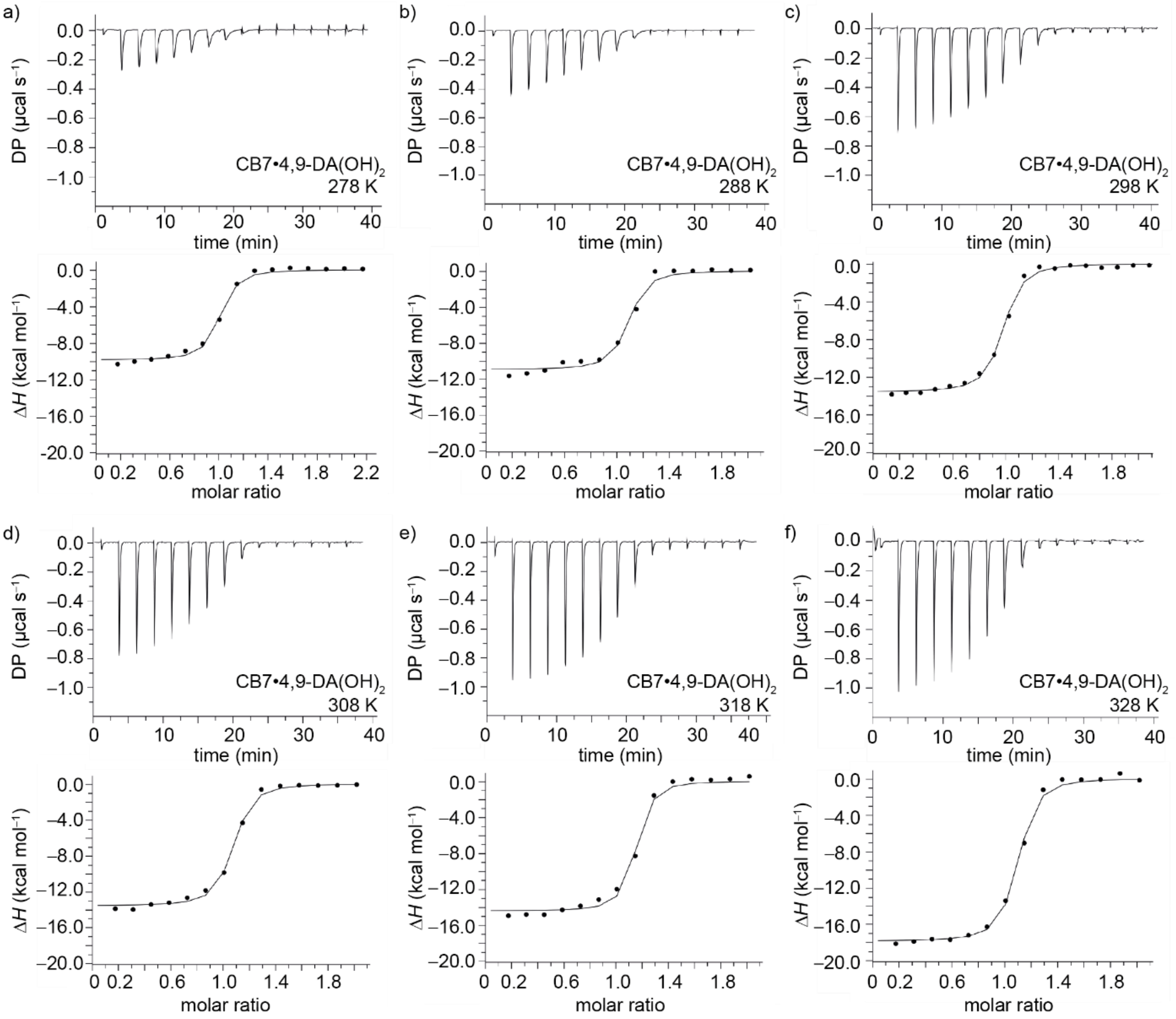

Figure S5. ITC isotherms (dilution heat corrected) for the titration of 4,9-DA(OH)$_2$ (c = 0 - 90 µM) to CB7 (c = 42.5 µM) in a temperature range from 278 to 328 K. Raw data of the measurements are uploaded on zenodo.org (DOI: 10.5281/zenodo.7082003).

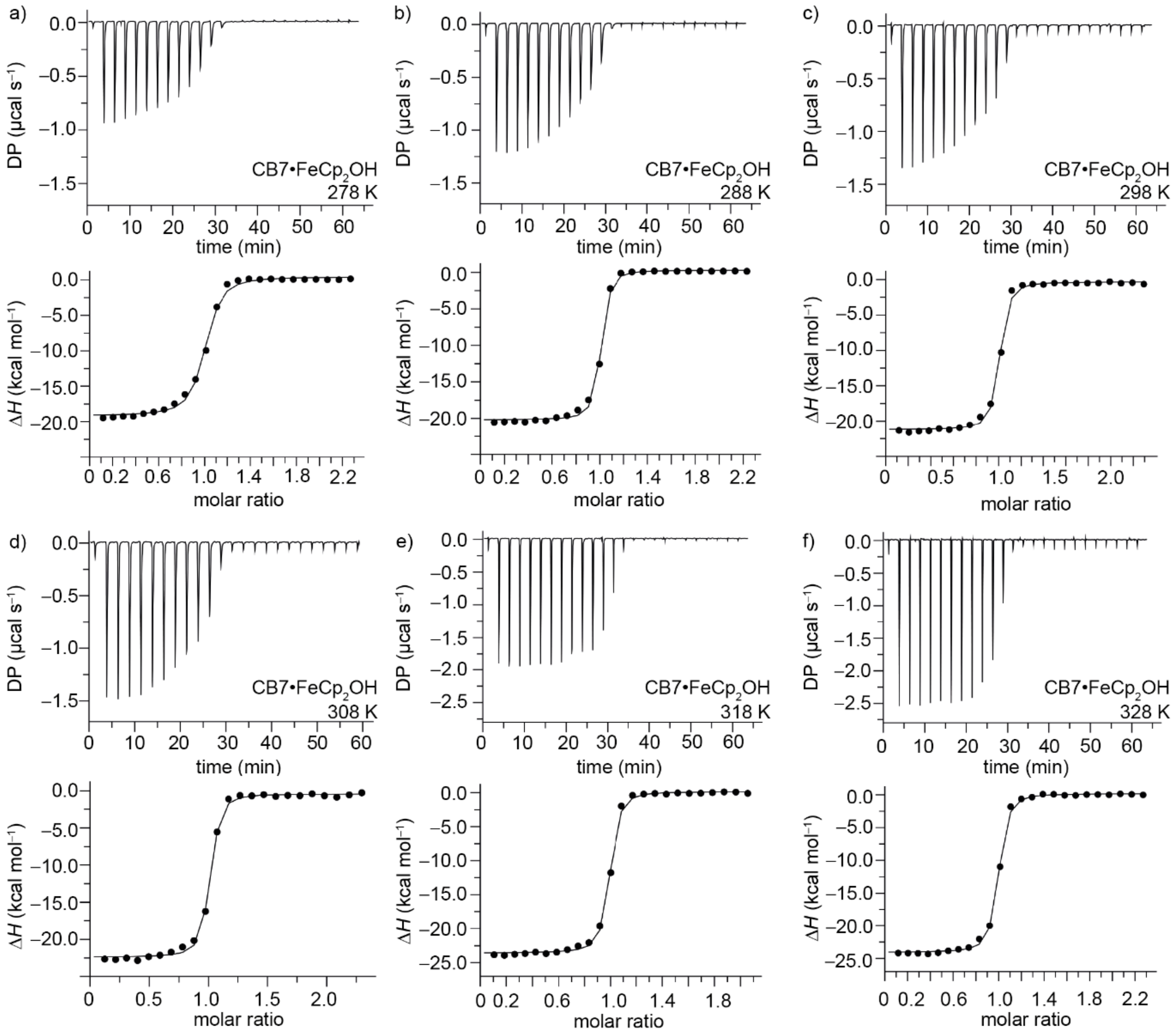

Figure S6. ITC isotherms (dilution heat corrected) for the direct titration of $FeCp_2OH$ (c = 0 – 70 µM) to CB7 (c = 34 µM) in a temperature range from 278 to 328 K. Raw data of the measurements are uploaded on zenodo.org (DOI: 10.5281/zenodo.7082003).

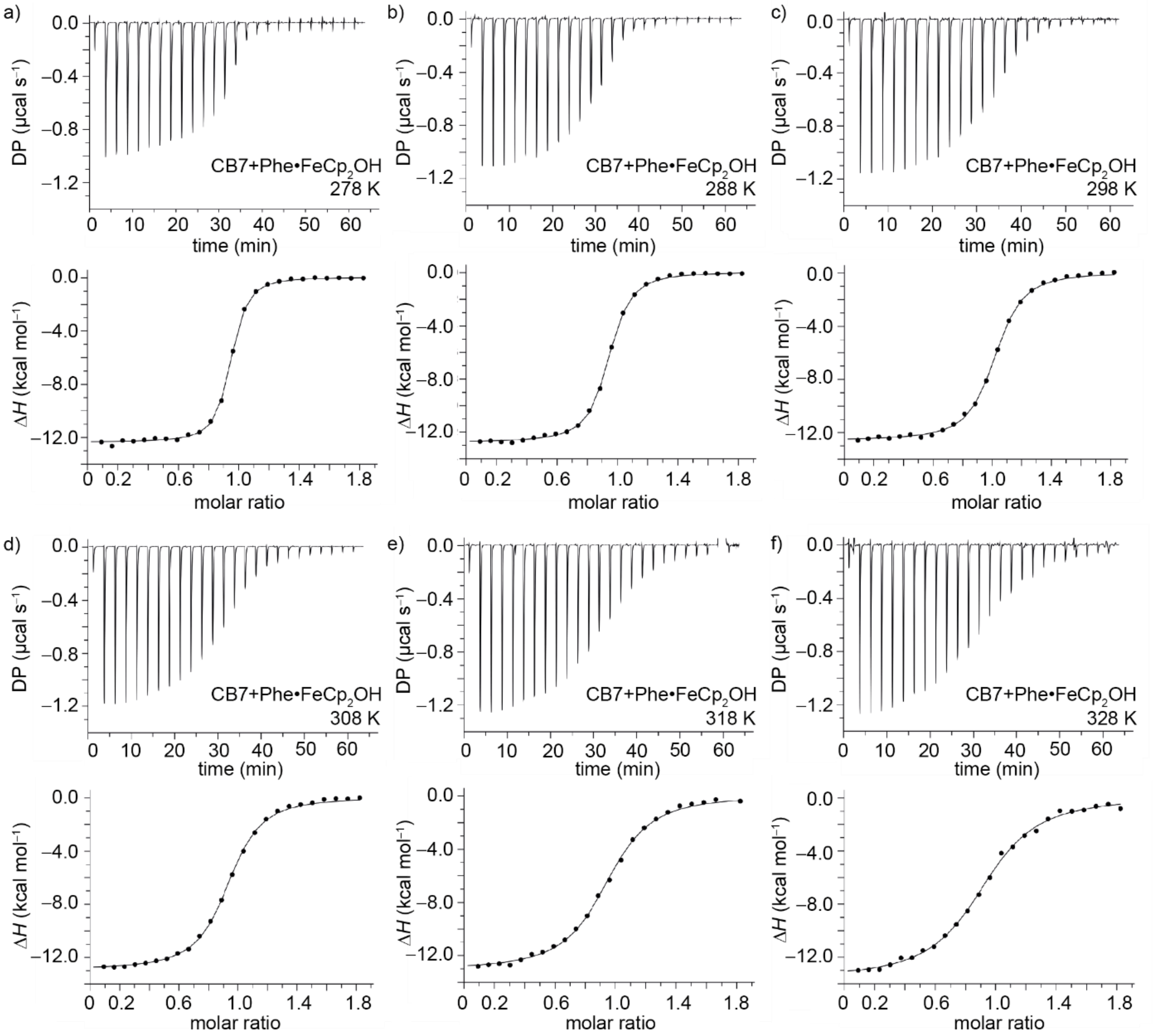

Figure S7. ITC isotherms (dilution heat corrected) for the titration of $FeCp_2OH$ (c = 0 - 100 µM) to a mixture of CB7 (c = 62.5µM) and Phe (c = 1.5 mM) in a temperature range from 278 to 328 K. Raw data of the measurements are uploaded on zenodo.org (DOI: 10.5281/zenodo.7082003).

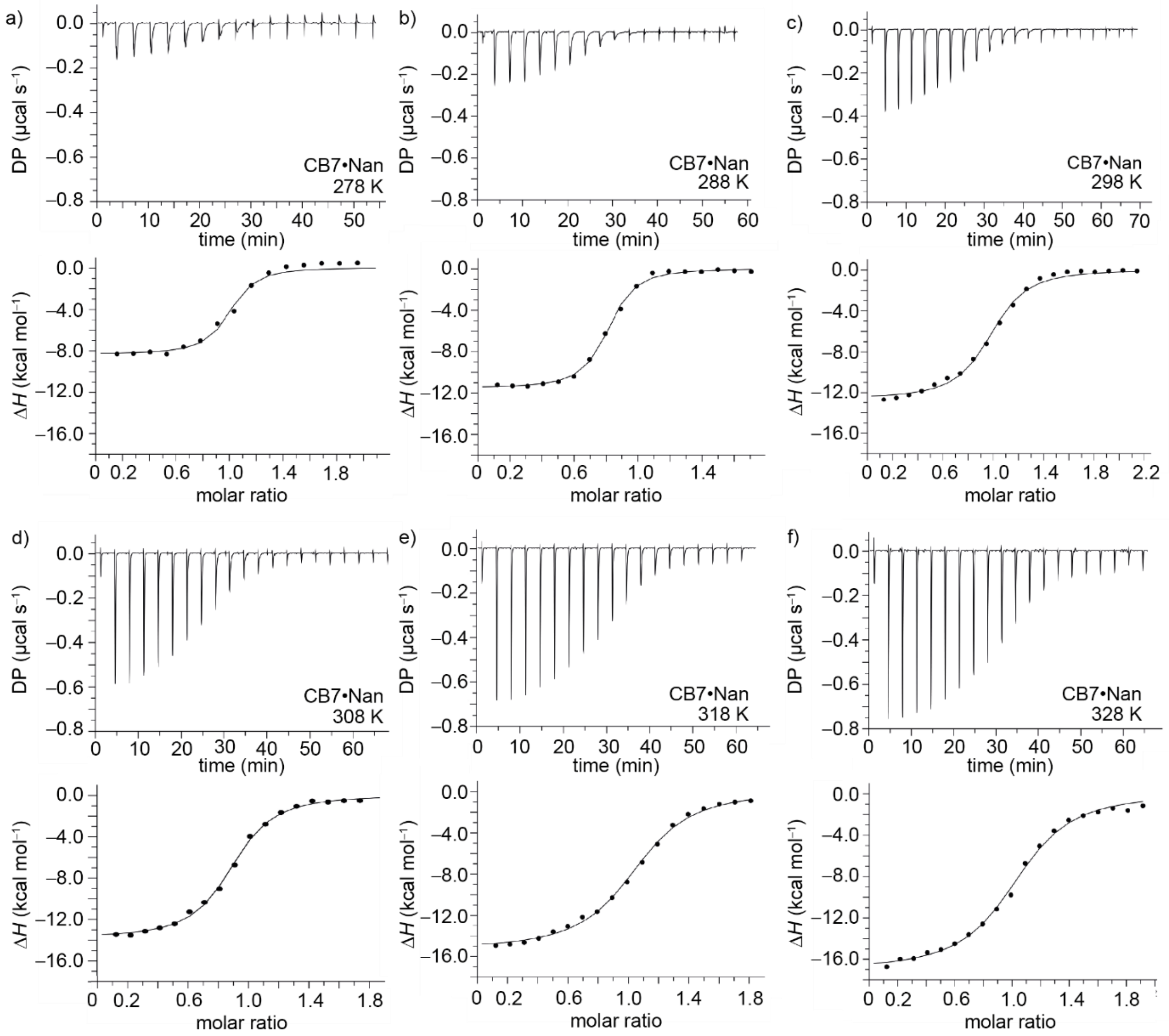

Figure S8. ITC isotherms (dilution heat corrected) for the titration of Nan (c = 0 - 40 µM) to CB7 (c = 20 µM) in a temperature range from 278 to 328 K. Raw data of the measurements are uploaded on zenodo.org (DOI: 10.5281/zenodo.7082003).

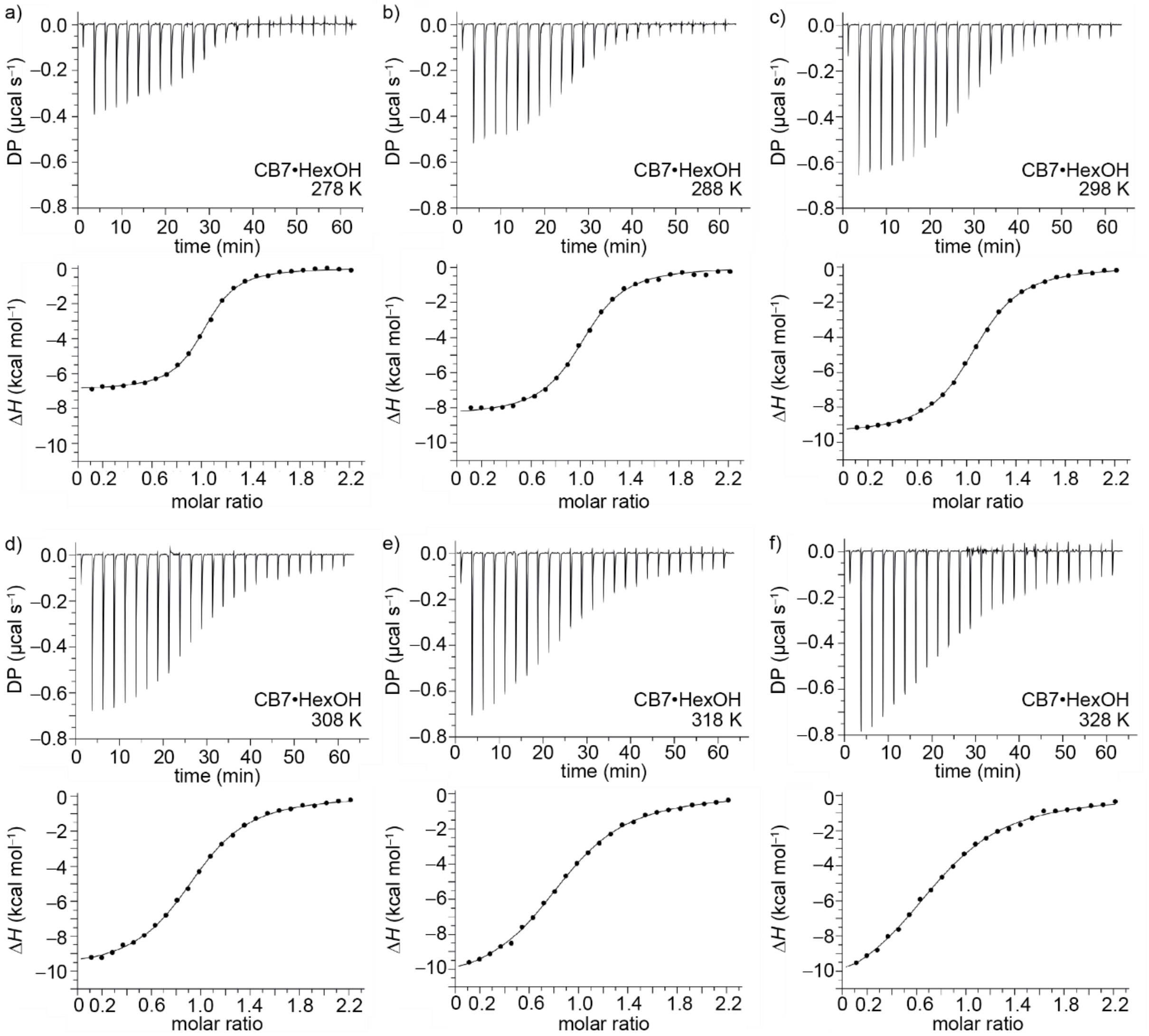

Figure S9. ITC isotherms (dilution heat corrected) for the titration of HexOH (c = 0 - 90 µM) to CB7 (c = 40 µM) in a temperature range from 278 to 328 K. Raw data of the measurements are uploaded on zenodo.org (DOI: 10.5281/zenodo.7082003).

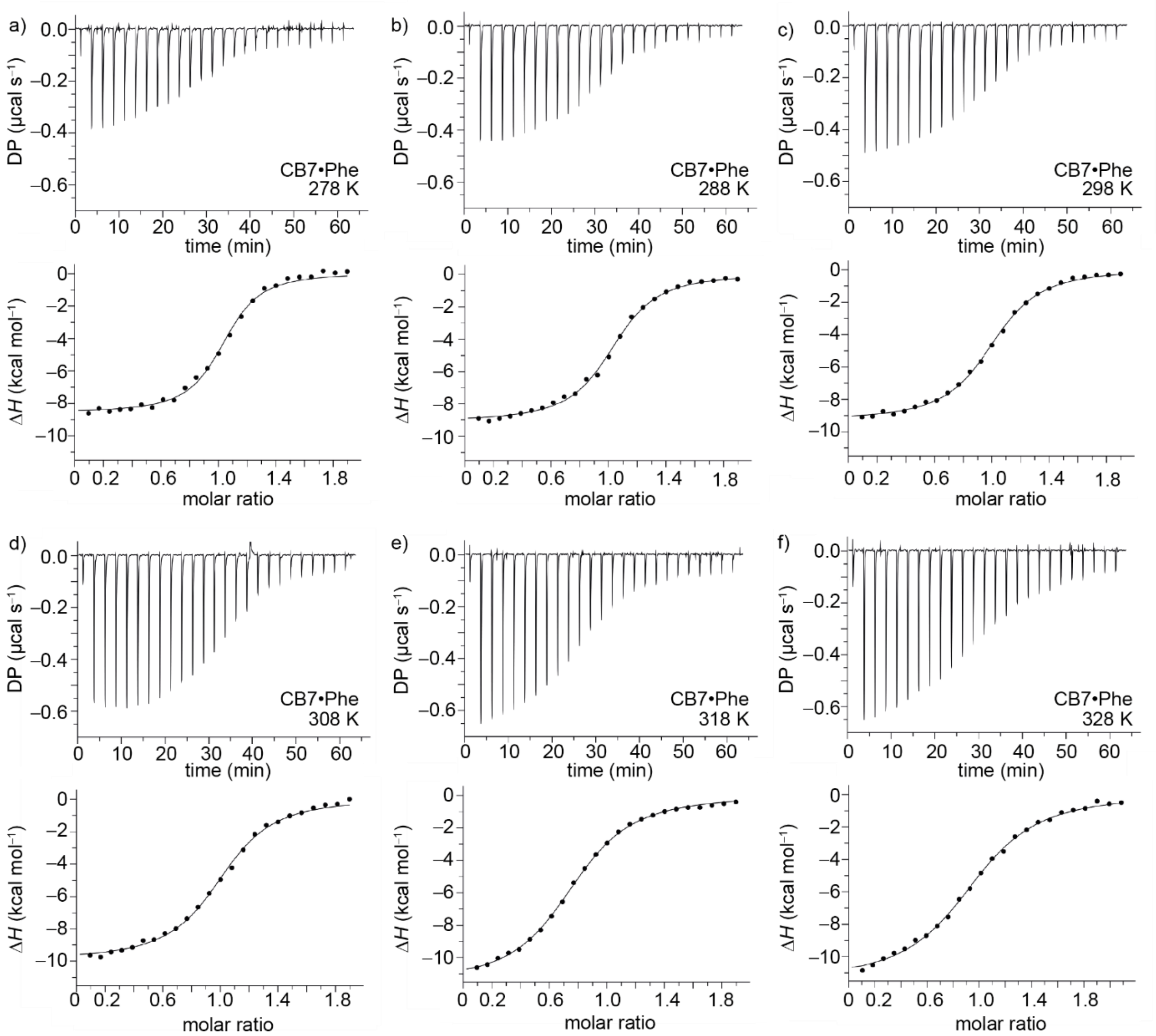

Figure S10. ITC isotherms (dilution heat corrected) for the titration of Phe (c = 0 - 63 µM) to CB7 (c = 35 µM) in a temperature range from 278 to 328 K. Raw data of the measurements are uploaded on zenodo.org (DOI: 10.5281/zenodo.7082003).

ITC Experiments with Cucurbit[8]uril

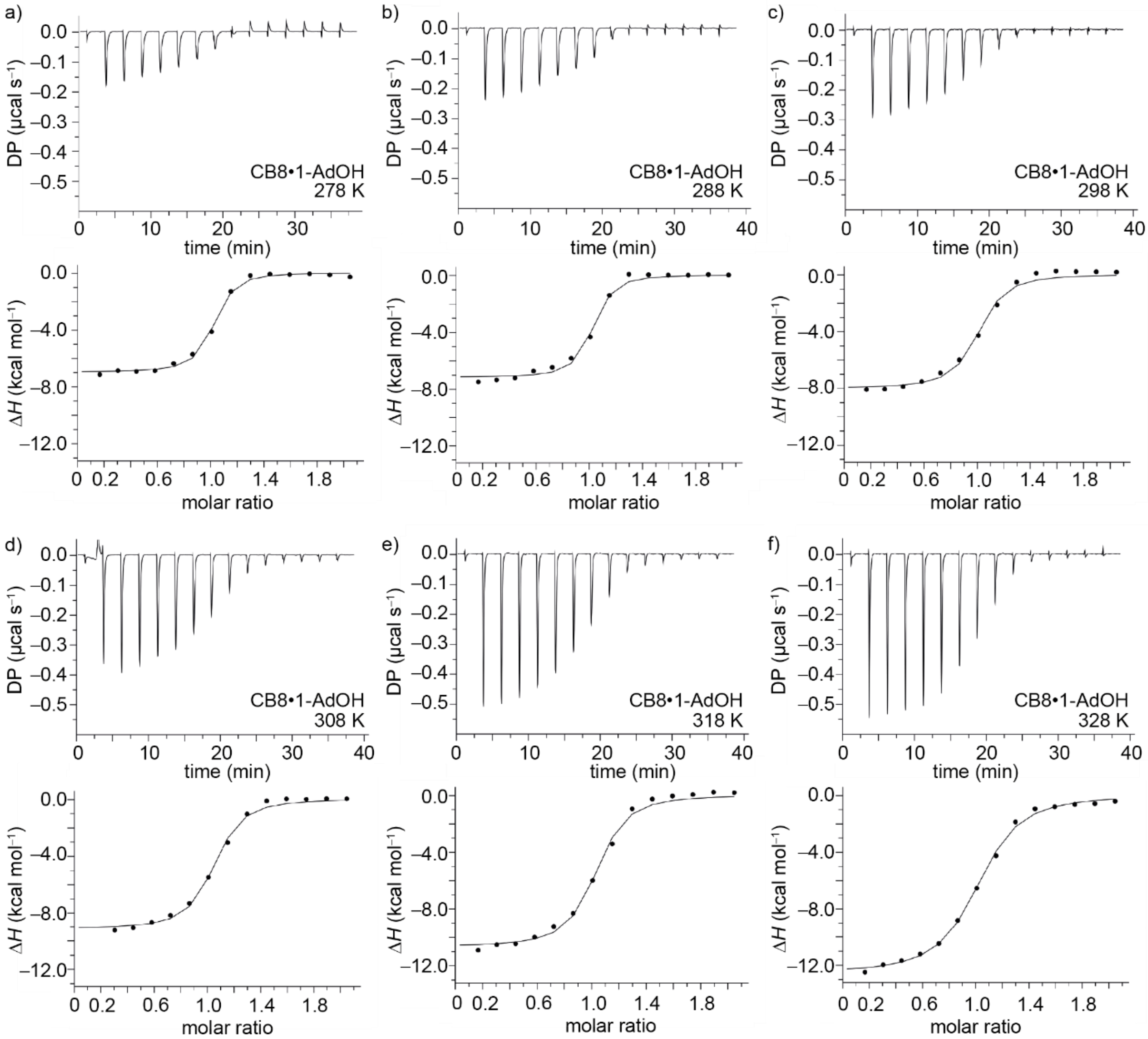

Figure S11. ITC isotherms (dilution heat corrected) for the titration of 1-AdOH (c = 0 - 35 µM) to CB8 (c = 16 µM) in a temperature range from 278 to 328 K. Raw data of the measurements are uploaded on zenodo.org (DOI: 10.5281/zenodo.7082003).

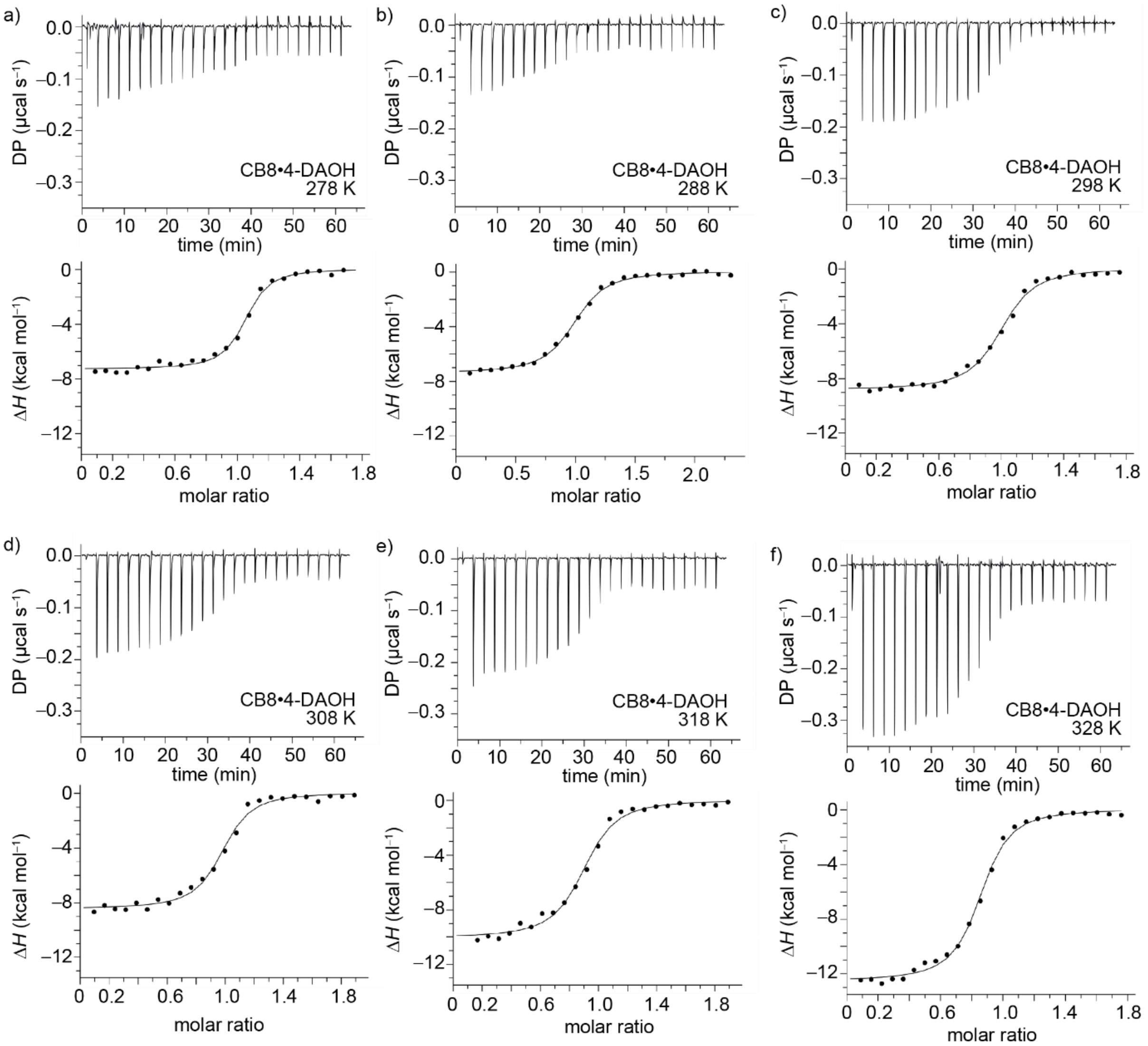

Figure S12. ITC isotherms (dilution heat corrected) for the titration of 4-DAOH (c = 0 - 20 µM) to CB8 (c = 11 µM) in a temperature range from 278 to 328 K. Raw data of the measurements are uploaded on zenodo.org (DOI: 10.5281/zenodo.7082003).

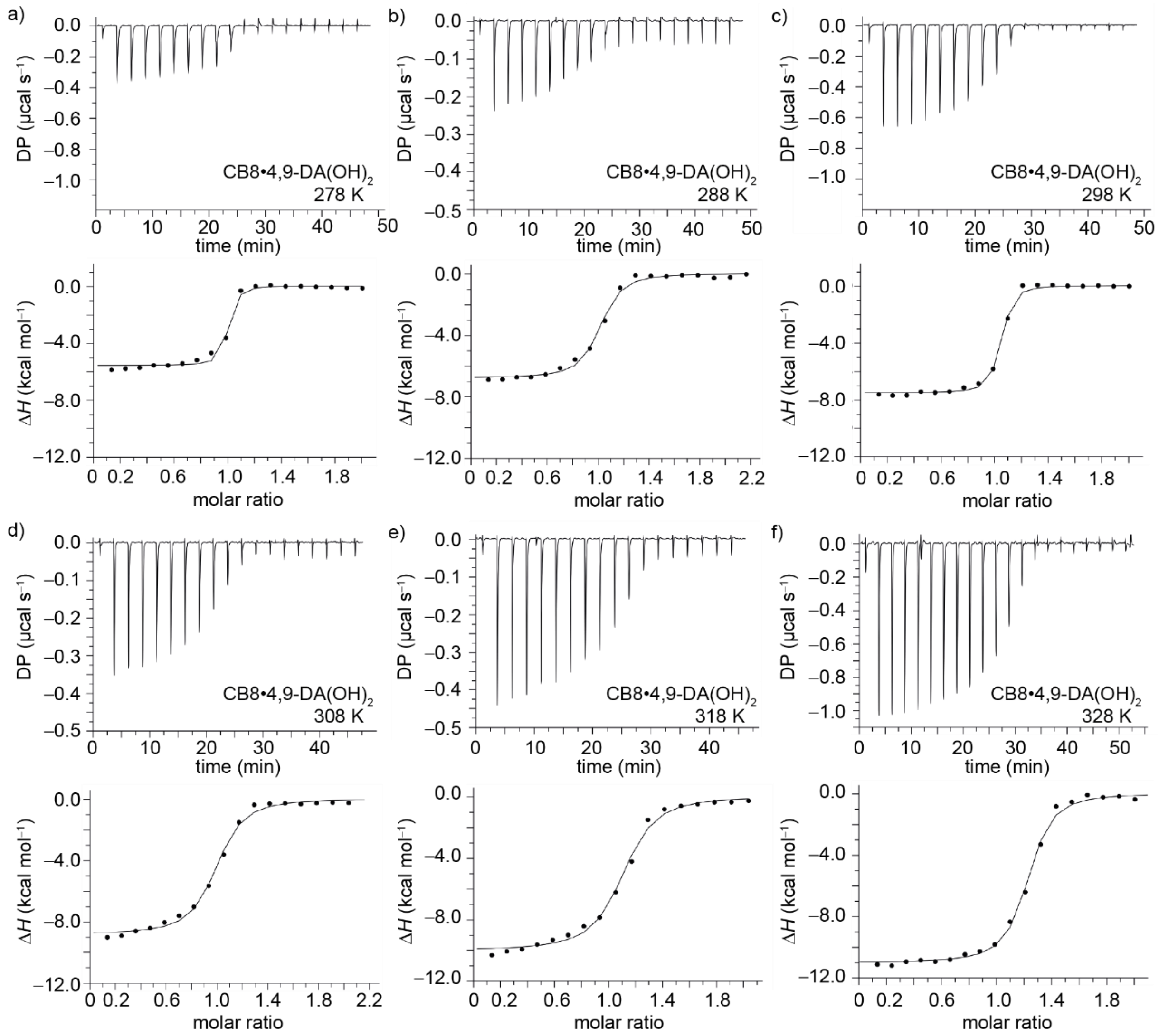

Figure S13. ITC isotherms (dilution heat corrected) for the titration of 4,9-DA(OH)$_2$ (c = 0 - 45 µM) to CB8 (c = 19.5 µM) in a temperature range from 278 to 328 K. Raw data of the measurements are uploaded on zenodo.org (DOI: 10.5281/zenodo.7082003).

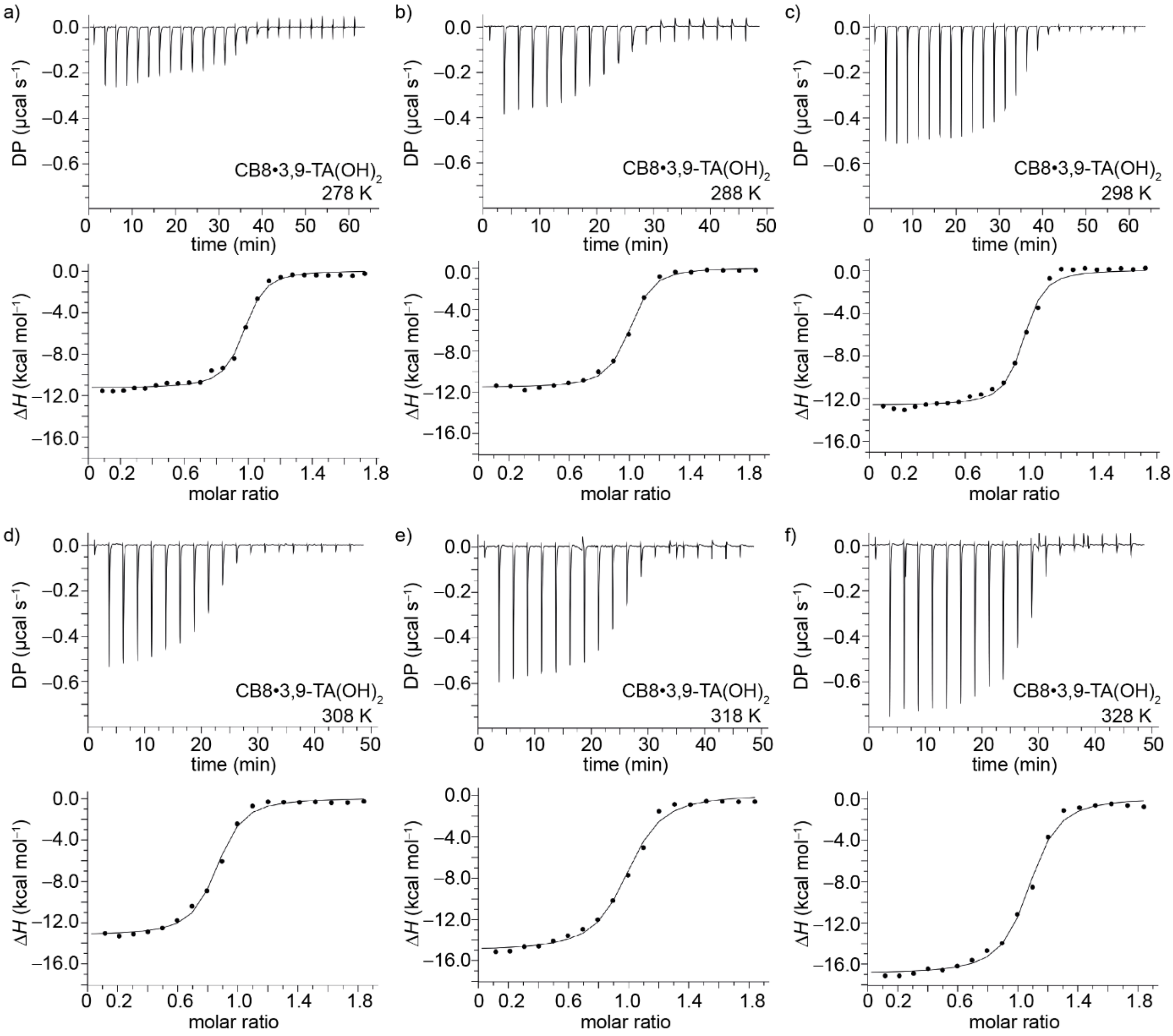

Figure S14. ITC isotherms (dilution heat corrected) for the titration of 3,9-TA$(OH)_2$ (c = 0 - 40 µM) to CB8 (c = 21.5 µM) in a temperature range from 278 to 328 K. Raw data of the measurements are uploaded on zenodo.org (DOI: 10.5281/zenodo.7082003).

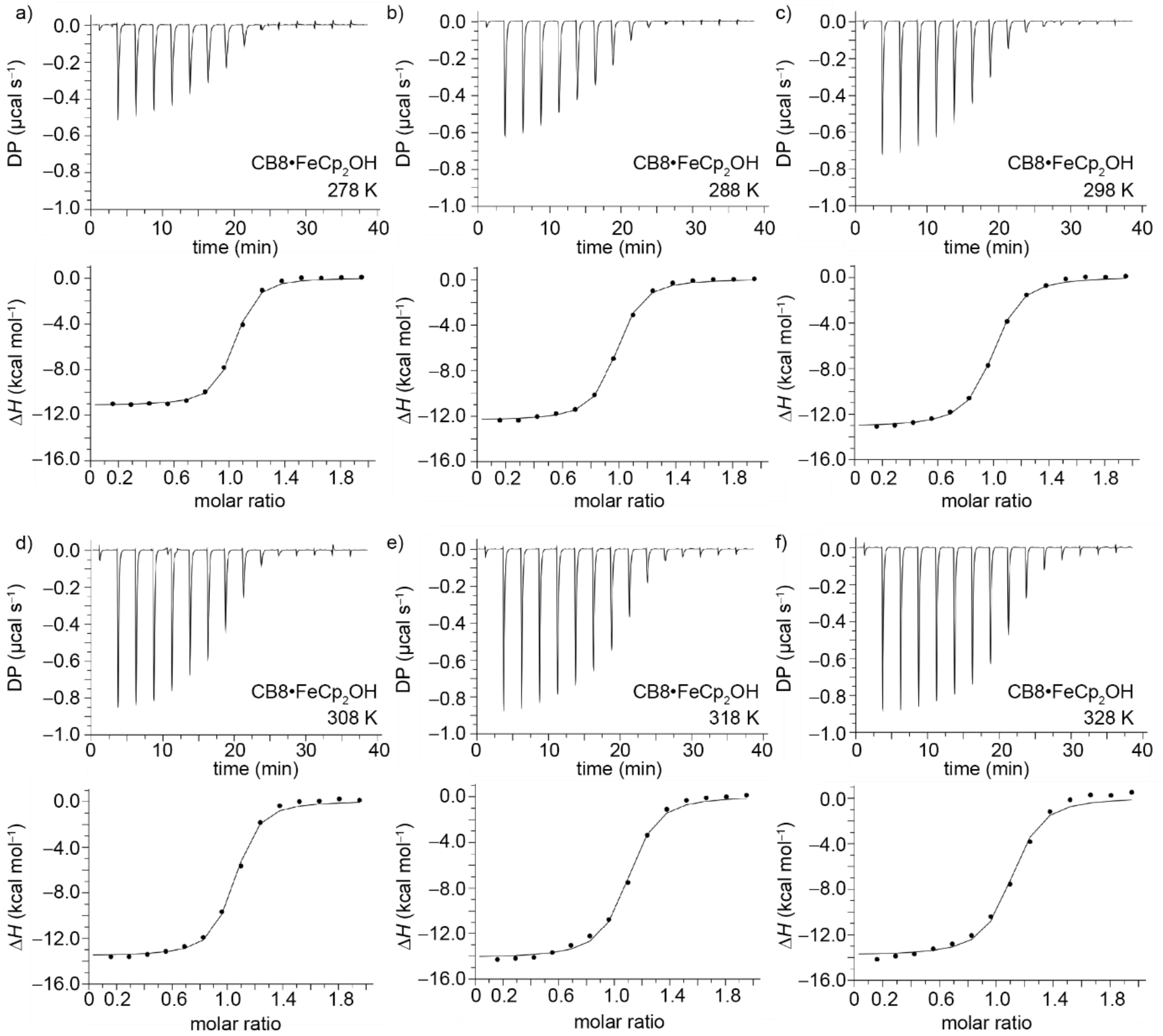

Figure S15. ITC isotherms (dilution heat corrected) for the titration of $FeCp_2OH$ (c = 0 - 55 µM) to CB8 (c = 26 µM) in a temperature range from 278 to 328 K. Raw data of the measurements are uploaded on zenodo.org (DOI: 10.5281/zenodo.7082003).

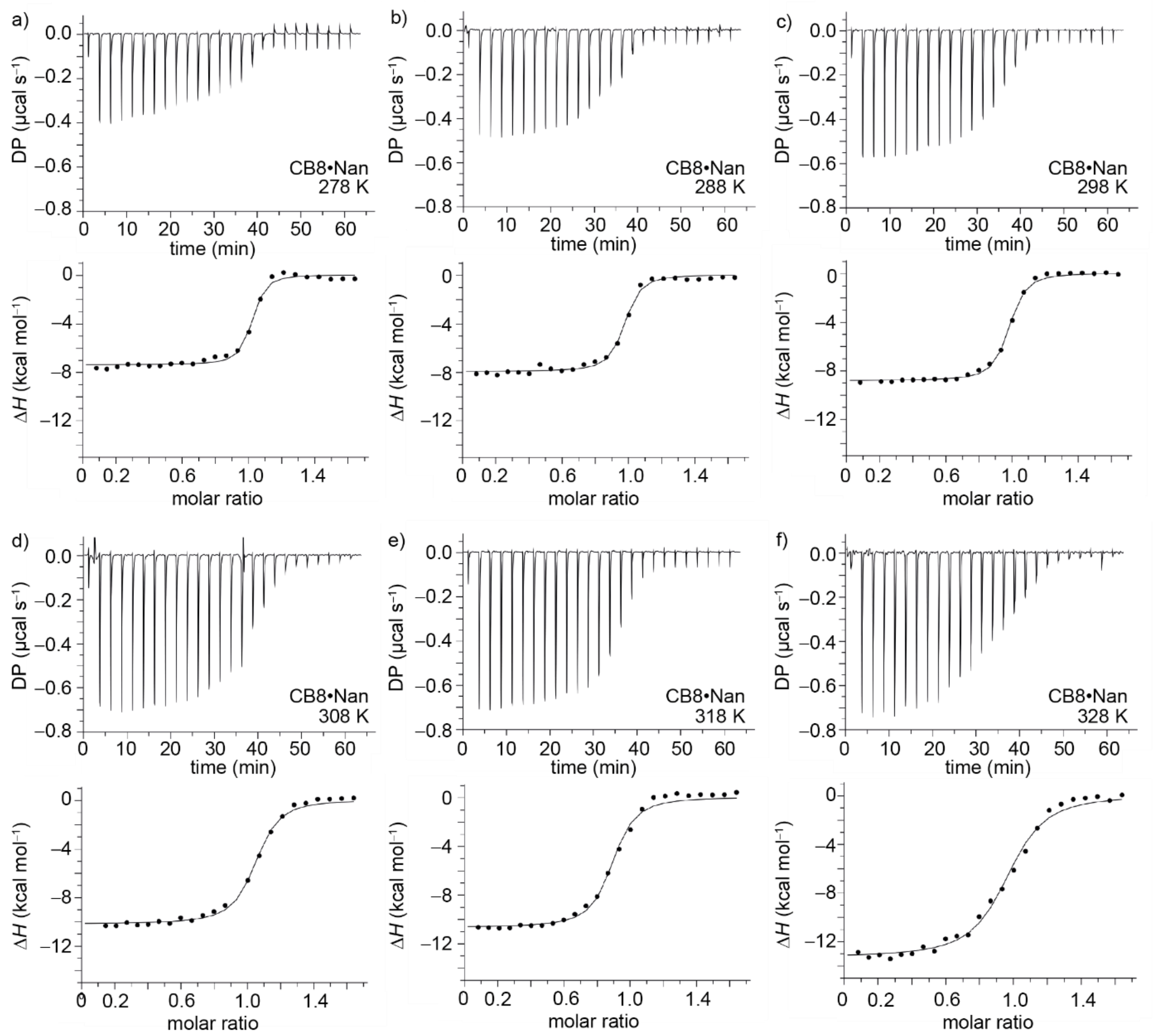

Figure S16. ITC isotherms (dilution heat corrected) for the titration of Nan (c = 0 - 82 µM) to CB8 (c = 48 µM) in a temperature range from 278 to 328 K. Raw data of the measurements are uploaded on zenodo.org (DOI: 10.5281/zenodo.7082003).

ITC Experiments with β-Cyclodextrin

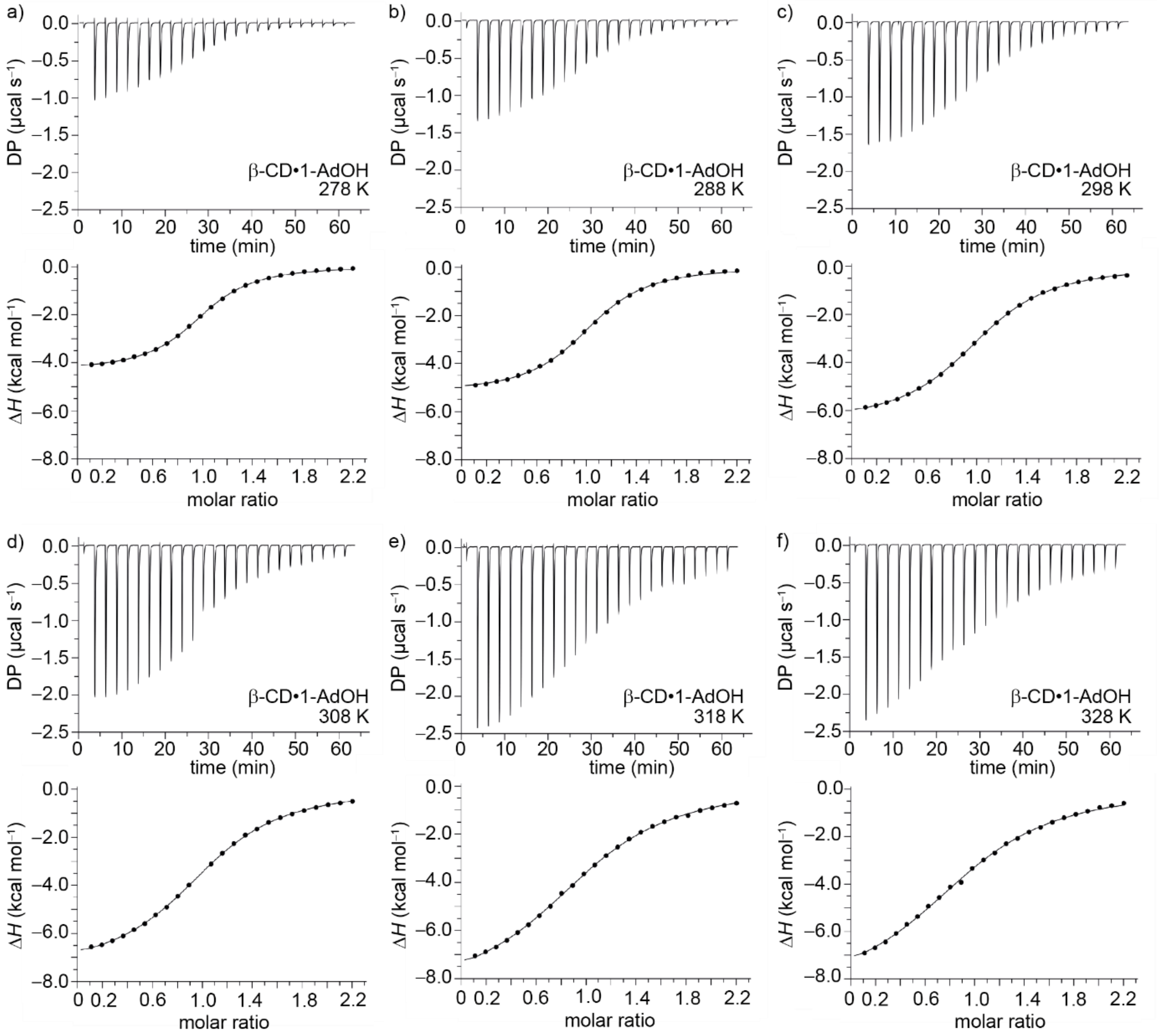

Figure S17. ITC isotherms (dilution heat corrected) for the titration of 1-AdOH (c = 0 - 450 µM) to β-CD (c = 196 µM) in a temperature range from 278 to 328 K. Raw data of the measurements are uploaded on zenodo.org (DOI: 10.5281/zenodo.7082003).

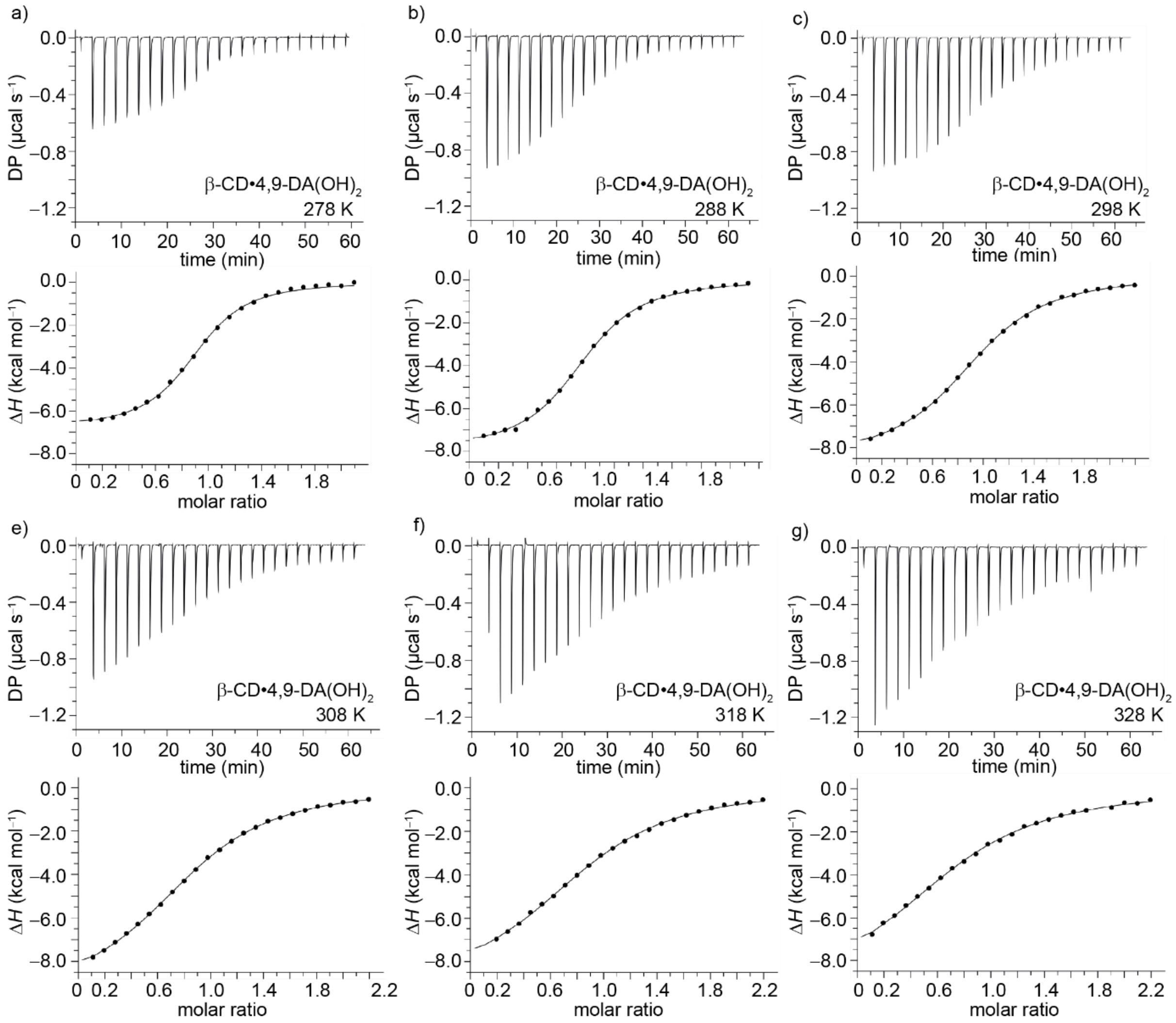

Figure S18. ITC isotherms (dilution heat corrected) for the titration of 4,9-DA(OH)$_2$ (c = 0 - 65 µM) to β-CD (c = 29 µM) in a temperature range from 278 to 328 K. Raw data of the measurements are uploaded on zenodo.org (DOI: 10.5281/zenodo.7082003).

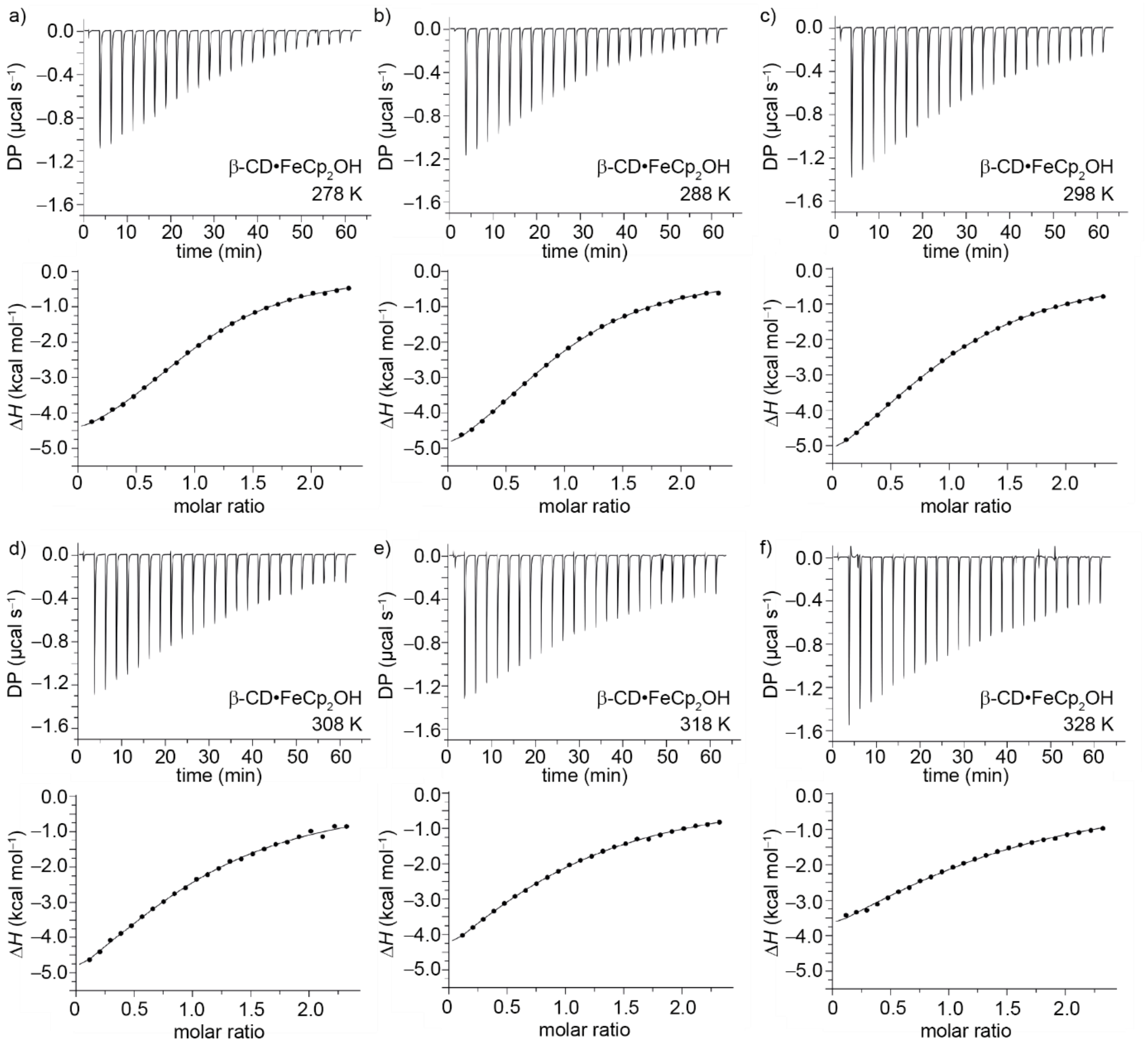

Figure S19. ITC isotherms (dilution heat corrected) for the titration of $FeCp_2OH$ (c = 0 - 441 µM) to β-CD (c = 196 µM) in a temperature range from 278 to 328 K. Raw data of the measurements are uploaded on zenodo.org (DOI: 10.5281/zenodo.7082003).

Fluorescence-Based Assays

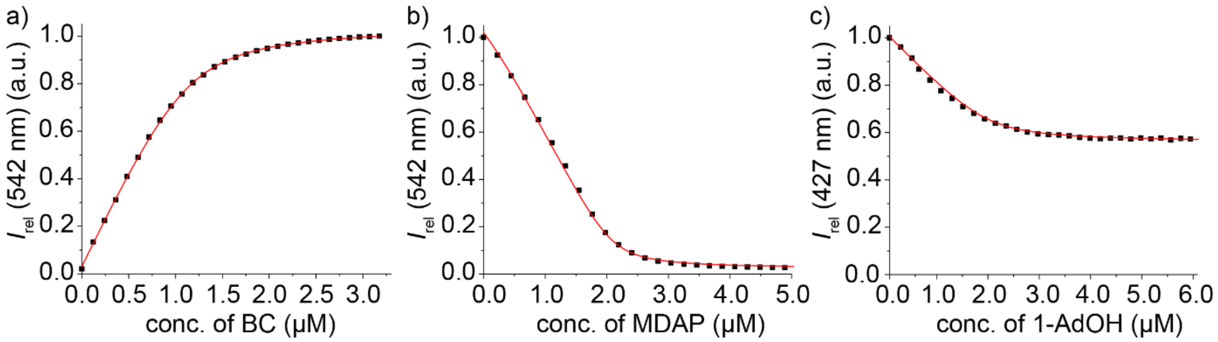

Figure S20. (a) Fluorescence intensity at 542 nm (λ_{ex} = 440 nm) after the attainment of the equilibrium (30 s reaction time) as a function of BC concentration at 298 K; c(CB7) = 1.15 μM. (b) Fluorescence intensity at 542 nm (λ_{ex} = 421 nm) after the attainment of the equilibrium (15 s reaction time) as a function of MDAP concentration at 298 K; c(CB7) = 2.2 μM; c(BC) = 2.5 μM. (c) Fluorescence intensity at 427 nm (λ_{ex} = 378 nm) after the attainment of the equilibrium (300 s reaction time) as a function of 1-AdOH concentration at 298 K; c(CB7) = 2.0 μM; c(MDAP) = 3.0 μM.

Table S4. Binding affinities of the indicator dyes determined by fluorescence-based titration experiments, see **Table S1** for the values for 1-AdOH.

host•guest complex / temperature		278 K	288 K	298 K	308 K	318 K	328 K
CB7•BC	K_a (M^{-1})	$1.7 \cdot 10^7$	$1.5 \cdot 10^7$	$1.1 \cdot 10^7$	$6.7 \cdot 10^6$	$5.4 \cdot 10^6$	$3.3 \cdot 10^6$
	$\log K_a$	7.2	7.2	7.0	6.8	6.7	6.5
CB7•MDAP	K_a (M^{-1})	$1.6 \cdot 10^9$	$1.7 \cdot 10^9$	$9.7 \cdot 10^8$	$6.4 \cdot 10^8$	$4.8 \cdot 10^8$	$3.0 \cdot 10^8$
	$\log K_a$	9.2	9.2	9.0	8.8	8.7	8.5

Binding Affinities

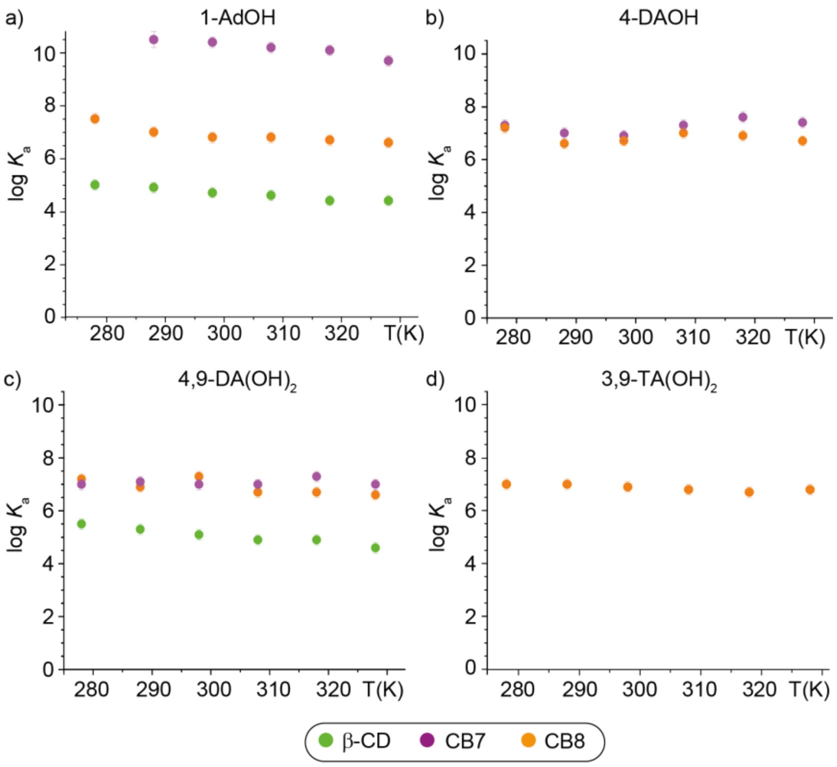

Figure S21. Graphical representation of the determined binding affinities (shown as decadic logarithm of K_a) for the guests (a) 1-AdOH, (b) 4-DAOH, (c) 4,9-DA(OH)$_2$, and (d) 3,9-TA(OH)$_2$ with β-CD (green), CB7 (violet), and CB8 (orange) in a temperature range from 278 to 328 K (for CB7•1-AdOH only 288 to 328 K). Error bars calculated as SD from at least three repetition experiments are shown. See **Table S1** - **Table S3** for individual values.

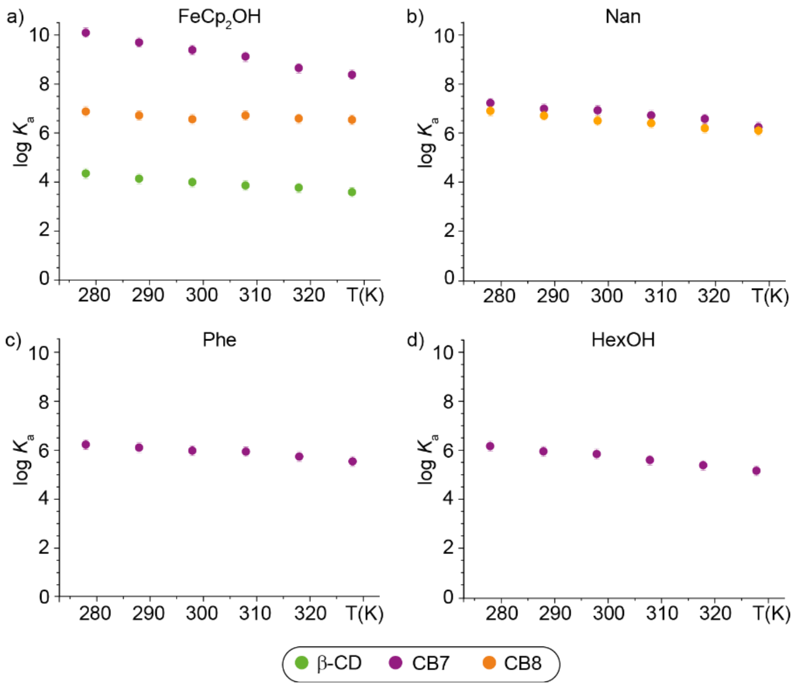

Figure S22. Graphical representation of the determined binding affinities (shown as decadic logarithm of K_a) for the guests (a) FeCp$_2$OH, (b) Nan, (c) Phe, and (d) HexOH with β-CD (green), CB7 (violet), and CB8 (orange) in a temperature range from 278 to 328 K. Error bars calculated as SD from at least three repetition experiments are shown. See **Table S1** - **Table S3** for individual values.

Standard Complexation Parameters

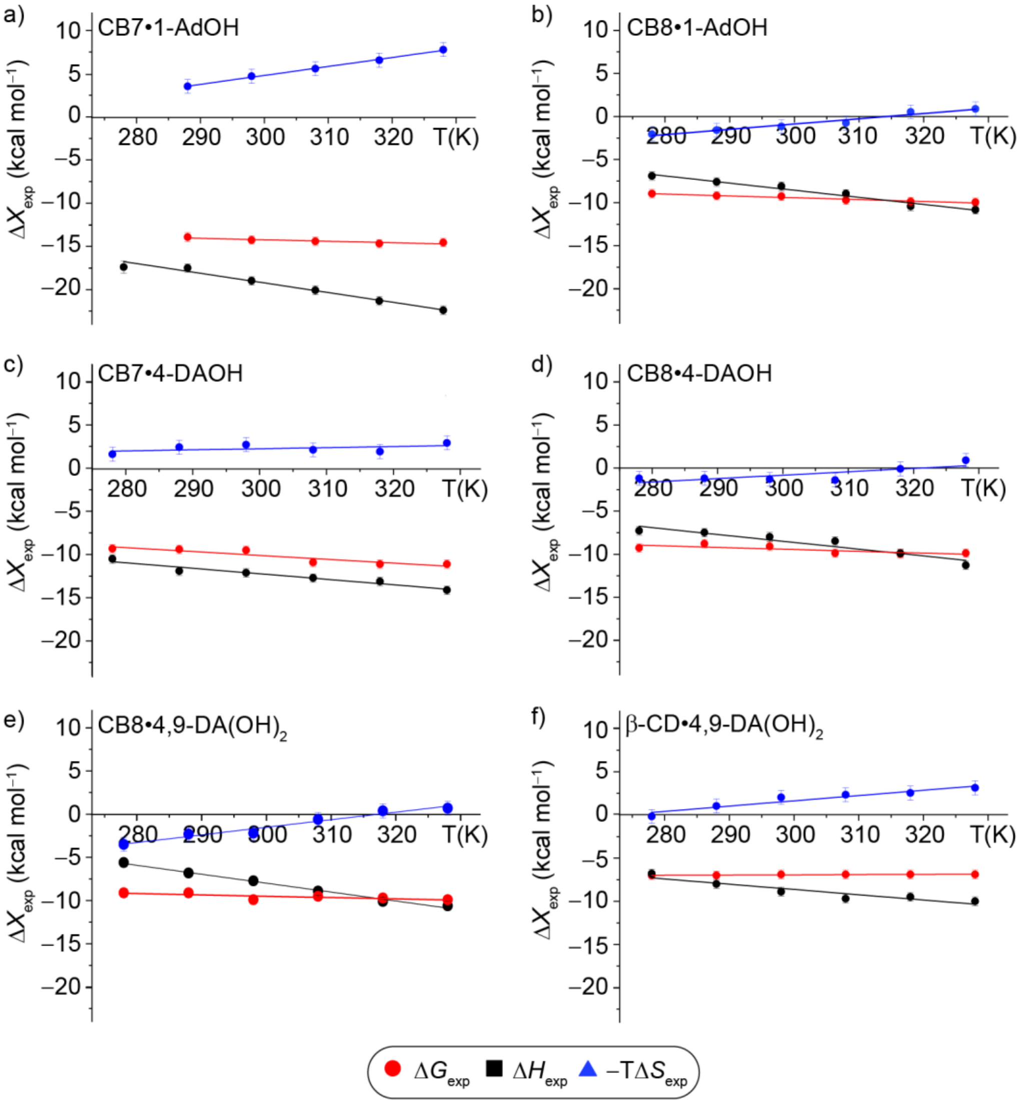

Figure S23. The temperature dependence of the standard complexation parameters for (a) CB7•1-AdOH, (b) CB8•1-AdOH, (c) CB7•4-DAOH, (d) CB8•4-DAOH, (e) CB8•4,9-DA(OH)$_2$, and (f) β-CD•4,9-DA(OH)$_2$ in a temperature range from 278 to 328 K. Error bars calculated as SD from at least three repetition experiments are shown. See **Table S1** - **Table S3** for individual values.

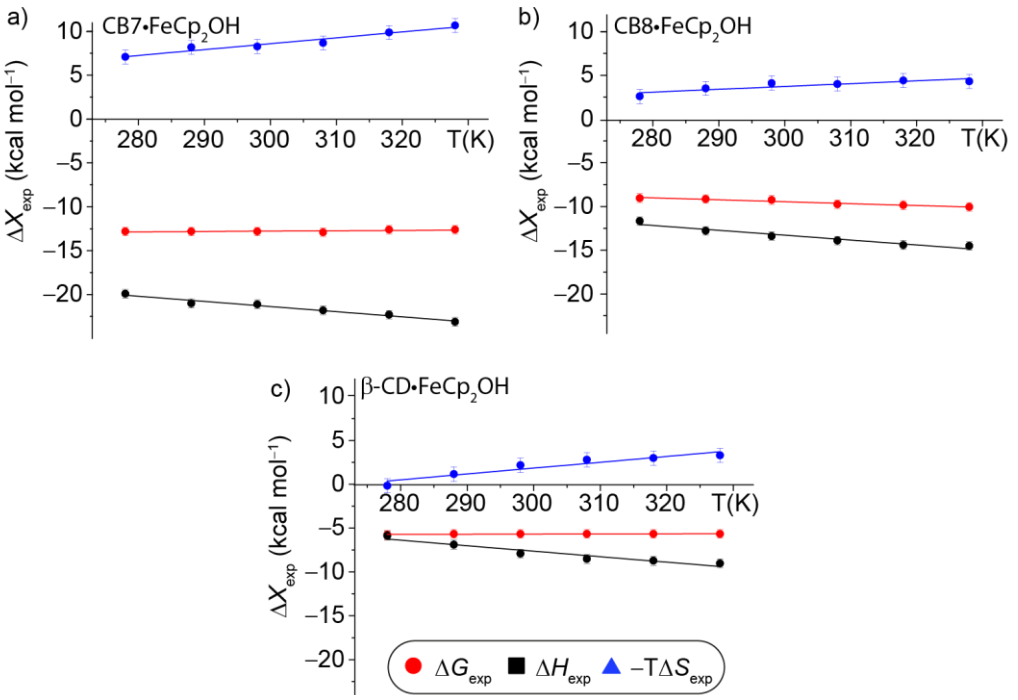

Figure S24. The temperature dependence of the standard complexation parameters for (a) CB7•$FeCp_2OH$, (b) CB8•$FeCp_2OH$, and (c) β-CD•$FeCp_2OH$ in a temperature range from 278 to 328 K. Error bars calculated as SD from at least three repetition experiments are shown. See **Table S1** - **Table S3** for individual values.

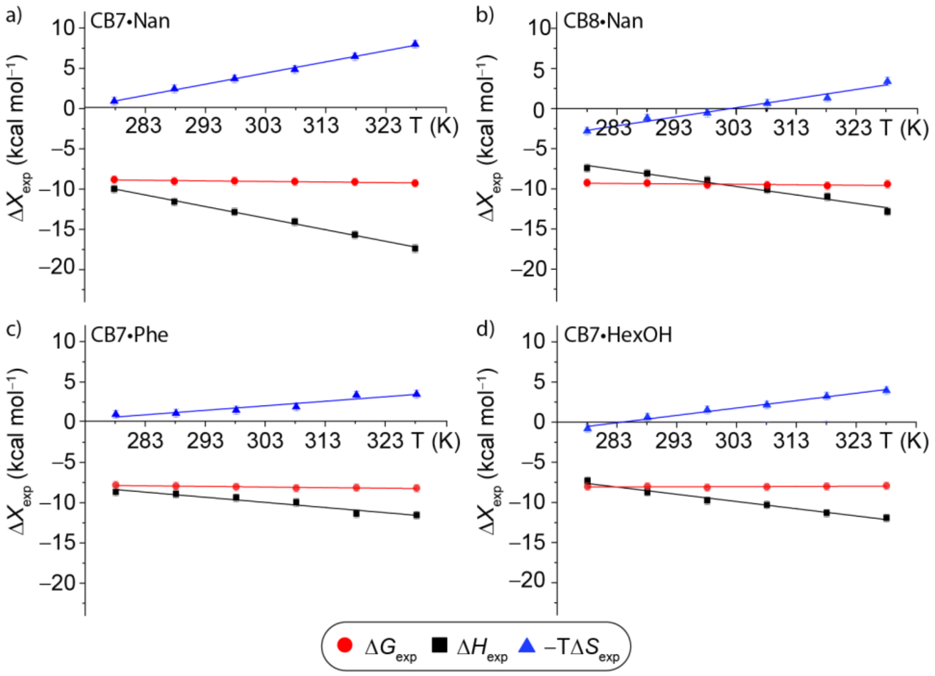

Figure S25. The temperature dependence of the standard complexation parameters for (a) CB7•Nan, (b) CB8•Nan, (c) CB7•Phe, and (d) CB7•HexOH in a temperature range from 278 to 328 K. Error bars calculated as SD from at least three repetition experiments are shown. See **Table S1** - **Table S3** for individual values.

Heat Capacity Changes ($\Delta C_{p,b}$)

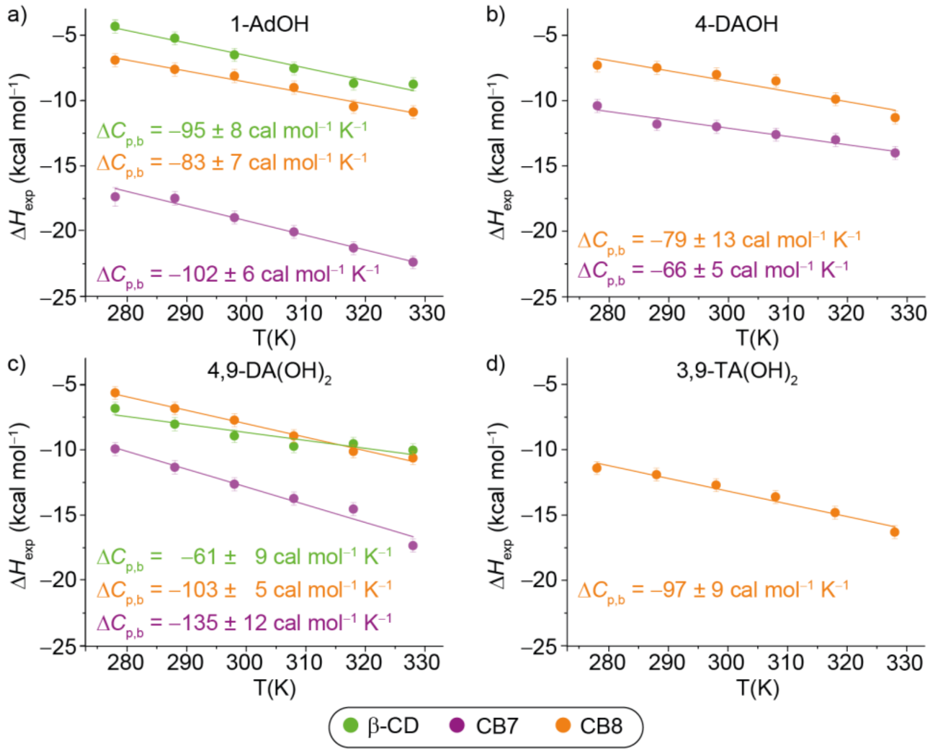

Figure S26. Binding enthalpies as a function of temperature for (a) 1-AdOH, (b) 4-DAOH, (c) 4,9-DA(OH)$_2$, and (d) 3,9-TA(OH)$_2$ with CB7 (violet), CB8 (orange), and β-CD (green). Heat capacity change values (equal to the slope of Δ*H*) are obtained from the linear fits shown. Errors are individual error values of the linear fit.

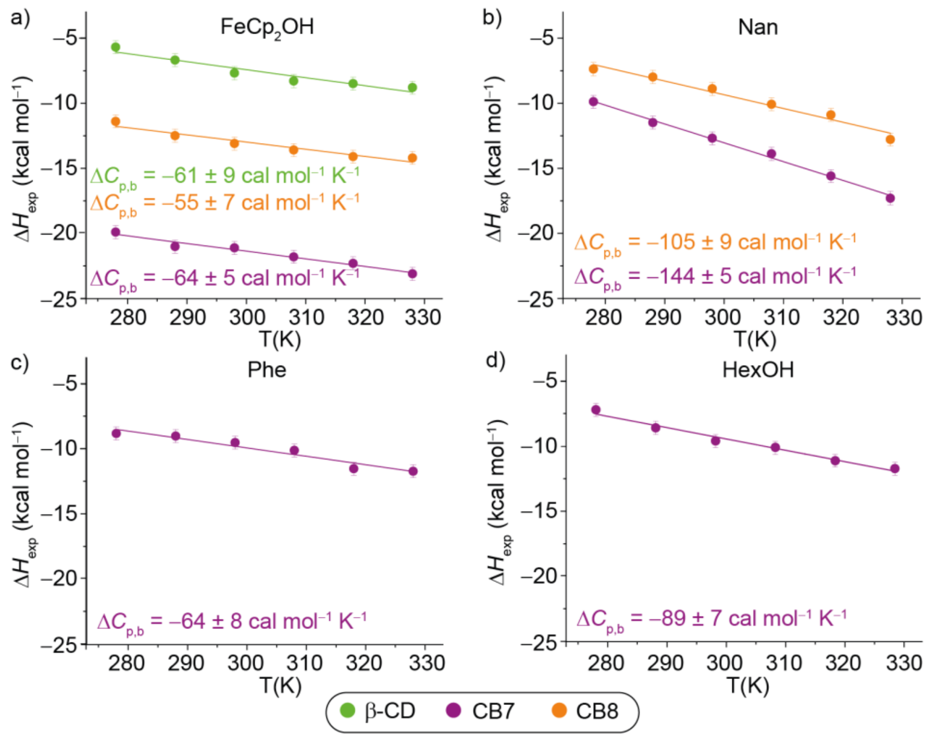

Figure S27. Binding enthalpies as a function of temperature for (a) FeCp$_2$OH, (b) Nan, (c) Phe, and (d) HexOH with CB7 (violet), CB8 (orange), and β-CD (green). Heat capacity change values (equal to the slope of Δ*H*) are obtained from the linear fits shown. Errors are individual error values of the linear fit.

Table S5. Values collected during a literature survey on the entropic ($-\mathrm{T}\Delta S_{\mathrm{exp}}$) and enthalpic ($\Delta H_{\mathrm{exp}}$) contributions to a supramolecular binding event at 298 K as well as the heat capacity change ($\Delta C_{\mathrm{p,b}}$) for protein-ligand interactions in aqueous solution, corresponding to the main text, **Figure 7**. If not stated otherwise, the measurement technique was ITC, and measurements were conducted in pure water at 298 K.

enzyme/ protein	ligand	$\Delta H_{\mathrm{exp}}^{298\mathrm{K}}$ (kcal mol^{-1})	$-\mathrm{T}\Delta S_{\mathrm{exp}}^{298\mathrm{K}}$ (kcal mol^{-1})	$\Delta C_{\mathrm{p,b}}$ (cal mol^{-1} K^{-1})	ref.
aldolase	hexitol-1,6-diphosphate	1.3	–8.5	–1100 ± 200	11[a,b]
avidin	biotin	–22.5 ± 0.1	–0.4	–237 ± 12	12
GPDH	NAD^+	–14.1	5.1	–520	13[a,d]
	NAD^+	–33.6	18.9	–800	13[a,c]
hemoglobin	haptoglobin	–11	21.8	–940	14[l]
heart LDH	NAD^+	–6.1 ± 0.2	–1.0 ± 0.3[l]	–84 ± 8	15[e]
	NADH	–10.6 ± 0.3	3.2 ± 0.4[l]	–169 ± 9	
heart LDH·NAD^+	oxalate	–7.8 ± 0.3	0 ± 0.1	–339 ± 11	
heart LDH·NADH	oxamate	–15.1 ± 0.5	8.4 ± 0.57	–405 ± 21	
muscle LDH	NADH	–7.6 ± 2.1	–0.7 ± 0.63	–325	16[e]
Met(O_2)-S-peptide	S-protein	–34.4 ± 0.9	26.5 ± 0.3	–1140	17[k]
tRNA ligase	L-isoleucine	–4.6 ± 0.9	–3.5 ± 0.2	–427 ± 18	18[f]
	L-leucine	–3.6 ± 0.9	0.9 ± 0.2	–477 ± 28	
	L-valine	–4.0 ± 0.7	–0.7 ± 0.2	–479 ± 24	
	L-norvaline	–7.1 ± 0.7	–0.4 ± 0.2	–372 ± 15	
	L-2-amino-3*S*,4-dimethyl pentanoic acid	–8.2 ± 0.5	–2.1 ± 0.2	–428 ± 19	
	L-isoleucinol	0.6 ± 0.6	–3.7 ± 0.2	–363 ± 35	
RNase A	cytidine 2'-mono-phosphate	–12.6	-	–211	19[g]
		–17.7	-	–200	19[h]
		–10.6	-	–198	19[i]
		–8.5	-	–258	19[j]
S-peptide	S-protein	–39.8 ± 0.9	29.2 ± 0.3	–1460	17[k]
anti-TNP	DNP-lysine	–17.1 ± 0.6	7.9	–205 ± 52	20[l]
	TNP-lysine	–21.3 ± 0.2	9.1	–185 ± 22	

[a] Values given correspond to mole of enzyme. [b] Aqueous solution, pH 7.5. [c] Values given correspond to first site binding; 50 mM pyrophosphate buffer, pH 8.5, containing 2 mM EDTA. [d] 50 mM Phosphate buffer, pH 6.5, containing 2 mM EDTA. [e] Values given correspond to mole of binding site; 200 mM potassium phosphate buffer, pH 7.0. [f] 50 mM Potassium phosphate buffer, pH 7.5, containing 1 mM dithioerythritol. [g] 200 mM Potassium acetate, pH 5.5, containing 200 mM KCl. [h] 50 mM Potassium acetate, pH 5.5. [i] 200 mM Potassium acetate, pH 5.5, containing 200 mM KCl and 500 mM sucrose. [j] 200 mM Potassium acetate, pH 5.5, containing 200 mM KCl and 1 M guanidinium chloride. [k] Aqueous solution containing 300 mM NaCl, pH 7.0. [l] Values were determined at 292 K. DNP = dinitrophenyl; GPDH = glyceraldehyde-3-phosphate dehydrogenase; LDH = lactate dehydrogenase; TNP = trinitrophenyl.

Table S6. Values collected during a literature survey on the entropic ($-\mathrm{T}\Delta S_{\mathrm{exp}}$) and enthalpic ($\Delta H_{\mathrm{exp}}$) contributions to a supramolecular binding event at 298 K as well as the heat capacity changes ($\Delta C_{\mathrm{p,b}}$) for macrocyclic host molecules in aqueous solution, corresponding to the main text, **Figure 7**. Chemical structures of the listed host and guest molecules are shown in **Figure S28**. If not stated otherwise, the measurement technique was ITC, and measurements were conducted in pure water at 298 K.

host	guest	$\Delta H_{\mathrm{exp}}^{298\mathrm{K}}$ (kcal mol^{-1})	$-\mathrm{T}\Delta S_{\mathrm{exp}}^{298\mathrm{K}}$ (kcal mol^{-1})	$\Delta C_{\mathrm{p,b}}$ (cal mol^{-1} K^{-1})	ref.
CATS	1-butylamine$^+$	–4.3 ± 0.2	1.2 ± 0.1	–30 ± 1	21
	1-heptylamine$^+$	–5.0 ± 0.2	–0.4 ± 0.1	–57 ± 4	
	1-hexylamine$^+$	–4.9 ± 0.1	0.04 ± 0.1	–42 ± 4	
	1-pentylamine$^+$	–4.8 ± 0.1	0.4 ± 0.1	–38 ± 4	
	1-propylamine$^+$	–4.0 ± 0.1	1.6 ± 0.1	–21 ± 1	
CB7	$FeCp_2OH$	–21.3 ± 0.5	4.1 ± 0.7	–60 ± 7	22
	$FeCp_2(CH_2N^+Me_3)$	–21.5 ± 0.5	8.6 ± 0.5	–33 ± 7	
	$FeCp_2(CH_2N^+Me_3)_2$	–22.0 ± 0.7	1.0 ± 1.0	–26 ± 7	
α-CD	1-adamantanecarboxylate$^-$	–3.5 ± 0.1	0.1 ± 0.1	–105	23[a]
	adipate^{2-}	–3.6 ± 0.1	0.8 ± 0.1	–67 ± 1	24[a]
	1,4-butanediol	–2.8 ± 0.1	1.6 ± 0.1	–38 ± 11	25
	1-butanol	–2.6 ± 0.1	–0.1 ± 0.1	–83 ± 2	26
	1,10-dodecanediol	–5.9 ± 0.1	0.6 ± 0.1	–101 ± 10	25
	heptandioate^{2-}	–4.5 ± 0.1	0.8 ± 0.1	–76 ± 1	24[a]
	1,7-heptanediol	–5.3 ± 0.1	1.8 ± 0.1	–78 ± 5	25
	1-heptanoate$^-$	–4.2 ± 0.1	0.3 ± 0.1	–84 ± 2	27[b]
	1-heptanol	–5.4 ± 0.3	1.3 ± 0.3	–144 ± 22	26
	1-heptylamine$^+$	–4.8 ± 0.1	0.6 ± 0.1	–92 ± 4	27[b]
	1,6-hexanediol	–4.3 ± 0.1	1.4 ± 0.1	–81 ± 5	25
	1-hexanoate$^-$	–3.5 ± 0.1	0.1 ± 0.1	–74 ± 2	27[b]
	1-hexanol	–4.3 ± 0.3	0.5 ± 0.1	–134 ± 4	26
	1-hexylamine$^+$	–4.2 ± 0.1	0.7 ± 0.1	–78 ± 4	27[b]
	4-nitrophenolate$^-$	–9.3 ± 0.1	5.0 ± 0.1	–26	23[a]
	1,9-nonanediol	–5.4 ± 0.1	0.6 ± 0.1	–89 ± 6	25
	1,5-pentanediol	–4.2 ± 0.1	2.2 ± 0.1	–57 ± 5	25
	1-pentanol	–3.6 ± 0.2	0.3 ± 0.1	–103 ± 3	26
	1-pentylamine$^+$	–3.6 ± 0.1	0.6 ± 0.1	–65 ± 5	27[b]
	1-propanol	–1.6 ± 0.1	–0.2 ± 0.1	–57 ± 3	26
	octanedioate^{2-}	–5.0 ± 0.1	0.6 ± 0.1	–78 ± 1	24[a]
	1,8-octanediol	–5.4 ± 0.1	1.1 ± 0.1	–85 ± 14	25
	1-octylamine$^+$	–5.3 ± 0.1	0.7 ± 0.2	–108 ± 6	27[b]
HP-β-CD	artemisinin	–2.9 ± 0.1	–1.4 ± 0.1	-56	28
	naproxen	–3.2 ± 0.2	–1.9 ± 0.2	-63	28[d]
	naproxenate$^-$	–1.5 ± 0.1	–2.3 ± 0.1	-62	28[e]
β-CD	1-adamantanecarboxylate$^-$	–5.5 ± 0.1	–0.5 ± 0.1	–96	23[a]
	adamantanol	–5.2 ± 0.1	–1.1	–96	29[c]
	benzene	–0.8 ± 0.1	–1.9 ± 0.2	–64 ± 3	30
	cyclohexanol	–1.6 ± 0.1	–2.3 ± 0.1	–79 ± 2	27[b]
	cis-4-methyl-cyclohexanol	–2.3 ± 0.1	–2.0 ± 0.1	–87 ± 2	
	3-(2-hydroxy-phenyl)propionate$^-$	–3.6 ± 0.1	1.0 ± 0.1	–44 ± 6	

β-CD	3-(4-hydroxy-phenyl)propionate^{-}	–3.4 ± 0.1	0.0 ± 0.1	–45 ± 4	27[b]
	4-nitrophenolate^{-}	–4.0 ± 0.1	0.5 ± 0.1	–60	23[a]
	phenethylamine^{+}	–1.5 ± 0.1	–0.4 ± 0.1	–58 ± 1	
	3-phenylbutyrate^{-}	–2.2 ± 0.1	–1.3 ± 0.1	–69 ± 1	27[b]
	3-phenylpropionate^{-}	–1.8 ± 0.1	–1.2 ± 0.1	–61 ± 1	
	tyramine^{+}	–3.3 ± 0.1	0.8 ± 0.1	–36 ± 3	
CP1	isoquinoline	–9.8	3.3	–25	
	1-methyl-1*H*-indole	–1.6	–2.4	–120	
	4-methylquinoline	–9.8	2.7	–130	
	quinoline	–11.0	5.1	–12	
	trimethyladamantanaminium^{+}	–4.7	–2.6	–100	
CP2	isoquinoline	–2.9	–3.3	–61	
	1-methyl-1*H*-indole	0.3	–5.4	–120	31[f]
	4-methylquinoline	1.0	–7.2	–190	
	quinoline	–7.5	1.1	–39	
	trimethyl-adamantan-1-aminium^{+}	–1.3	–4.2	–110	
CP3	trimethyladamantanaminium^{+}	–3.4	–3.0	–130	
CP4	trimethyladamantanaminium^{+}	–4.9	–0.7	–34	
CP5	*p*-cresol	–11.0 ± 0.2	–5.9 ± 0.2	–110 ± 50	
	p-dicyanobenzene	–10.7 ± 0.2	–5.1 ± 0.2	–30	
	p-dimethoxy-benzene	–10.1 ± 0.2	–4.6 ± 0.2	–20	
	dimethyl *p*-benzenedi-carboxylate^{2-}	–11.9 ± 0.2	–5.0 ± 0.2	–60	
	p-dinitrobenzene	–9.9 ± 0.2	–4.6 ± 0.2	–40	32
	hydroquinone	–11.2 ± 0.2	–6.6 ± 0.2	–60	
	p-nitrophenol	–10.9 ± 0.2	–4.6 ± 0.2	–130 ± 20	
	p-nitrotoluene	–8.8 ± 0.2	–2.1 ± 0.2	–50	
	p-tolunitrile	–8.5 ± 0.2	–2.1 ± 0.2	–70	
	p-xylene	–7.5 ± 0.2	–1.9 ± 0.2	–20	
hexa-oxazole 1	hTel24-NMR	–1.70 ± 0.1	–5.8 ± 0.2	–140 ± 3	33[g]
hexa-oxazole 2	hTel24-NMR	–2.1 ± 0.1	–5.7 ± 0.2	–126 ± 4	33[g]
OA	1-decanoate^{-}	–6.5 ± 0.2	–1.4 ± 0.1	–155 ± 2	
	1-heptanoate^{-}	–6.5 ± 0.1	0.2 ± 0.1	–89 ± 2	
	1-hexanoate^{-}	–5.7 ± 0.3	0.6 ± 0.3	–68 ± 1	34
	1-nonanoate^{-}	–6.5 ± 0.1	–1.1 ± 0.1	–133 ± 6	
	1-octanoate^{-}	–6.1 ± 0.1	–0.9 ± 0.1	–116 ± 3	
TEMOA	1-decanoate^{-}	–9.1 ± 0.2	0.1 ± 0.3	–182 ± 3	
	1-heptanoate^{-}	–8.5 ± 0.3	1.2 ± 0.3	–119 ± 7	
	1-hexanoate^{-}	–7.5 ± 0.2	1.5 ± 0.2	–95 ± 6	34
	1-nonanoate^{-}	–9.7 ± 0.3	0.8 ± 0.2	–165 ± 2	
	1-octanoate^{-}	–8.7 ± 0.1	0.3 ± 0.1	–143 ± 3	

[a] Water, pH 9.5. [b] 50 mM Na^{+} buffer pH 6.9. [c] 20 mM HEPES buffer, pH 7.0. [d] Water, pH 2.0. [e] Water, pH 10.0. [f] Van't Hoff plot; T-dependent ^{1}H NMR studies in 10 mM deuterated cesium borate buffer, pD 9.0. [g] 10 mM EPPS, pH 7.5 in the presence of 50 mM K^{+}.

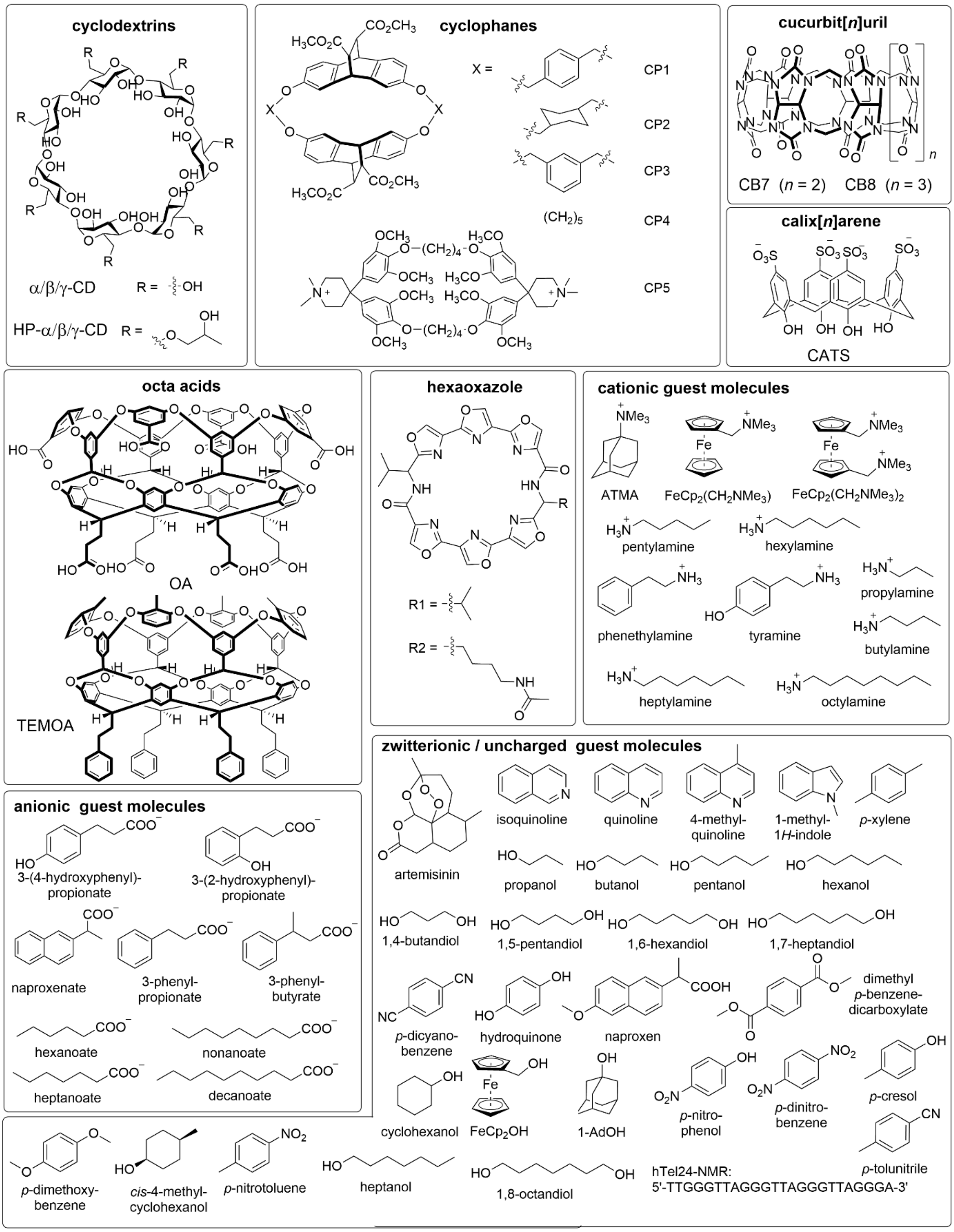

Figure S28. Chemical structures of host and guest molecules corresponding to **Table S5** and **S6**. CATS = 25,26,27,28-tetra-hydroxycalix[4]arene-5,11,17,23-tetrasulfonate; CB*n* = cucurbit[*n*]uril (*n* = 7, 8); CD = cyclodextrin; CP = cyclophane; HP-α/β/γ-CD = hydroxypropyl-α/β/γ-CD; OA = octa acid; TEMOA = tetra-*endo*-methyl octa-acid.

Molecular Dynamics Simulations

Simulation Details. The structures for each host•guest complex were prepared using the build modules of the open-source Python package pAPRika version 1.1.0.[35] The host-guest complex was aligned to the z-axis and solvated with 2500 TIP3P water molecules[36] in a rectangular box. All host•guest complexes were assigned to AM1-BCC partial charges[37, 38] using the antechamber program from AmberTools.[39] General Amber force field (GAFF) 2.1 force field parameters were assigned to each system using the *tLeap* software through the Python wrapper in pAPRika. All MD simulations were performed with OpenMM software version 7.5.1.[40] The temperature was maintained at the desired value using the Langevin thermostat[41] with a collision rate of 1 ps^{-1} and a simulation time step of 2 fs. The pressure was kept at 1 atm with the Monte Carlo barostat.[42] A cutoff of 9 Å was applied when calculating non-bonded interactions, and the PME method was used to calculate long-range electrostatic interactions.[43] An analytical correction was applied to estimate the Lennard-Jones interactions beyond the cutoff distance.[44]

Binding Thermodynamics. The binding free energy ΔG_{calc} was calculated using the attach-pull-release (APR) method,[45] and the binding enthalpy ΔH_{calc} was calculated using the direct method.[46] The addition of noninteracting dummy atoms and configuration of the APR restraints was done with pAPRika. The binding free energy value was obtained using thermodynamic integration (TI), and the uncertainty was estimated using the blocking method.[47] For each APR window, the system was first subjected to energy minimization followed by 2.5 ns of equilibration, and then a 30 ns production run was carried out. For the binding enthalpy ΔH_{calc}, the production runs for the bound and pulled-free states (windows) were simulated for 1 µs. The entropy contribution to the binding free energy, $-\mathrm{T}\Delta S_{\text{calc}}$, was obtained as the difference between ΔG_{calc} and ΔH_{calc}. Confidence intervals (95%) were estimated using 10,000 steps of bootstrapping. The heat capacity change upon binding $\Delta C_{\text{p,b}}$ was obtained from the slope of ΔH_{calc} versus temperature.

We also use the grid inhomogeneous solvation theory (GIST) method[48, 49] to spatially decompose the contributions of water in and around the hosts and guests to $\Delta C_{\text{p,b}}$. Briefly, for a given conformation of a molecule or complex, GIST splits the density-weighted solvation free energy in each voxel i into the solvation energy and solvation entropy contributions,

$$\Delta A_i = \Delta E_i - \mathrm{T}\Delta S_i. \tag{1}$$

The total solvation energy in each voxel is given by

$$\Delta E_i^{tot} = E_i^{sw} + \left(E_i^{ww} - \frac{n_i}{n_{i,\text{bulk}}} E_{i,\text{bulk}}^{ww} \right), \tag{2}$$

where E_i^{sw} and E_i^{ww} are, respectively, the solute-water and water-water energy contributions, n_i is the average number of water molecules in voxel i and $n_{i,bulk}$ is the average number of water molecules that would reside in each voxel if it were in bulk solvent far from the solute(s). The water-water contribution is referenced to bulk $E_{i,\text{bulk}}^{ww}$ in order to make voxels far away from the solutes have zero values. The heat capacity

change in each voxel $C_{\mathrm{p},i}$ is estimated by taking the difference in energy at two temperatures divided by the difference in temperature, *i.e.*,

$$C_{\mathrm{p},i} = \frac{\Delta E_i^{tot}(\mathrm{T}_2) - \Delta E_i^{tot}(\mathrm{T}_1)}{\mathrm{T}_2 - \mathrm{T}_1}. \quad (3)$$

The change in heat capacity upon binding $\Delta C_{\mathrm{p},i}$ is then obtained by subtracting the grid of the free solutes from the identically aligned bound complex grid,

$$\Delta C_{\mathrm{p},i} = C_{\mathrm{p},i}^{complex} - C_{\mathrm{p},i}^{host} - C_{\mathrm{p},i}^{guest}. \quad (4)$$

Calculating the heat capacity change using the equations above with GIST is valid only if the bound complex and the free solutes (host and guest molecules) are all in the same frame of reference. In addition, GIST does not consider the solute-solute interactions, which is valid in this study due to the near-rigidity of the host-guest complexes. The solutes are solvated with 2000 TIP3P water molecules in a cubic box. The conformation of the solutes was chosen from the 1 µs-long simulation used to calculate ΔH_{calc} at 298 K. The positions of the solute atoms were constrained to fix their conformation and simulated for 100 ns at two different temperatures, 298 K and 328 K, respectively. A corresponding water-only system with the same conditions at these two temperatures was simulated to get the reference bulk energy $E_{i,\mathrm{bulk}}^{ww}$ and bulk density ρ^0, 0.0334 $\mathrm{Å}^{-3}$ and 0.0320 $\mathrm{Å}^{-3}$, respectively. The non-PME GPU-accelerated GIST implemented in cpptraj[50] of AmberTools[39] was used to construct a 60x60x60 grid with a spacing of 0.5 Å and the center of mass of the host as the grid center. Before performing the GIST analysis, the free host and guest molecules were aligned to the bound complex structure. The total heat capacity change $\Delta C_{\mathrm{p,b}}$ is obtained by integrating all voxels within 9 Å of any heavy solute atoms.

Hydrogen Bond Analysis. In the hydrogen bond analysis, a hydrogen bond was considered to exist when the donor-acceptor distance is less than 3.0 Å, and the donor-hydrogen-acceptor angle is less than 150°. The MDAnalysis[51, 52] Python package was used to perform the hydrogen bond analysis.

Force Field Comparison

We tested two versions of a well-regarded AMBER force field, GAFF 1.8 and GAFF 2.1, by comparing room temperature computational calorimetry results[45] with the corresponding experimental results for six host-guest complexes defined by guests 1-AdOH and 4,9-DA(OH)$_2$ with hosts CB7, CB8, and β-CD. Both sets of calculations replicate the overall experimental trends, with R^2 values in the order of 0.8, though they overestimate most or all of the binding free energies, enthalpies, and entropies, especially for CB*n* (**Figure S29** and **Table S7**). We sought further insight into the difference between GAFF 1.8 and GAFF 2.1 by comparing the host conformations with the two force fields. As seen in **Figure S30**, both CB7 and CB8 are more flexible with GAFF 1.8 than with GAFF 2.1, as indicated by the larger root-mean-squared deviation (RMSD) relative to an ideal circular conformation. The difference in flexibility becomes more profound with the larger CB8 than with CB7. Such flexibility in the cyclic host may

produce incorrect binding thermodynamics, as demonstrated in a previous study with CD,[53] where the force field that gave a more rigid cyclodextrin showed the best correlation with the experiment, albeit resulting in larger values as observed here as well. A similar issue was observed for CB8 with an initial set of parameters of the AMOEBA polarizable force, and better agreement with the experiment was reached when key torsional parameters were reoptimized to better maintain the host's circular shape during simulations.[54] We also decomposed the binding enthalpy ΔH_{calc} into various force field terms; electrostatics, Lennard-Jones, and valance (**Table S9**). The main difference between the two force fields is the large valence penalties for β-CD with GAFF 1.8, which is consistent with a previous study showing the low degree of preorganization of CDs with earlier versions of GAFF.[53] Therefore, because the calculations with GAFF 2.1 are consistently more accurate than those with the older GAFF 1.8, with lower mean signed errors, root-mean-squared errors, and higher correlation coefficients, we used it to compute the total temperature-dependent binding thermodynamics of the six selected host-guest systems.

Room Temperature Binding Thermodynamics

Table S7. Summary of the calculated (GAFF 1.8 and GAFF 2.1) and experimental binding thermodynamics for the complexation of 1-AdOH and 4,9-DA(OH)$_2$ with CB7, CB8, and β-CD at room temperature (298 K). The binding entropy $-\mathrm{T}\Delta S_{\mathrm{b}}$ is calculated by taking the difference between ΔG_{b} and ΔH_{b}. All energy values are reported in kcal mol^{-1}.

host•guest complex	Force Field	ΔG_{b}		ΔH_{b}		$-\mathrm{T}\Delta S_{\mathrm{b}}$	
		exp	calc	exp	calc	exp	calc
CB7•1-AdOH	GAFF 1.8	–14.2	–23.1	–19.4	–24.0	5.2	0.9
CB7•4,9-DA(OH)$_2$		–9.6	–20.3	–12.6	–22.1	3.0	1.8
CB8•1-AdOH		–9.3	–14.5	–8.1	–7.9	–1.2	–6.6
CB8•4,9-DA(OH)$_2$		–9.9	–20.1	–7.7	–14.0	–2.2	–6.1
β-CD•1-AdOH		–6.5	–4.1	–6.5	–0.2	0.0	–4.3
β-CD•4,9-DA(OH)$_2$		–6.9	–5.4	–8.9	–3.0	2.0	–2.4
CB7•1-AdOH	GAFF 2.1	–14.2	–23.1	–19.4	–25.9	5.2	2.8
CB7•4,9-DA(OH)$_2$		–9.6	–14.6	–12.6	–17.5	3.0	2.9
CB8•1-AdOH		–9.3	–15.2	–8.1	–10.0	–1.2	–5.2
CB8•4,9-DA(OH)$_2$		–9.9	–19.4	–7.7	–15.1	–2.2	–4.3
β-CD•1-AdOH		–6.5	–8.4	–6.5	–7.8	0.0	–0.6
β-CD•4,9-DA(OH)$_2$		–6.9	–9.8	–8.9	–11.2	2.0	1.4

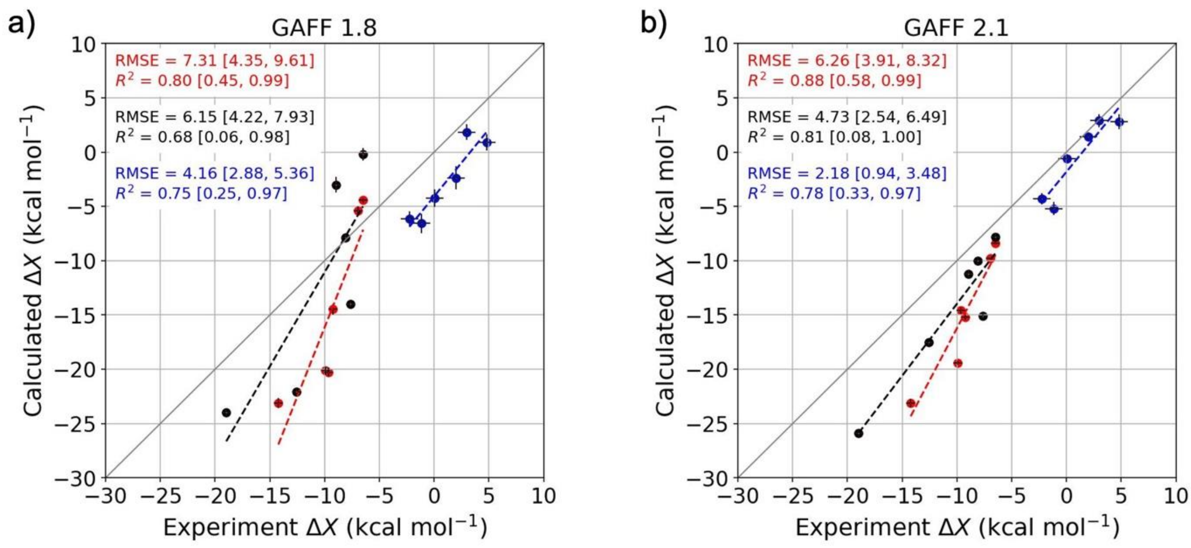

Figure S29. Comparison of the MD-calculated thermodynamic quantities versus experiment at room temperature (298 K) for (a) GAFF 1.8 and (b) GAFF 2.1 force fields. $\Delta G_{\rm b}$ = red, $\Delta H_{\rm b}$ = black, $-\mathrm{T}\Delta S_{\rm b}$ = blue. Values in square brackets give the 95% confidence intervals.

Table S8. Decomposition by energy terms of the calculated (GAFF 1.8 and GAFF 2.1) binding enthalpy $\Delta H_{\rm calc}$ for the complexation of 1-AdOH and 4,9-DA(OH)$_2$ with CB7, CB8, and β-CD at room temperature (298 K). The total change in binding enthalpy $\Delta H_{\rm total}$ is the sum of the Lennard-Jones $\Delta H_{\rm LJ}$, Coulombic electrostatic $\Delta H_{\rm elec}$, and contributions from changes in bond-stretch, angle-bend, and dihedral terms $\Delta H_{\rm val}$. All energy values are reported in kcal mol^{-1} and the typical SEM in $\Delta H_{\rm calc}$ are in the order of 0.2 - 0.6 kcal mol^{-1}.

host•guest complex	Force Field	$\Delta H_{\rm exp}$	$\Delta H_{\rm calc}$			
			$\Delta H_{\rm val}$	$\Delta H_{\rm LJ}$	$\Delta H_{\rm elec}$	$\Delta H_{\rm total}$
CB7•1-AdOH	GAFF 1.8	–19.4	–2.2	–14.5	–7.4	–24.0
CB7•4,9-DA(OH)$_2$		–12.6	0.1	–14.0	–8.2	–22.1
CB8•1-AdOH		–8.1	0.3	–3.4	–4.9	–7.9
CB8•4,9-DA(OH)$_2$		–7.7	0.7	–9.2	–5.5	–14.0
β-CD•1-AdOH		–6.5	6.8	–6.5	–0.5	–0.2
β-CD•4,9-DA(OH)$_2$		–8.9	7.4	–8.9	–1.5	–3.0
CB7•1-AdOH	GAFF 2.1	–19.4	–2.1	–14.4	–9.4	–25.9
CB7•4,9-DA(OH)$_2$		–12.6	2.9	–10.7	–9.7	–17.5
CB8•1-AdOH		–8.1	0.4	–1.7	–8.7	–10.0
CB8•4,9-DA(OH)$_2$		–7.7	0.5	–8.1	–7.4	–15.1
β-CD•1-AdOH		–6.5	–0.6	–4.2	–2.9	–7.8
β-CD•4,9-DA(OH)$_2$		–8.9	0.0	–6.4	–4.8	–11.2

Structure Analysis

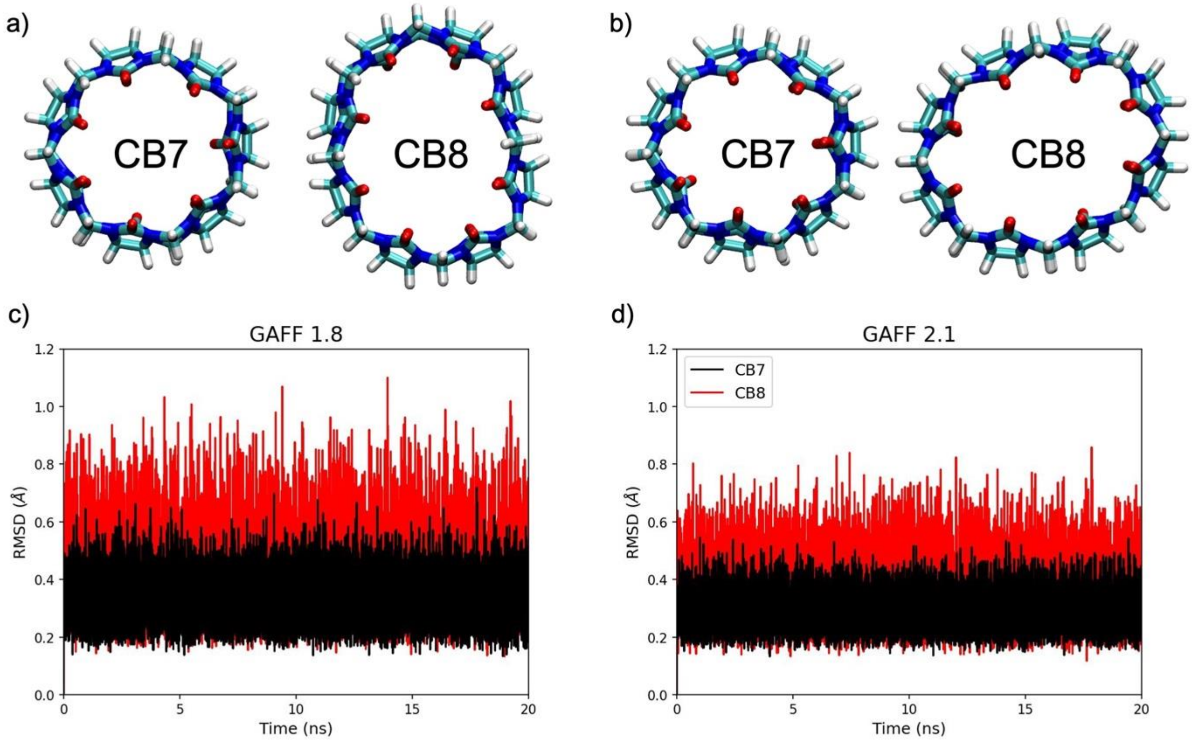

Figure S30. Snapshots of cucurbit[*n*]urils from 20 ns of MD simulations at room temperature (298 K) for (a) GAFF 1.8 and (b) GAFF 2.1 force fields with TIP3P water model. The root-mean-squared-deviation (RMSD) of CB7 (black) and CB8 (red) in reference to a circular structure for (c) GAFF 1.8 and (d) GAFF 2.1 force fields.

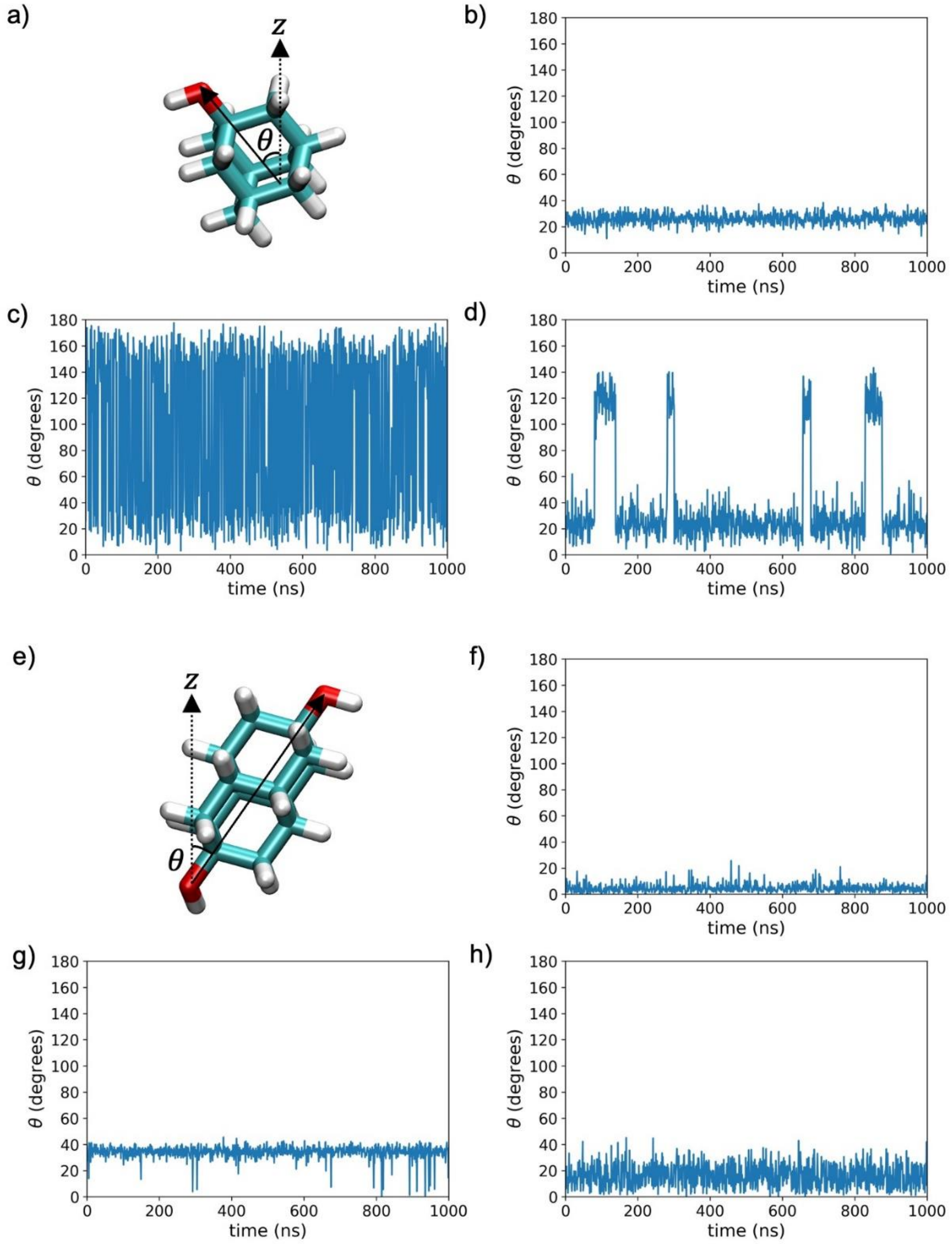

Figure S31. The angle of the normal vector of (a) 1-AdOH and (e) 4,9-DA(OH)$_2$, with respect to the z-axis of the host as a function of time. (b) CB7•1-AdOH, (c) CB8•1-AdOH, (d) β-CD•1-AdOH, (f) CB7•4,9-DA(OH)$_2$, (g) CB8•4,9-DA(OH)$_2$, and (h) β-CD•4,9-DA(OH)$_2$. The analysis is based on MD trajectory at room temperature (298 K).

Temperature Dependence of Binding Thermodynamics

Table S9. Summary of the calculated binding parameters for the complexation of 1-AdOH and 4,9-DA(OH)$_2$ with CB7, CB8, and β-CD in a temperature range from 278 to 328 K. The binding entropy $-\mathrm{T}\Delta S_{\mathrm{calc}}$ was calculated by taking the difference between ΔG_{calc} and ΔH_{calc}, and the uncertainty was determined by adding the SEMs from blocking analysis in quadrature. All energy values are reported in kcal mol^{-1}.

host•guest complex	T(K)	ΔG_{calc}		ΔH_{calc}		$-\mathrm{T}\Delta S_{\mathrm{calc}}$	
		mean	SEM	mean	SEM	mean	SEM
CB7•1-AdOH	278	−22.8	0.2	−23.4	0.2	0.6	0.3
	288	−23.9	0.6	−24.4	0.2	0.5	0.6
	298	−23.1	0.4	−25.9	0.3	2.8	0.5
	308	−23.8	0.2	−27.0	0.2	3.2	0.3
	318	−24.0	0.2	−27.5	0.3	3.5	0.4
	328	−24.4	0.2	−28.9	0.3	4.5	0.4
CB7•4,9-DA(OH)$_2$	278	−14.4	0.3	−15.2	0.3	0.8	0.4
	288	−14.6	0.2	−16.1	0.2	1.5	0.3
	298	−14.6	0.3	−17.5	0.3	2.9	0.4
	308	−14.7	0.2	−18.5	0.2	4.1	0.3
	318	−14.6	0.2	−19.5	0.3	4.9	0.3
	328	−14.7	0.2	−20.3	0.3	5.6	0.3
CB8•1-AdOH	278	−14.2	0.2	−7.8	0.2	−6.4	0.4
	288	−15.0	0.2	−8.7	0.2	−6.3	0.4
	298	−15.2	0.3	−10.0	0.3	−5.2	0.6
	308	−15.6	0.2	−10.2	0.2	−5.4	0.4
	318	−16.0	0.2	−11.0	0.2	−5.0	0.4
	328	−16.4	0.2	−12.0	0.3	−4.4	0.5
CB8•4,9-DA(OH)$_2$	278	−19.0	0.2	−13.1	0.2	−5.9	0.3
	288	−19.6	0.2	−14.4	0.2	−5.2	0.3
	298	−19.4	0.2	−15.1	0.3	−4.3	0.4
	308	−20.3	0.2	−15.7	0.3	−4.6	0.3
	318	−20.7	0.2	−17.4	0.2	−3.3	0.3
	328	−20.8	0.1	−18.2	0.4	−2.6	0.4
β-CD•1-AdOH	278	−8.3	0.1	−6.3	0.2	−2.0	0.2
	288	−8.7	0.2	−7.0	0.2	−1.7	0.3
	298	−8.4	0.1	−7.8	0.3	−0.6	0.3
	308	−9.1	0.1	−8.5	0.2	−0.6	0.2
	318	−9.4	0.2	−9.3	0.3	−0.1	0.3
	328	−10.2	0.3	−10.0	0.2	−0.2	0.4
β-CD•4,9-DA(OH)$_2$	278	−9.5	0.2	−9.2	0.3	−0.3	0.4
	288	−9.8	0.3	−10.1	0.3	0.3	0.4
	298	−9.8	0.1	−11.2	0.3	1.4	0.3
	308	−10.3	0.2	−12.3	0.2	2.0	0.3
	318	−10.3	0.2	−12.8	0.3	2.5	0.3
	328	−10.6	0.2	−13.6	0.3	3.0	0.3

Table S10. Decomposition of the binding enthalpy ΔH_{calc}, following Tang and Chang,[55] over a 278 to 328 K temperature range. The total change in enthalpy upon binding ΔH_{total} is the sum of the host-guest interactions $\Delta H_{H\text{-}G}$ (*i.e.*, solute-solute) and the desolvation energy ΔH_{desolv} (*i.e.*, solute-water and water-water). The enthalpies are calculated from 1 µs of MD simulation and post-processed with MDAnalysis and OpenMM. The binding free energy ΔG_{calc} and binding entropy ΔS_{calc} are also included in the table for comparison (from **Table S10**). All energy values are reported in kcal mol^{-1} and the entropy (ΔS_{calc}) values are reported in cal mol^{-1} K^{-1}.

host•guest complex	T(K)	ΔG_{calc}	ΔH_{calc}			ΔS_{calc}
			$\Delta H_{H\text{-}G}$	ΔH_{desolv}	ΔH_{total}	
CB7•1-AdOH	278	−22.8	−40.5	17.0	−23.5	−2.5
	288	−23.9	−40.4	16.0	−24.4	−1.7
	298	−23.1	−40.4	14.4	−26.0	−9.7
	308	−23.8	−40.4	13.4	−27.0	−10.4
	318	−24.0	−40.3	12.8	−27.5	−11.0
	328	−24.4	−40.3	11.3	−29.0	−14.0
CB7•4,9-DA(OH)$_2$	278	−14.4	−43.0	27.9	−15.1	−2.5
	288	−14.6	−42.9	26.8	−16.1	−5.2
	298	−14.6	−42.8	25.5	−17.4	−9.2
	308	−14.7	−42.8	24.3	−18.5	−12.3
	318	−14.6	−42.6	23.1	−19.5	−15.4
	328	−14.7	−42.5	22.3	−20.2	−16.6
CB8•1-AdOH	278	−14.2	−28.4	20.5	−7.9	22.6
	288	−15.0	−28.4	19.7	−8.7	21.9
	298	−15.2	−28.3	18.2	−10.1	17.1
	308	−15.6	−28.3	18.1	−10.2	17.5
	318	−16.0	−28.3	17.3	−11.0	15.7
	328	−16.4	−28.3	16.2	−12.1	13.1
CB8•4,9-DA(OH)$_2$	278	−19.0	−40.6	27.5	−13.0	21.6
	288	−19.6	−40.6	26.3	−14.3	18.4
	298	−19.4	−40.5	25.5	−15.0	14.8
	308	−20.3	−40.4	24.7	−15.7	14.9
	318	−20.7	−40.4	23.0	−17.4	10.4
	328	−20.8	−40.4	22.3	−18.1	8.2
β-CD•1-AdOH	278	−8.3	−24.0	17.8	−6.3	7.2
	288	−8.7	−23.9	16.9	−7.0	5.9
	298	−8.4	−23.9	16.1	−7.8	2.0
	308	−9.1	−23.9	15.4	−8.5	1.9
	318	−9.4	−24.0	14.7	−9.3	0.3
	328	−10.2	−24.0	14.0	−10.0	0.6
β-CD•4,9-DA(OH)$_2$	278	−9.5	−32.2	23.0	−9.2	1.1
	288	−9.8	−32.0	21.9	−10.1	−1.0
	298	−9.8	−32.0	20.7	−11.2	−4.7
	308	−10.3	−31.8	19.5	−12.3	−6.5
	318	−10.3	−31.7	18.9	−12.8	−7.9
	328	−10.6	−31.6	17.9	−13.6	−9.2

Heat Capacity Changes ($\Delta C_{p,b}$)

Table S11. Comparison of the calculated heat capacity changes ($\Delta C_{p,b}$) upon binding with experiment. Heat capacity changes from MD were obtained from the slope of the calculated enthalpies ΔH_{calc} across a temperature range of 278 to 328 K. The errors were estimated with 10 000 steps of bootstrapping. The GIST-calculated heat capacity changes upon binding were calculated using equations (2)-(4).

host•guest complex	$\Delta C_{p,b}$ (cal mol^{-1} K^{-1})		
	experiment	MD	GIST
CB7•1-AdOH	−102 ± 6	−108 ± 6	−117
CB7•4,9-DA(OH)$_2$	−135 ± 12	−105 ± 7	−110
CB8•1-AdOH	−83 ± 7	−80 ± 6	−89
CB8•4,9-DA(OH)$_2$	−103 ± 5	−100 ± 7	−108
β-CD•1-AdOH	−95 ± 8	−75 ± 10	−90
β-CD•4,9-DA(OH)$_2$	−61 ± 9	−89 ± 7	−86

Table S12. Decomposition of the MD-calculated heat capacity changes ($\Delta C_{p,b}^{MD}$) upon binding. The total $\Delta C_{p,total}$ is decomposed into the sum of the host-guest $\Delta C_{p,H\text{-}G}$ (solute-solute) and desolvation $\Delta C_{p,desolv}$ (solute-water and water-water) interactions based on the enthalpy values in **Table S11**.

host•guest complex	$\Delta C_{p,b}^{MD}$ (cal mol^{-1} K^{-1})		
	$\Delta C_{p,H\text{-}G}$	$\Delta C_{p,desolv}$	$\Delta C_{p,total}$
CB7•1-AdOH	5	−113	−108
CB7•4,9-DA(OH)$_2$	10	−115	−105
CB8•1-AdOH	3	−83	−80
CB8•4,9-DA(OH)$_2$	5	−105	−100
β-CD•1-AdOH	0	−75	−75
β-CD•4,9-DA(OH)$_2$	11	−100	−89

Table S13. Decomposition of the GIST-calculated heat capacity changes ($\Delta C_{p,b}^{GIST}$) upon binding. The total $\Delta C_{p,total}$ is decomposed into the sum of voxels inside, $\Delta C_{p,cavity}$, and outside the cavity, $\Delta C_{p,portal}$. The heat capacity change inside the cavity is defined as the integral of a cylindrical volume with a radius of 7 Å and a height of $\pm$3.2 Å from the center of the host. The heat capacity change outside of the cavity is obtained by subtracting the cavity value from the total.

host•guest complex	$\Delta C_{p,b}^{GIST}$ (cal mol^{-1} K^{-1})		
	$\Delta C_{p,cavity}$	$\Delta C_{p,portal}$	$\Delta C_{p,total}$
CB7•1-AdOH	−68	−49	−117
CB7•4,9-DA(OH)$_2$	−64	−46	−110
CB8•1-AdOH	−58	−31	−89
CB8•4,9-DA(OH)$_2$	−60	−48	−108
β-CD•1-AdOH	−81	−9	−90
β-CD•4,9-DA(OH)$_2$	−81	−5	−86

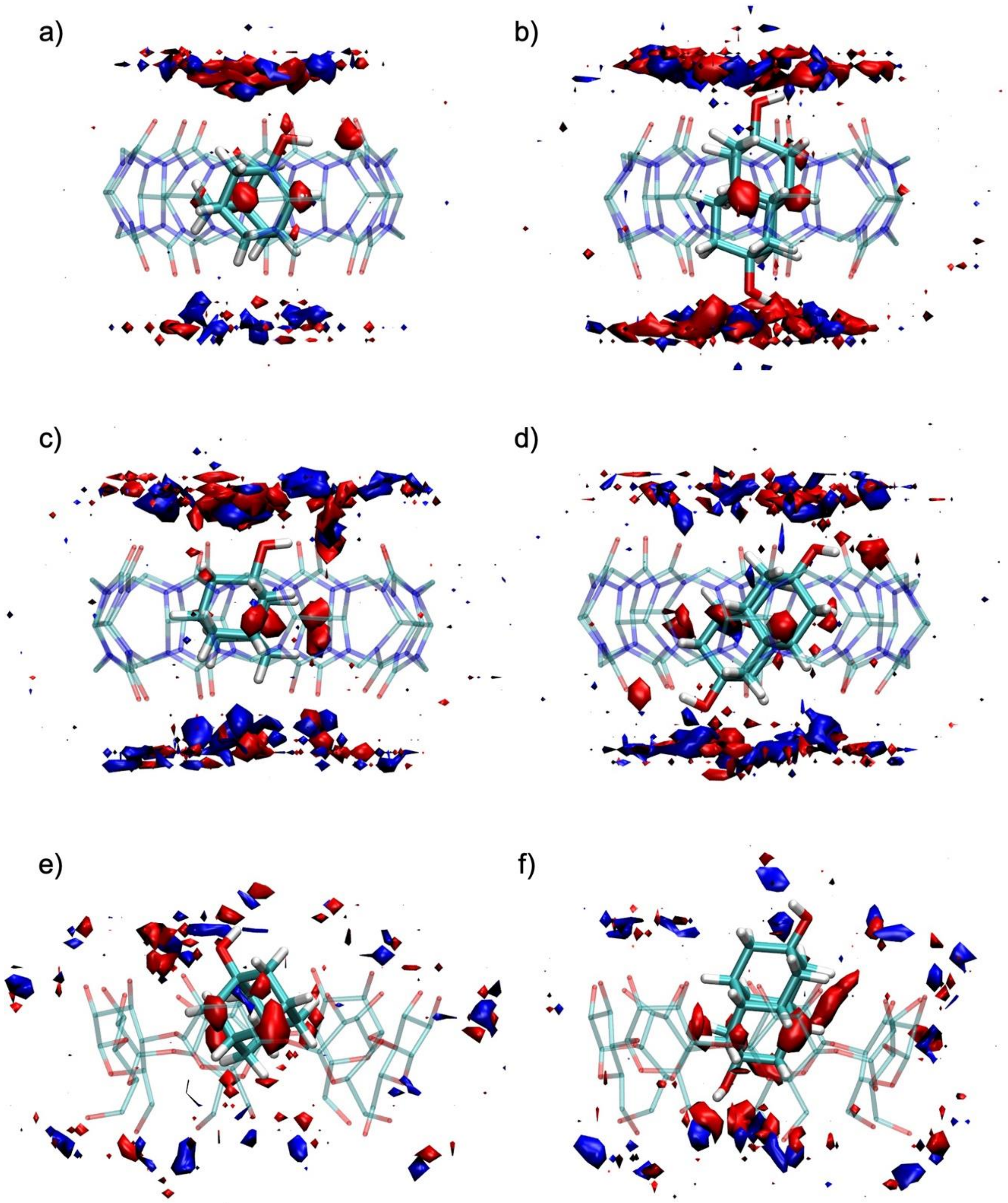

Figure S32. GIST contour plot for the heat capacity change ($\Delta C_{\mathrm{p,b}}$) upon binding for (a) CB7•1-AdOH, (b) CB7•4,9-DA(OH)$_2$, (c) CB8•1-AdOH, (d) CB8•4,9-DA(OH)$_2$, (e) β-CD•1-AdOH, and (f) β-CD•4,9-DA(OH)$_2$. The blue and red contours are 0.2 cal mol^{-1} K^{-1} and –0.2 cal mol^{-1} K^{-1}, respectively.

References

1. C. Marquez, F. Huang and W. Nau, *NanoBioscience, IEEE Transactions on*, 2004, **3**, 39-45.
2. D. Jiao and O. A. Scherman, *Green Chem.*, 2012, **14**, 2445-2449.
3. K. Gao, J. Yin, N. M. Henriksen, A. T. Fenley and M. K. Gilson, *J. Chem. Theroy Comput.*, 2015, **11**, 4555-4564.
4. N. A. Fokina, B. A. Tkachenko, A. Merz, M. Serafin, J. E. P. Dahl, R. M. K. Carlson, A. A. Fokin and P. R. Schreiner, *Eur. J. Org. Chem.*, 2007, **2007**, 4738-4745.
5. M. T. H. Khan, *Bioactive Heterocycles IV*, Springer-Verlag, Berlin Heidelberg, 2007.
6. S. Yi and A. E. Kaifer, *J. Org, Chem.*, 2011, **76**, 10275-10278.
7. W. S. Jeon, K. Moon, S. H. Park, H. Chun, Y. H. Ko, J. Y. Lee, E. S. Lee, S. Samal, N. Selvapalam, M. V. Rekharsky, V. Sindelar, D. Sobransingh, Y. Inoue, A. E. Kaifer and K. Kim, *J. Am. Chem. Soc.*, 2005, **127**, 12984-12989.
8. S. Sinn, E. Spuling, S. Bräse and F. Biedermann, *Chem. Sci.*, 2019, **10**, 6584-6593.
9. S. Sinn, J. Krämer and F. Biedermann, *Chem. Commun.*, 2020, **56**, 6620-6623.
10. L. M. Grimm, S. Spicher, B. Tkachenko, P. R. Schreiner, S. Grimme and F. Biedermann, *Chem. Eur. J.*, 2022, **28**, e202200529.
11. J. M. Sturtevant, H. J. Hinz and D. D. Shiao, *Biochemistry*, 1971, **10**, 1347-1352.
12. J. Suurkuusk and I. Wadsö, *Eur. J. Biochem.*, 1972, **28**, 438-441.
13. C. W. Niekamp, J. M. Sturtevant and S. F. Velick, *Biochemistry*, 1977, **16**, 436-445.
14. F. Lavialle, M. Rogard and A. Alfsen, *Biochemistry*, 1974, **13**, 2231-2234.
15. F. Schmid, H. J. Hinz and R. Jaenicke, *Biochemistry*, 1976, **15**, 3052-3059.
16. H. J. Hinz and R. Jaenicke, *Biochemistry*, 1975, **14**, 24-27.
17. J. M. Sturtevant, R. P. Hearn, F. M. Richards and G. D. Watt, *Biochemistry*, 1971, **10**, 806-817.
18. H. J. Hinz, K. Weber, J. Flossdorf and M. R. Kula, *Eur. J. Biochem.*, 1976, **71**, 437-442.
19. H. Naghibi, A. Tamura and J. M. Sturtevant, *PNAS*, 1995, **92**, 5597-5599.
20. B. Barisas, S. Singer and J. Sturtevant, *Biochemistry*, 1972, **11**, 2741-2744.
21. M. Stödeman and N. Dhar, *J. Chem. Soc., Faraday Trans.*, 1998, **94**, 899-903.
22. V. Rekharsky Mikhail, T. Mori, C. Yang, H. Ko Young, N. Selvapalam, H. Kim, D. Sobransingh, E. Kaifer Angel, S. Liu, L. Isaacs, W. Chen, S. Moghaddam, M. K. Gilson, K. Kim and Y. Inoue, *PNAS*, 2007, **104**, 20737-20742.
23. J. C. Harrison and M. R. Eftink, *Biopolymers*, 1982, **21**, 1153-1166.
24. I. Gómez-Orellana, D. Hallén and M. Stödeman, *J. Chem. Soc., Faraday Trans.*, 1994, **90**, 3397-3400.
25. M. Bastos, L. E. Briggner, I. Shehatta and I. Wadsö, *J. Chem. Thermodyn.*, 1990, **22**, 1181-1190.
26. D. Hallén, A. Schön, I. Shehatta and I. Wadsö, *J. Chem. Soc., Faraday Trans.*, 1992, **88**, 2859-2863.
27. P. D. Ross and M. V. Rekharsky, *Biophys. J.*, 1996, **71**, 2144-2154.
28. A. C. Illapakurthy, C. M. Wyandt and S. P. Stodghill, *Eur. J. Pharm. Biopharm.*, 2005, **59**, 325-332.
29. B. Zhang and R. Breslow, *J. Am. Chem. Soc.*, 1993, **115**, 9353-9354.
30. I. Gómez-Orellana and D. Hallén, *Thermochim. Acta*, 1993, **221**, 183-193.
31. D. A. Stauffer, R. E. Barrans Jr and D. A. Dougherty, *J. Org, Chem.*, 1990, **55**, 2762-2767.
32. D. B. Smithrud, T. B. Wyman and F. Diederich, *J. Am. Chem. Soc.*, 1991, **113**, 5420-5426.
33. D. S. Pilch, C. M. Barbieri, S. G. Rzuczek, E. J. LaVoie and J. E. Rice, *Biochimie*, 2008, **90**, 1233-1249.

34. J. W. Barnett, M. R. Sullivan, J. A. Long, D. Tang, T. Nguyen, D. Ben-Amotz, B. C. Gibb and H. S. Ashbaugh, *Nat. Chem.*, 2020, **12**, 589-594.
35. D. Slochower, Setiadi, J., Henriksen, N., Boothroyd, S., Thompson, M., & Wang, L., *pAPRika version 1.1.0 https://github.com/slochower/pAPRika*, 2022.
36. W. L. Jorgensen, J. Chandrasekhar, J. D. Madura, R. W. Impey and M. L. Klein, *J. Chem. Phys.*, 1983, **79**, 926-935.
37. A. Jakalian, B. L. Bush, D. B. Jack and C. I. Bayly, *J. Comput. Chem.*, 2000, **21**, 132-146.
38. A. Jakalian, D. B. Jack and C. I. Bayly, *J. Comput. Chem.*, 2002, **23**, 1623-1641.
39. D. A. Case, H. M. Aktulga, K. Belfon, I. Ben-Shalom, S. R. Brozell, D. S. Cerutti, T. E. Cheatham III, V. W. D. Cruzeiro, T. A. Darden and R. E. Duke, *Amber 2021*, University of California, San Francisco, 2021.
40. P. Eastman, J. Swails, J. D. Chodera, R. T. McGibbon, Y. Zhao, K. A. Beauchamp, L.-P. Wang, A. C. Simmonett, M. P. Harrigan and C. D. Stern, *PLoS Comput. Biol.*, 2017, **13**, e1005659.
41. R. J. Loncharich, B. R. Brooks and R. W. Pastor, *Biopolymers: Original Research on Biomolecules*, 1992, **32**, 523-535.
42. J. Åqvist, P. Wennerström, M. Nervall, S. Bjelic and B. O. Brandsdal, *Chem. Phys. Lett.*, 2004, **384**, 288-294.
43. T. Darden, D. York and L. Pedersen, *J. Chem. Phys.*, 1993, **98**, 10089-10092.
44. T. Steinbrecher, D. L. Mobley and D. A. Case, *J. Chem. Phys.*, 2007, **127**, 214108.
45. N. M. Henriksen, A. T. Fenley and M. K. Gilson, *J. Chem. Theroy Comput.*, 2015, **11**, 4377-4394.
46. A. T. Fenley, N. M. Henriksen, H. S. Muddana and M. K. Gilson, *J. Chem. Theroy Comput.*, 2014, **10**, 4069-4078.
47. H. Flyvbjerg and H. G. Petersen, *J. Chem. Phys.*, 1989, **91**, 461-466.
48. S. Ramsey, C. Nguyen, R. Salomon-Ferrer, R. C. Walker, M. K. Gilson and T. Kurtzman, *J. Comput. Chem.*, 2016, **37**, 2029-2037.
49. C. N. Nguyen, T. K. Young and M. K. Gilson, *Chem. Phys.*, 2012, **137**, 044101.
50. D. R. Roe and T. E. Cheatham, III, *J. Chem. Theroy Comput.*, 2013, **9**, 3084-3095.
51. Richard J. Gowers, Max Linke, Jonathan Barnoud, Tyler J. E. Reddy, Manuel N. Melo, Sean L. Seyler, Jan Domanski, David L. Dotson, Sebastian Buchoux, Ian M. Kenney and O. Beckstein, *MDAnalysis: A Python Package for the Rapid Analysis of Molecular Dynamics Simulations*, 2016.
52. N. Michaud-Agrawal, E. J. Denning, T. B. Woolf and O. Beckstein, *J. Comput. Chem.*, 2011, **32**, 2319-2327.
53. D. R. Slochower, N. M. Henriksen, L.-P. Wang, J. D. Chodera, D. L. Mobley and M. K. Gilson, *J. Chem. Theroy Comput.*, 2019, **15**, 6225-6242.
54. M. L. Laury, Z. Wang, A. S. Gordon and J. W. Ponder, *J. Comput. Aided Mol. Des.*, 2018, **32**, 1087-1095.
55. Z. Tang and C.-e. A. Chang, *J. Chem. Theroy Comput.*, 2018, **14**, 303-318.
